# Discriminating agonist and antagonist ligands of the nuclear receptors using 3D-pharmacophores

**DOI:** 10.1186/s13321-016-0154-2

**Published:** 2016-09-06

**Authors:** Nathalie Lagarde, Solenne Delahaye, Jean-François Zagury, Matthieu Montes

**Affiliations:** Laboratoire Génomique Bioinformatique et Applications, Équipe d’accueil EA 4627, Conservatoire National des Arts et Métiers, 292 rue Saint Martin, 75003 Paris, France

**Keywords:** Nuclear receptors, Agonist ligands, Antagonist ligands, Pharmacophores, Structure-based, Ligand-based, Virtual screening

## Abstract

Nuclear receptors (NRs) constitute an important class of therapeutic targets. We evaluated the performance of 3D structure-based and ligand-based pharmacophore models in predicting the pharmacological profile of NRs ligands using the NRLiSt BDB database. We could generate selective pharmacophores for agonist and antagonist ligands and we found that the best performances were obtained by combining the structure-based and the ligand-based approaches. The combination of pharmacophores that were generated allowed to cover most of the chemical space of the NRLiSt BDB datasets. By screening the whole NRLiSt BDB on our 3D pharmacophores, we demonstrated their selectivity towards their dedicated NRs ligands. The 3D pharmacophores herein presented can thus be used as a predictor of the pharmacological activity of NRs ligands.

**Electronic supplementary material:**

The online version of this article (doi:10.1186/s13321-016-0154-2) contains supplementary material, which is available to authorized users.

**Graphical Abstract:**
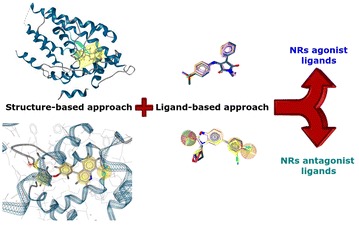
Using a combination of structure-based and ligand-based pharmacophores, agonist and antagonist ligands of the Nuclear Receptors included in the NRLiSt BDB database could be separated

## Background

Nuclear receptors (NRs) are involved in a wide range of physiological key functions. They are potential targets for numerous diseases and constitute an important class of therapeutic targets [1, 2]. NRs are transcription factors naturally switched on and off by small-molecule hormones, and artificially by synthetic ligands. Taking advantage of the biological potency of the NRs, a large amount of compounds has been proposed to modulate their activity and some of them are still marketed [3, 4]. The NRs ligands can be classified according to their pharmacological profiles, the two main classes being agonist and antagonist ligands. These two classes of compounds act through the binding to a NR and the activation (agonist ligands) or the inhibition (antagonist ligands) of its activity. The drug discovery process is thus not limited to the search of the best ligand of a given target, but consists in the search of a ligand with a pharmacological profile that his compatible to the required activity. In this context, the ability to predict the agonist or antagonist behaviour of a NR ligand is of major importance. In recent years, virtual screening methods have proven their ability to predict the activity of small compounds [5–7] and can be used to predict the pharmacological profile of NRs ligands. Numerous ligand-based (LB) and structure-based (SB) virtual screening studies dedicated to NRs were conducted but only few focused on the agonism/antagonism issues [8–17]. Despite these several prediction attempts and the elucidation of the molecular bases of agonism and antagonism [18–21], discriminating agonist from antagonist ligands based on their sole structure remains a challenge. In this study, we describe a 3D pharmacophore modeling study performed on 27 NRs, with the aim to provide separate and selective agonist and antagonist pharmacophores for each NR. To our knowledge, this is the first large-scale study conducted to predict the agonist and antagonist behaviour of NRs ligands using a 3D pharmacophore modeling method. 3D pharmacophores are nowadays widely used as filters in virtual screening protocols and several studies successfully identified new NRs ligands using pharmacophore models [22–30]. Pharmacophore models display two main advantages: reduced computational times associated to the simplified pharmacophoric representations and a large diversity of potential hits with scaffolds and functional groups distinct to the original ligands [31, 32]. To design our study, we used the 27 NRLiSt BDB datasets [33]. For each dataset, we created both SB and LB 3D pharmacophores and compared the ability of these two approaches to generate agonist selective pharmacophores and antagonist selective pharmacophores covering the whole NRLiSt BDB ligands chemical space. We also studied the performance obtained using a combination of SB and LB pharmacophores and analyzed the composition and the selectivity against all NRs datasets of these combinations. In the present study, we describe our attempt to develop selective pharmacophores for agonist or antagonist ligands that could be used to predict the pharmacological activity of NRs.

## Methods

### Nuclear receptors ligands and structures benchmarking DataBase (NRLiSt BDB)

The NRLiSt BDB [33] is a freely available benchmarking database for both SB and LB methods evaluation and dedicated to the NRs. The NRLiSt BDB presents separated agonist and antagonist datasets for the 27 targets (out of the 48 known NRs) for which more than one agonist ligand, one antagonist ligand, and at least one experimental structure was available. All of the ligands found to be agonist or antagonist in the scientific literature are provided in two separated datasets and all of the available human holo PDB structures (except for RXR_gamma, for which only one apo structure was available). A total of 7853 actives, 458,981 decoys, and 339 structures are divided into 54 datasets. The NRLiSt BDB was downloaded from the Web site http://nrlist.drugdesign.fr.

### LigandScout

3D pharmacophores were generated using the software LigandScout [34] (version 4.0) in SB and LB approaches.

#### Structure-based approach

3D SB pharmacophores were automatically generated using the PDB structures included in the NRLiSt BDB. This approach is only possible with holo structures, thus no RXR gamma 3D SB pharmacophore could be computed. In this approach, the LigandScout algorithm tags the key features of the ligand that are interacting with the residues of the receptor: aromatic ring, hydrophobic area, hydrogen bond donor or acceptor, negative or positive ionisable atom and metal binding location. To complete the pharmacophore, an ensemble of exclusion volume spheres is generated to represent the shape of the active site.

#### Ligand-based approach

All ligands of each dataset were clustered with LigandScout using default settings except for the cluster distance that was adjusted for each NR to obtain balanced clusters. For each cluster, a 3D LB pharmacophore was generated using the “merged feature pharmacophore approach” with the number of omitted features for a given merged pharmacophore set to 4 and optional partially matching features with a threshold set to 10 %. In this approach, all the features observed in each ligand of the training datasets are identified, scored and removed according to the threshold number of omitted features. We chose to enable the creation of exclusion volume spheres around the alignment of ligands. In some cases, we added manually exclusion volume spheres to remove decoys compounds since inactive compounds can map all the pharmacophore require features, their inactivity being explained by steric clashes with the binding site [35, 36]. For each pharmacophore, the ligands of the cluster used to generate the pharmacophore constituted the training set and the test set was formed by all agonist ligands and all antagonist ligands of the corresponding NR. During the pharmacophore generation, the ligands of the training set were automatically aligned with the LigandScout pharmacophore-based alignment algorithm [37].

#### Model optimization protocol

**Fig. 1:**
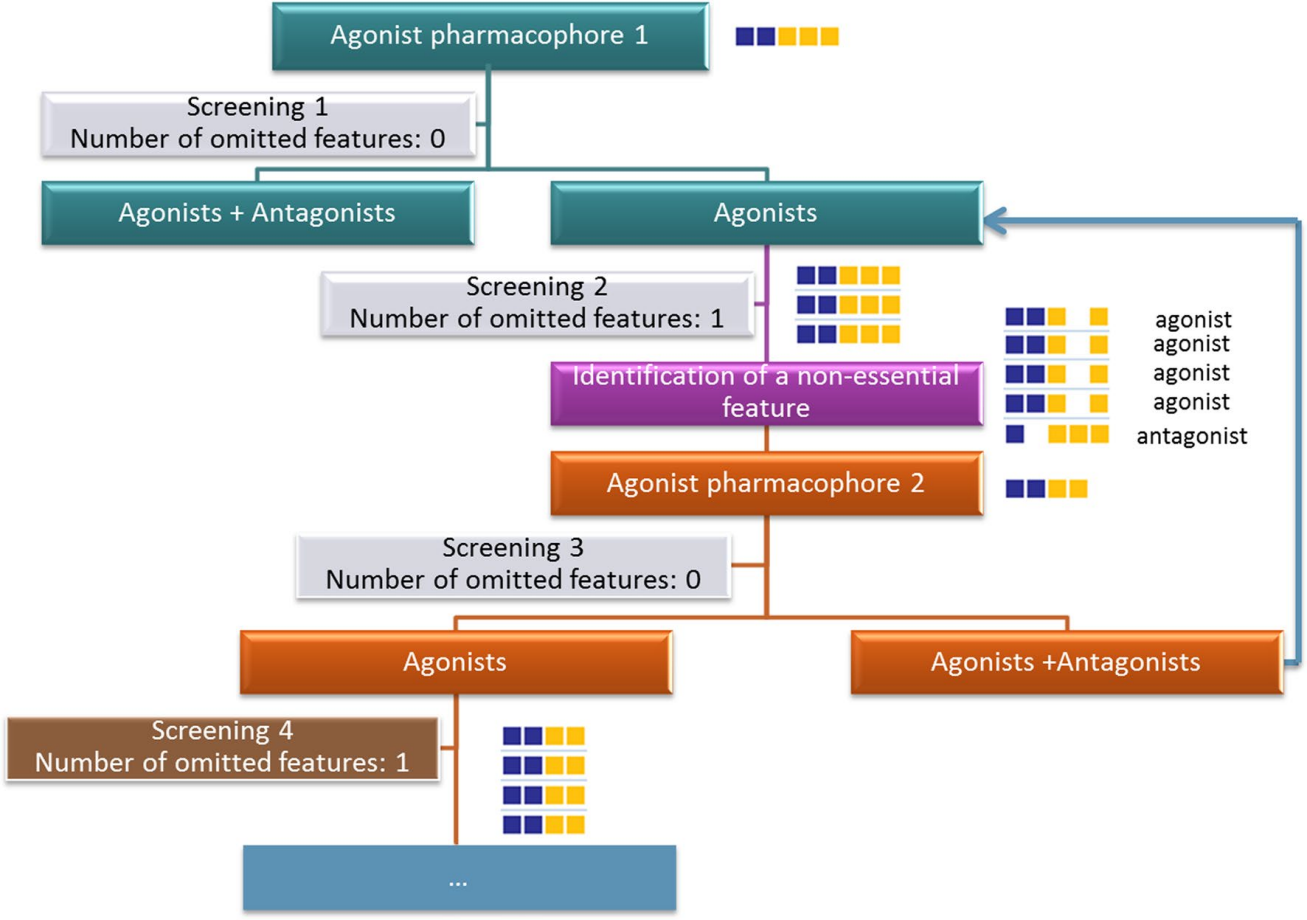
Screening protocol developed to select selective pharmacophores for agonist ligands and selective pharmacophores for antagonist ligands using LigandScout. The example presented here started with a pharmacophore generated with agonist ligands containing 2 aromatic rings (*blue square*) and 3 hydrophobic groups (*yellow square*). The pharmacophore was retained if only agonist ligands were retrieved in the screening 1 (with the Max. number of omitted features set to 0, which means that only ligands mapping the 5 pharmacophore features are considered as hits). Then, we tried to identify non-essential feature in the screening 2 by setting the Max. number of omitted features to 1 (which means that the ligands mapping 4 of the 5 pharmacophore features are considered as hits). A second agonist pharmacophore was defined by disabling the pharmacophore feature identified as non-essential. The agonist pharmacophore 2 was validated if the hits of the screening 3 (with the Max. number of omitted features set to 0) were only agonist ligands. This protocol was applied to each pharmacophore until 3 pharmacophore features were retained or until no non-essential feature could be identified

The generated 3D pharmacophores were used to screen the NRs datasets. All of the ligands provided in SMILES format in the NRLiSt BDB were converted in .ldb format using the idbgen tool provided with LigandScout with the omega-fast option. Two databases were used for each screening, a screening database of active compounds and a screening database of decoys. Agonist ligands were used as decoys for antagonist pharmacophores and reciprocally antagonist ligands were used as decoys for agonist pharmacophores. We developed an original model optimization protocol for this study (Fig. 1), to sequentially refine the pharmacophore models according to several literature recommendations [32, 38, 39]. For each pharmacophore, a first screening was made with LigandScout default settings and particularly the Max. number of omitted features set to 0. If the hits retrieved with this first screening contained both agonist and antagonist ligands, the pharmacophore was not validated and was not retained. If only agonist or antagonist ligands were retrieved in this first screening, the pharmacophore was validated and a second screening was performed with this pharmacophore, but with the Max. number of omitted features parameter set to 1. This second screening was carried out to identify possible non-essential pharmacophore features, i.e. features that can be disabled to obtain less stringent pharmacophores able to retrieve more active ligands (agonist ligands when using agonist datasets or antagonist ligands when using antagonists datasets), but no decoys (antagonists when using agonist datasets and agonists when using antagonist datasets). When a non-essential pharmacophore feature was identified, a third screening was performed with the non-essential feature marked as disabled and the Max. number of omitted features parameter set to 0. If the hits retrieved with this third screening were both agonist and antagonist ligands, this second pharmacophore was not validated and another round of identification of non-essential features was performed. If only active ligands were retrieved, the pharmacophore was validated and other non-essential features were studied. This protocol was applied to each pharmacophore until 3 pharmacophore features were retained or until no non-essential feature could be identified.

#### Combination of SB pharmacophores, combination of LB pharmacophores and combination of SBLB pharmacophores

Using the SB approach, for each NR, all the selective pharmacophores generated, i.e. all pharmacophores that retrieved only agonist ligands or antagonist ligands, were gathered into two groups: “SB agonist selective pharmacophores” and “SB antagonist selective pharmacophores”; redundant pharmacophores were removed. Similarly, all the selective pharmacophores obtained with the LB approach for each NR were gathered into two groups: “LB agonist selective pharmacophores” and “LB antagonist selective pharmacophores”; redundant pharmacophores were removed. Finally, the SB and LB selective pharmacophores previously generated were gathered in two pharmacophore ensembles: “SBLB agonist selective pharmacophores” and “SBLB antagonist selective pharmacophores”; redundant pharmacophores were removed.

Redundant pharmacophores are pharmacophores that could be removed without decreasing the recall of the set of combined pharmacophores i.e. pharmacophores that only retrieved ligands that were also retrieved with other pharmacophores of the set. To remove these redundant pharmacophores, all generated pharmacophores were ranked according to the number of hits they retrieved. Then, each pharmacophore was removed sequentially, starting from the pharmacophore associated with the smallest number of hits. For each removal, the impact on the recall was evaluated. If the recall was not affected, the pharmacophore was dismissed and in the opposite, if the recall decreased, the pharmacophore was conserved.

### Performance metrics

**Fig. 2:**
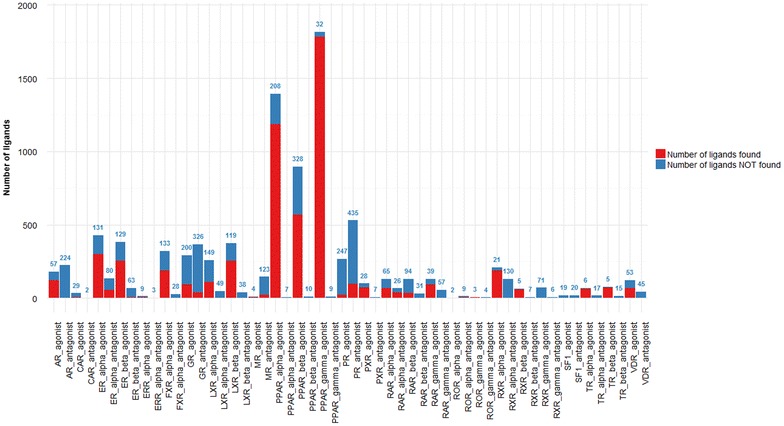
Performances of the structure-based approach for each NRLiSt BDB dataset. The amount of ligands retrieved during the virtual screening procedure using structure-based selective pharmacophores is shown in *red* whereas the number of ligands not covered by the pharmacophores is depicted and labelled in *blue*

**Fig. 3:**
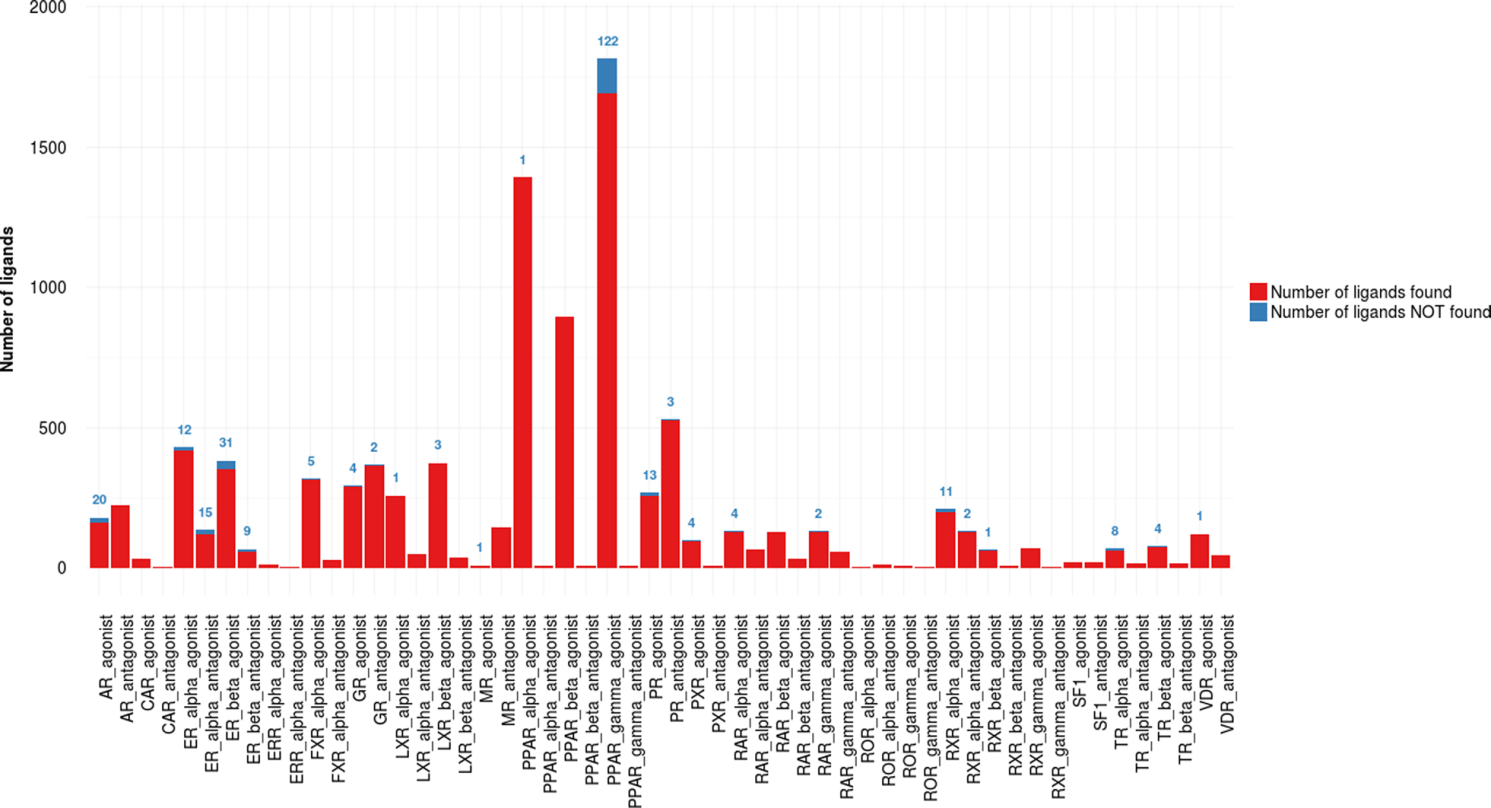
Performances of the ligand-based approach for each NRLiSt BDB dataset. The amount of ligands retrieved during the virtual screening procedure using ligand-based selective pharmacophores is shown in *red* whereas the number of ligands not covered by the pharmacophores is depicted and labelled in *blue*

**Fig. 4:**
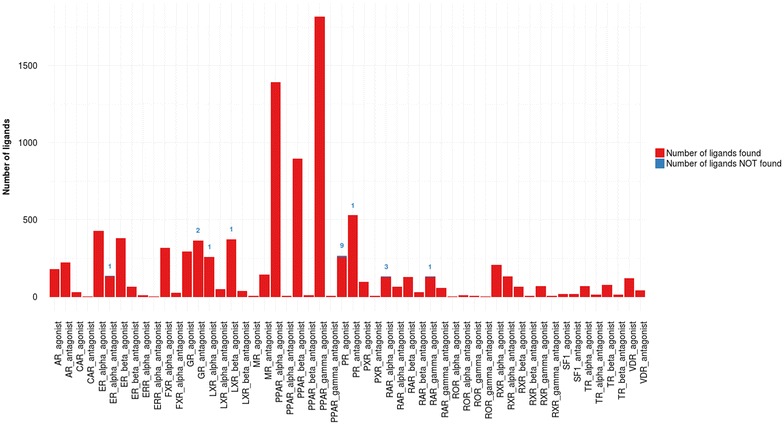
Performances of the combination of structure-based and ligand-based approach for each NRLiSt BDB dataset. The amount of ligands retrieved during the virtual screening procedure using the combination of structure-based and ligand-based selective pharmacophores is shown in *red* whereas the number of ligands not covered by the pharmacophores is depicted and labelled in *blue*

**Fig. 5:**
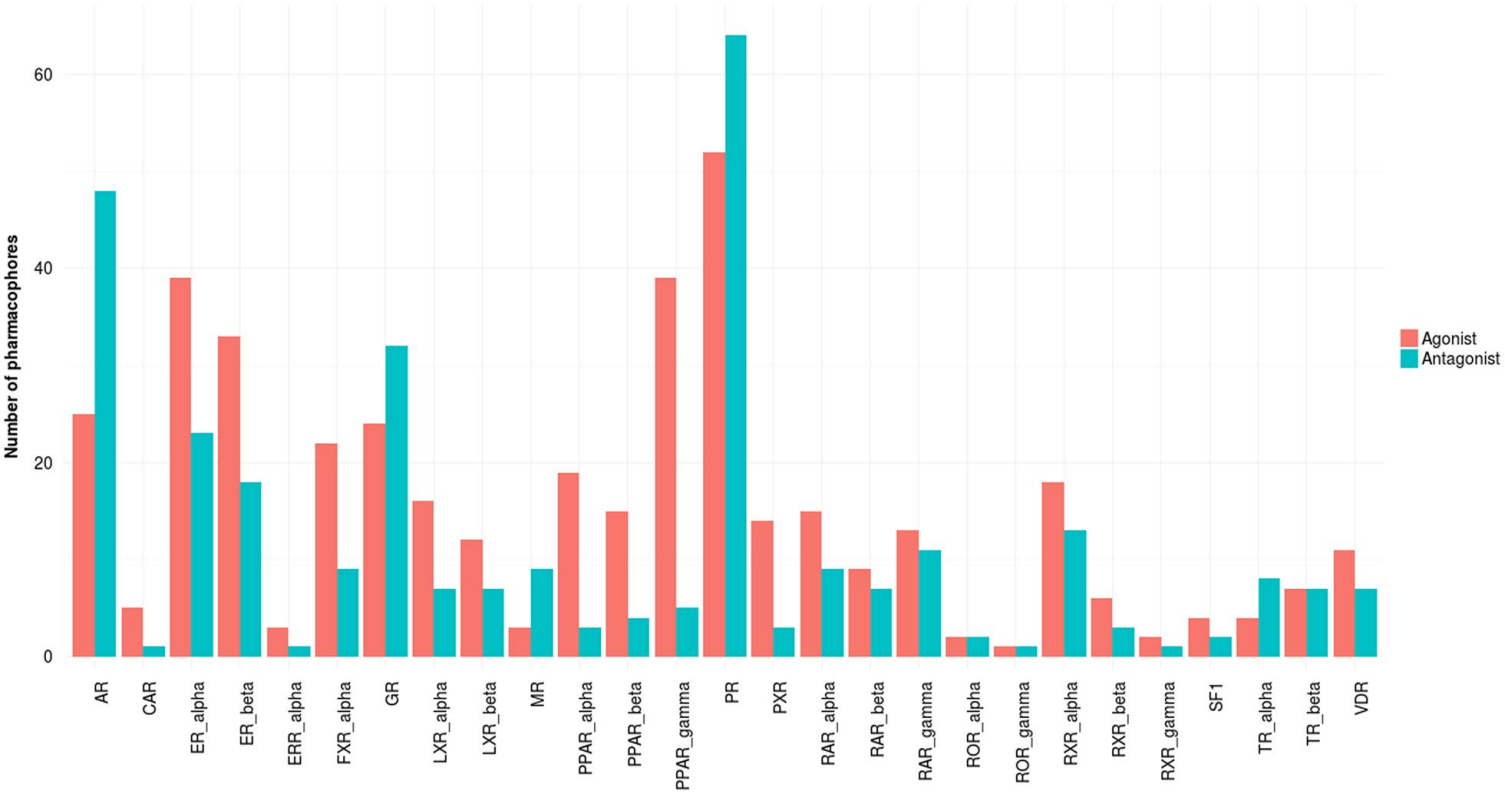
Number of pharmacophores necessary to cover each NRLiSt BDB dataset and included in the “SBLB agonist selective pharmacophores” (*pink*) and “SBLB antagonist selective pharmacophores” (*cyan*) combinations

**Fig. 6:**
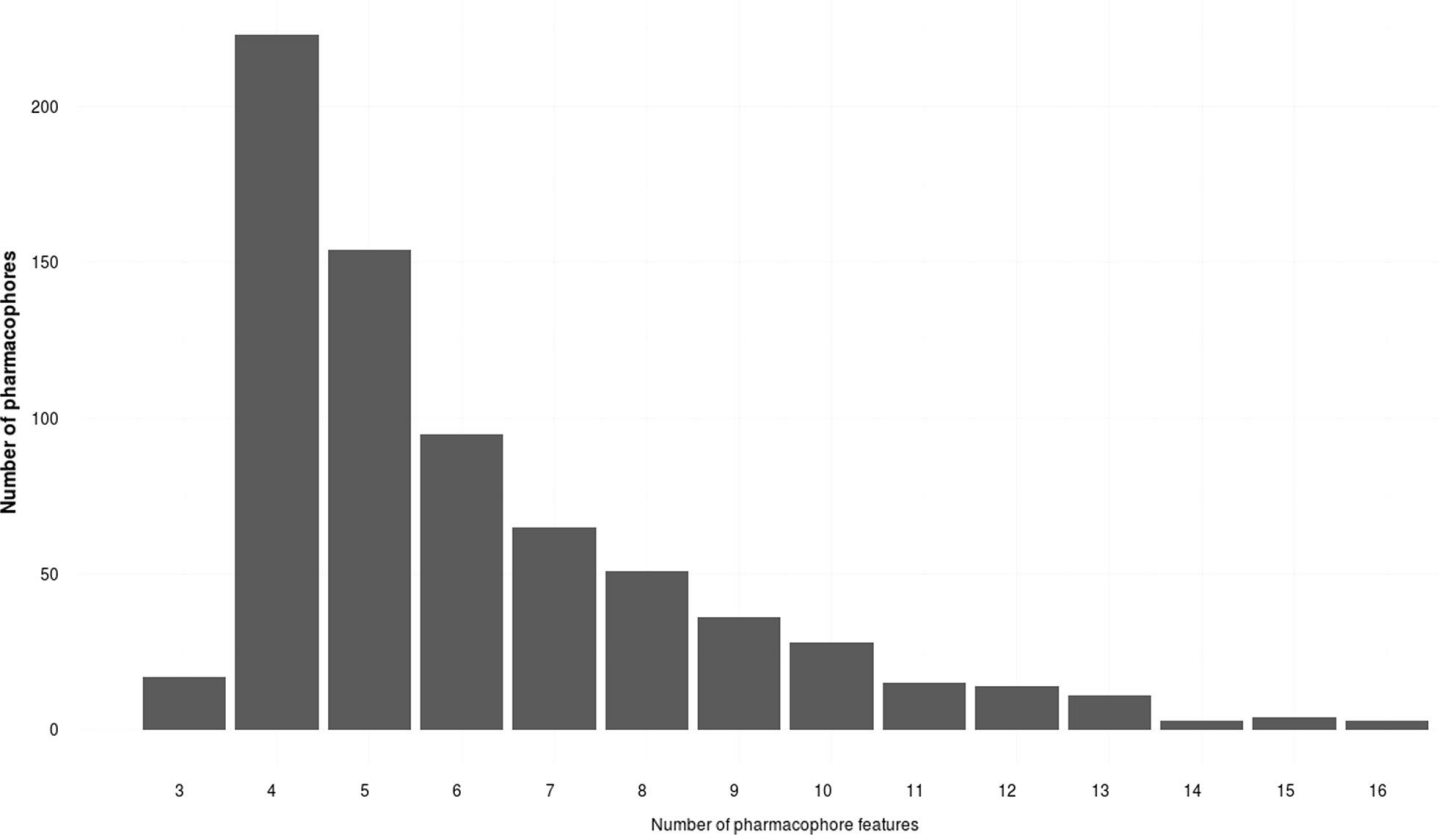
Distribution of the 718 pharmacophores generated for this study according to their number of pharmacophore features (without the number of exclusion volume spheres)

All the graphs were produced with the statistical and graphical tool R (http://www.r-project.org/). The ggplot2 package was used to produce the barplot of Figs. 2, 3, 4, 5 and 6. The corrplot and RColorBrewer packages were used to produce the graph of pharmacophores selectivity using the recall (R) value (Fig. 9). For each dataset, the recall (R), the specificity (Sp) and the Matthew’s correlation coefficient (MCC) were computed as follows:

$$R = \frac{TP}{TP + FN};\quad Sp = \frac{TN}{TN + FP};\quad MCC = \frac{TP \times TN - FP \times FN}{{\sqrt {\left( {TP + FN} \right)\left( {TN + FP} \right)\left( {TP + FP} \right)\left( {TN + FN} \right)} }}$$

with TP the number of true positives (number of active compounds of the dataset retrieved as screening hits), FN the number of false negatives (number of active compounds of the dataset not retrieved as screening hits), TN the number of true negatives (number of inactive compounds of the dataset not retrieved as screening hits), FP (number of inactive compounds of the dataset retrieved as screening hits). As we chose to generate only selective agonist or antagonist pharmacophores, the number of FP was always equal to 0 and thus the Sp value was always equal to 1. Similarly, in the SB approach, when the number of TP was equal to 0, it was not possible to compute the MCC value (because the denominator value is equal to 0), and the MCC value was qualified as not determined (ND).

## Results

### Structure-based pharmacophore modeling

338 3D SB pharmacophores were generated from the 339 PDB structures included in the NRLiSt BDB. The protein structures included in the NRLiSt BDB are classified according to the pharmacological profile of the ligand bound in the active site: 266 agonist-bound structures, 17 antagonist-bound structures, 55 other-bound structures (partial agonists, modulators, inverse agonists etc.). Since only 1 apo structure was available for RXR_gamma, no 3D SB pharmacophore could be generated and this NR was excluded for this part of the study. For respectively 25 and 10 out the 26 remaining NRs, at least one agonist-bound or one antagonist-bound structure was available. Using the screening protocol described in the “Methods” section, we succeeded in generated at least one pharmacophore that was selective for agonist ligands for 25 NRs out of the 26 used, and at least one pharmacophore selective for antagonist ligands for 9 NRs out of 26. As presented in the “Methods” section, all these pharmacophores were gathered into two groups: “SB agonist selective pharmacophores” and “SB antagonist selective pharmacophores”, and redundant pharmacophores were removed. The average recall for the “SB agonist selective pharmacophores” was of 55 %, ranging from 0 % for SF1 to 98 % for PPAR_gamma whereas the average recall for “SB antagonist selective pharmacophores” was of 8 %, ranging from 0 % for AR, CAR, ERR_alpha, FXR_alpha, LXR_alpha, LXR_beta PPAR_alpha, PPAR_beta, PPAR_gamma, PXR, RAR_beta, RAR_gamma, RXR_beta, SF1, TR_alpha, TR_beta and VDR to 61 % for RAR_alpha (Fig. 2; Table 1). The average MCC value for the “SB agonist selective pharmacophores” was of 0.484, ranging from 0.088 for CAR (the SF1 MCC value was ND) to 0.881 for RXR_alpha whereas the average MCC value for “SB antagonist selective pharmacophores” was of 0.326, ranging from 0.097 for RXR_alpha (the MCC value was ND for AR, CAR, ERR_alpha, FXR_alpha, LXR_alpha, LXR_beta PPAR_alpha, PPAR_beta, PPAR_gamma, PXR, RAR_beta, RAR_gamma, RXR_beta, SF1, TR_alpha, TR_beta and VDR) to 0.712 for RAR_alpha (Table 1).

**Table 1:** Recalls (R), specificity (Sp) and MCC values obtained using the SB approach, the LB approach and the combination of SB and LB approaches (SBLB) for each NRLiSt BDB dataset

	SB approach			LB approach			SBLB approach		
	R	Sp	MCC	R	Sp	MCC	R	Sp	MCC
AR_agonist_ligands	0.683	1.000	0.738	0.889	1.000	0.903	1.000	1.000	1.000
AR_antagonist_ligands	0.000	1.000	ND	1.000	1.000	1.000	1.000	1.000	1.000
CAR_agonist_ligands	0.121	1.000	0.088	1.000	1.000	1.000	1.000	1.000	1.000
CAR_antagonist_ligands	0.000	1.000	ND	1.000	1.000	1.000	1.000	1.000	1.000
ER_alpha_agonist_ligands	0.695	1.000	0.595	0.972	1.000	0.945	1.000	1.000	1.000
ER_alpha_antagonist_ligands	0.412	1.000	0.589	0.890	1.000	0.927	0.993	1.000	0.995
ER_beta_agonist_ligands	0.663	1.000	0.478	0.919	1.000	0.795	1.000	1.000	1.000
ER_beta_antagonist_ligands	0.074	1.000	0.251	0.868	1.000	0.921	1.000	1.000	1.000
ERR_alpha_agonist_ligands	0.308	1.000	0.277	1.000	1.000	1.000	1.000	1.000	1.000
ERR_alpha_antagonist_ligands	0.000	1.000	ND	1.000	1.000	1.000	1.000	1.000	1.000
FXR_alpha_agonist_ligands	0.584	1.000	0.319	0.984	1.000	0.914	1.000	1.000	1.000
FXR_alpha_antagonist_ligands	0.000	1.000	ND	1.000	1.000	1.000	1.000	1.000	1.000
GR_agonist_ligands	0.317	1.000	0.453	0.986	1.000	0.988	1.000	1.000	1.000
GR_antagonist_ligands	0.112	1.000	0.230	0.995	1.000	0.994	0.995	1.000	0.994
LXR_alpha_agonist_ligands	0.425	1.000	0.324	0.996	1.000	0.988	0.996	1.000	0.988
LXR_alpha_antagonist_ligands	0.000	1.000	ND	1.000	1.000	1.000	1.000	1.000	1.000
LXR_beta_agonist_ligands	0.682	1.000	0.406	0.992	1.000	0.972	0.995	1.000	0.986
LXR_beta_antagonist_ligands	0.000	1.000	ND	1.000	1.000	1.000	1.000	1.000	1.000
MR_agonist_ligands	0.556	1.000	0.735	0.889	1.000	0.940	1.000	1.000	1.000
MR_antagonist_ligands	0.152	1.000	0.102	1.000	1.000	1.000	1.000	1.000	1.000
PPAR_alpha_agonist_ligands	0.851	1.000	0.166	0.999	1.000	0.935	1.000	1.000	1.000
PPAR_alpha_antagonist_ligands	0.000	1.000	ND	1.000	1.000	1.000	1.000	1.000	1.000
PPAR_beta_agonist_ligands	0.634	1.000	0.137	1.000	1.000	1.000	1.000	1.000	1.000
PPAR_beta_antagonist_ligands	0.000	1.000	ND	1.000	1.000	1.000	1.000	1.000	1.000
PPAR_gamma_agonist_ligands	0.982	1.000	0.464	0.933	1.000	0.253	1.000	1.000	1.000
PPAR_gamma_antagonist_ligands	0.000	1.000	ND	1.000	1.000	1.000	1.000	1.000	1.000
PR_agonist_ligands	0.078	1.000	0.231	0.951	1.000	0.958	0.966	1.000	0.969
PR_antagonist_ligands	0.179	1.000	0.261	0.994	1.000	0.992	0.998	1.000	0.997
PXR_agonist_ligands	0.720	1.000	0.379	0.960	1.000	0.782	1.000	1.000	1.000
PXR_antagonist_ligands	0.000	1.000	ND	1.000	1.000	1.000	1.000	1.000	1.000
RAR_alpha_agonist_ligands	0.508	1.000	0.506	0.970	1.000	0.956	0.977	1.000	0.967
RAR_alpha_antagonist_ligands	0.606	1.000	0.712	1.000	1.000	1.000	1.000	1.000	1.000
RAR_beta_agonist_ligands	0.277	1.000	0.262	1.000	1.000	1.000	1.000	1.000	1.000
RAR_beta_antagonist_ligands	0.000	1.000	ND	1.000	1.000	1.000	1.000	1.000	1.000
RAR_gamma_agonist_ligands	0.705	1.000	0.647	1.000	1.000	1.000	0.992	1.000	0.988
RAR_gamma_antagonist_ligands	0.000	1.000	ND	1.000	1.000	1.000	1.000	1.000	1.000
ROR_alpha_agonist_ligands	0.333	1.000	0.537	1.000	1.000	1.000	1.000	1.000	1.000
ROR_alpha_antagonist_ligands	0.308	1.000	0.277	1.000	1.000	1.000	1.000	1.000	1.000
ROR_gamma_agonist_ligands	0.571	1.000	0.571	1.000	1.000	1.000	1.000	1.000	1.000
ROR_gamma_antagonist_ligands	0.250	1.000	0.418	1.000	1.000	1.000	1.000	1.000	1.000
RXR_alpha_agonist_ligands	0.900	1.000	0.881	0.948	1.000	0.935	1.000	1.000	1.000
RXR_alpha_antagonist_ligands	0.015	1.000	0.097	0.985	1.000	0.988	1.000	1.000	1.000
RXR_beta_agonist_ligands	0.923	1.000	0.734	0.985	1.000	0.928	1.000	1.000	1.000
RXR_beta_antagonist_ligands	0.000	1.000	ND	1.000	1.000	1.000	1.000	1.000	1.000
RXR_gamma_agonist_ligands	0.000	1.000	ND	1.000	1.000	1.000	1.000	1.000	1.000
RXR_gamma_antagonist_ligands	0.000	1.000	ND	1.000	1.000	1.000	1.000	1.000	1.000
SF1_agonist_ligands	0.000	1.000	ND	1.000	1.000	1.000	1.000	1.000	1.000
SF1_antagonist_ligands	0.000	1.000	ND	1.000	1.000	1.000	1.000	1.000	1.000
TR_alpha_agonist_ligands	0.913	1.000	0.821	0.884	1.000	0.775	1.000	1.000	1.000
TR_alpha_antagonist_ligands	0.000	1.000	ND	1.000	1.000	1.000	1.000	1.000	1.000
TR_beta_agonist_ligands	0.935	1.000	0.837	0.948	1.000	0.865	1.000	1.000	1.000
TR_beta_antagonist_ligands	0.000	1.000	ND	1.000	1.000	1.000	1.000	1.000	1.000
VDR_agonist_ligands	0.562	1.000	0.508	0.992	1.000	0.985	1.000	1.000	1.000
VDR_antagonist_ligands	0.000	1.000	ND	1.000	1.000	1.000	1.000	1.000	1.000

When the number of true positives was equal to 0, the MCC value was qualified as not determined (ND)

### Ligand-based pharmacophore modeling

#### Ligands clustering

To perform the LB pharmacophore modeling approach, the ligands of each NRLiSt BDB dataset were clustered using the Pharmacophore RDF-Code similarity. The cluster distance was set to 0.4 for the majority of the datasets but was lowered to 0.3 for 15 datasets (AR_agonist, ERR_alpha_agonist, GR_agonist, LXR_alpha_agonist, LXR_beta_agonist, PR_agonist, RXR_alpha_agonist, RXR_beta_agonist, RXR_beta_antagonist, RXR_gamma_agonist, TR_alpha_agonist, TR_alpha_antagonist, TR_beta_agonist, TR_beta_antagonist, VDR_antagonist) and to 0.2 for 1 dataset (RXR_alpha_antago). From 1 cluster (for the ERR_alpha_agonist, ROR_gamma_antagonist, and RXR_gamma_antagonist datasets) to 65 clusters (for the ER_alpha_agonist dataset) were generated, with an average of 18 clusters per dataset and a mean value of 7.8 ligands per cluster.

#### 3D ligand-based pharmacophores

Using the screening protocol described in the “Methods” section, we succeeded in generated pharmacophores that were selective for agonist ligands and pharmacophores selective for antagonist ligands for each of the 27 NRs of the NRLiSt BDB. All these pharmacophores were gathered into two groups according to their selectivity for agonist or antagonist ligands, “LB agonist selective pharmacophores” and “LB antagonist selective pharmacophores”. Redundant pharmacophores were eliminated. The “LB agonist selective pharmacophores” were associated with an average recall of 97 % and a mean value of 0.918. The lower recall and MCC value were respectively of 88 % for TR_alpha and 0.253 for PPAR_gamma; the higher recall and MCC values respectively reached 100 % and 1 for CAR, ERR_alpha, PPAR_beta, RAR_beta, ROR_alpha, ROR_gamma, RXR_gamma and SF1. The “LB antagonist selective pharmacophores” presented an average recall of 99 % and a mean MCC value of 0.99, and the individual recall and MCC values were equal to 100 % and 1 for all antagonist datasets but 5 (ER_alpha, ER_beta, GR, PR, RXR_alpha) (Fig. 3; Table 1).

### Combination of structure-based and ligand-based pharmacophores

#### 3D SBLB pharmacophores performance

The “SB agonist selective pharmacophores” and “LB agonist selective pharmacophores” on the one hand and the “SB antagonist selective pharmacophores” and “LB antagonist selective pharmacophores” on the other hand were respectively concatenated into two groups: “SBLB agonist selective pharmacophores” and “SBLB antagonist selective pharmacophores”. Using these combinations of SB and LB pharmacophores, average recalls of 99.7 and 99.9 % and mean MCC values of 0.993 and 0.999 were obtained for agonist and antagonist datasets respectively. The “SBLB agonist selective pharmacophores” were able to retrieve all agonist ligands and no antagonist ligands (i.e. recall of 100 % and MCC values of 1) for all NRs but 5 (LXR_alpha, LXR_beta, PR, RAR_alpha and RAR_gamma). Similarly, the “SBLB antagonist selective pharmacophores” were able to retrieve all antagonist ligands and no agonist ligands (i.e. recall of 100 % and MCC values of 1) for all NRs but 3 (ER_alpha, GR, PR) (Fig. 4; Table 1).

#### Pharmacophores composition

**Fig. 7:**
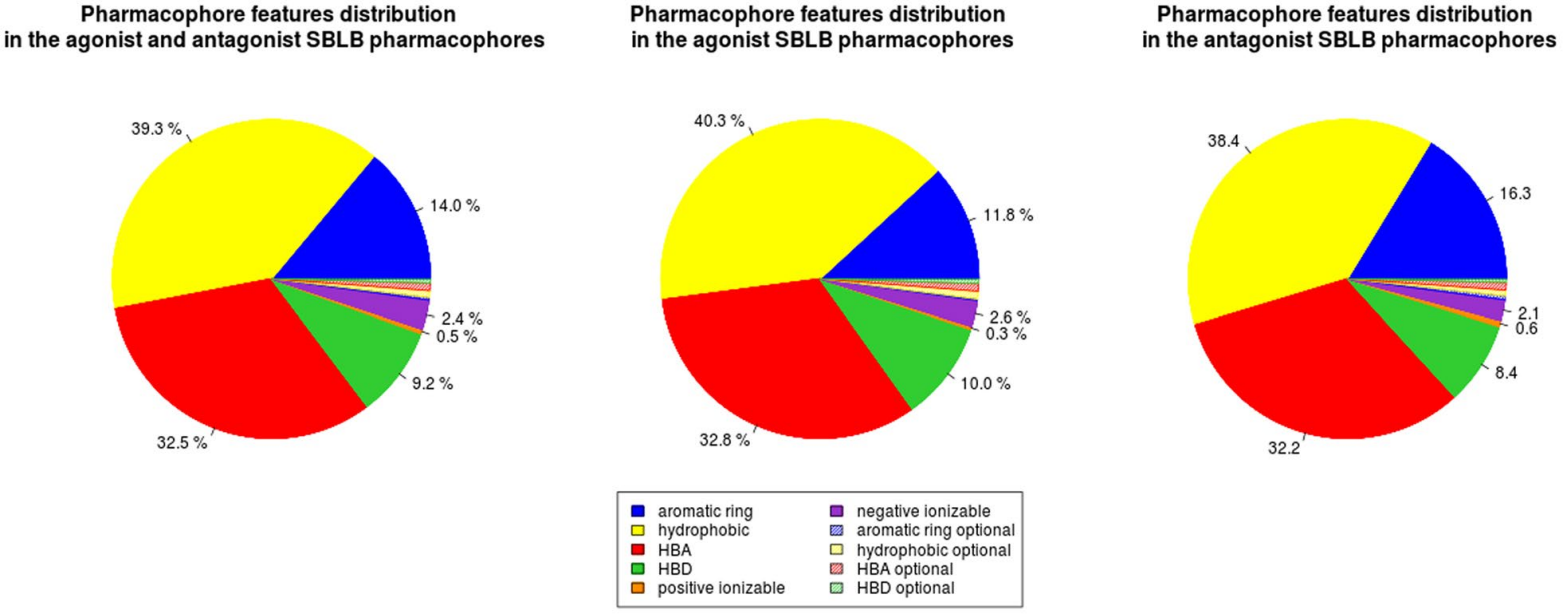
Pie chart representation of the distribution of each type of pharmacophore feature in the total composition of the 718 SBLB agonist and antagonist selective pharmacophores (*left*), of the “SBLB agonist selective pharmacophores” (*middle*) and of the “SBLB antagonist selective pharmacophores” (*right*) selected for the study

**Fig. 8:**
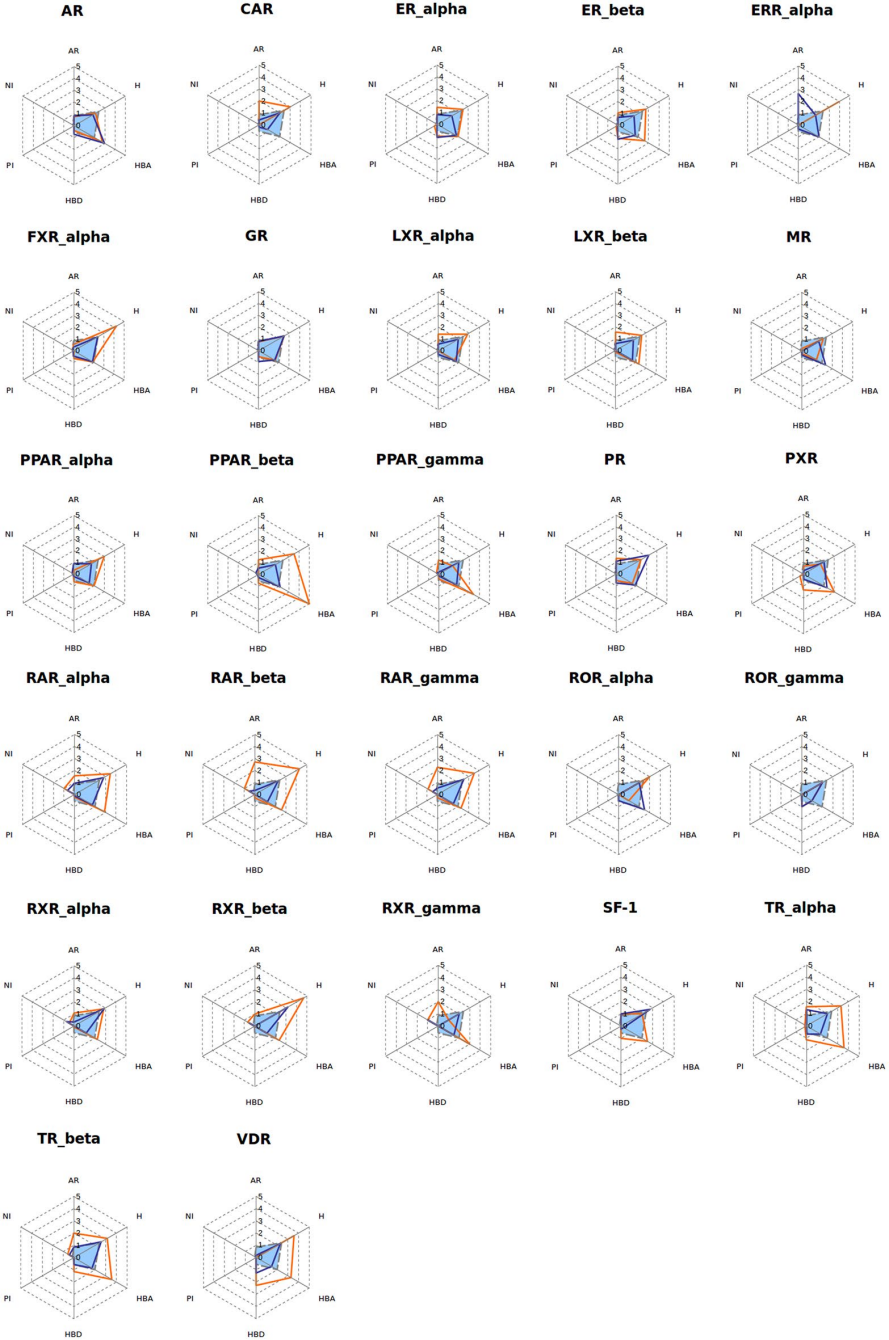
Radiochart representation of the mean values of pharmacophore features composition for the SBLB agonist selective pharmacophores (*blue line*) and the SBLB antagonist selective pharmacophores (*orange line*) compared to the mean value of all SBLB agonist and antagonist selective pharmacophores (*grey dashed line*) for each of the 27 NRs of the NRLiSt BDB [aromatic ring (AR), hydrophobic (H), hydrogen bond acceptor (HBA), hydrogen bond donor (HBD), positive ionizable (PI), negative ionizable (NI)]

 The “SBLB agonist selective pharmacophores” group contained 413 pharmacophores (from 1 pharmacophore for ROR_gamma to 52 pharmacophores for PR) whereas the “SBLB antagonist selective pharmacophores” group contained 305 pharmacophores (from 1 pharmacophore for CAR, ERR_alpha, ROR_gamma and RXR_gamma to 64 pharmacophores for PR) (Fig. 5). The number of pharmacophores that were necessary to cover a given dataset is significantly correlated with the number of ligands in the dataset (Kendall’s tau coefficient, *p* value = 9.55e−15, Additional file 1: Figure S1). These pharmacophores were composed of 3–16 features, with a median value of 5 features per pharmacophore (Fig. 6; Additional file 1: Figure S2A−N, Additional file 1: Tables S1−S54). Pharmacophore features were mainly hydrophobic groups and hydrogen bond acceptors (39.3 and 32.5 % of the total of all pharmacophores features of the 718 SBLB pharmacophores), but aromatic rings and hydrogen bond donors represented also an important part of the pharmacophore features (14.0 and 9.2 % respectively) far ahead negative and positive ionisable area (Fig. 7). These proportions were similar when agonist and antagonist data sets were considered separately (Fig. 7). However, when comparing the SBLB agonist and antagonist pharmacophores for each NR (Fig. 8), some significant differences (p-value <0.05) appeared in the pharmacophore features distribution (Additional file 1: Figure S3A−C). Thus, for respectively 9, 5, 4, 2 and 1 NRs, the SBLB agonist selective pharmacophores included significantly less HBA, hydrophobic, AR, PI and NI features than the corresponding SBLB antagonist selective pharmacophores. Similarly, for 3NRs, the SBLB antagonist selective pharmacophores included significantly less HBD features than the SBLB agonist selective pharmacophores. Each pharmacophore allowed to retrieve from 1 to 1299 ligands, with an average value of 32 ligands retrieved per pharmacophore (Additional file 1: Figure S2A−N, Additional file 1: Tables S1−S54).

#### Pharmacophores selectivity

**Fig. 9:**
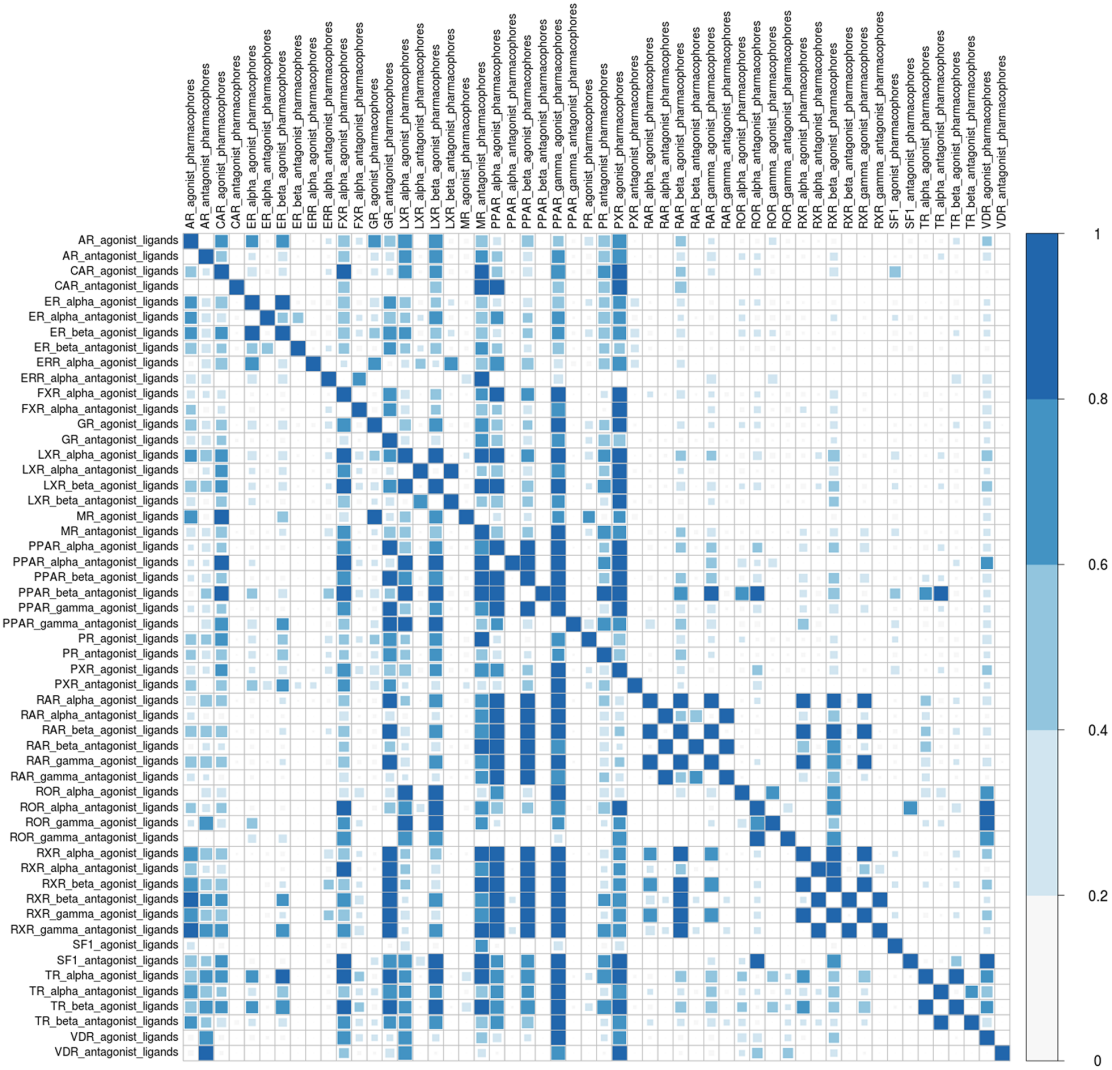
Corrplot representing the recalls obtained for each “SBLB agonist selective pharmacophores” and “SBLB antagonist selective pharmacophores” against the NRLiSt BDB

To evaluate the pharmacophores selectivity for their dedicated NR ligands, each “SBLB agonist selective pharmacophores” and “SBLB antagonist selective pharmacophores” combinations were screened against all the other NRLiSt BDB datasets of ligands. The corresponding recalls are displayed in Fig. 9. The average recall of this large scale cross-screening was of 19.8 %. The “SBLB agonist selective pharmacophores” were associated with higher recalls with an average value of 28.8 versus 10.8 % for the “SBLB antagonist selective pharmacophores”. The most selective combination of pharmacophores was the PPAR_beta “SBLB antagonist selective pharmacophores” with an average recall of 0.001 %, and the less selective pharmacophores were the PPAR_gamma “SBLB agonist selective pharmacophores” with an average recall of 76 %. For 29 combinations of pharmacophores, the average recall was below 10 %. For only 8 combinations of pharmacophores, the average recall was above 50 %. This selectivity was significantly correlated with the number of ligands in the dataset that was used to generate the pharmacophores (Kendall’s tau coefficient, p-value = 3.476e−8, Additional file 1: Figure S4) and with the number of pharmacophores included in the combination for the considered dataset (Kendall’s tau coefficient, p-value = 5.915e−5, Additional file 1: Figure S5). The selectivity could also be correlated with the active ligands over decoys ratio (Kendall’s tau coefficient, p-value = 4.461e-11, Additional file 1: Figure S6).

## Discussion

### Structure-based pharmacophore modeling

In the SB pharmacophore modeling approach, pharmacophores are intuitively derived from the analysis of experimentally determined (X-ray or NMR) target-ligand complexes [34]. The identified pharmacophore features represent chemical features directly involved in the ligand-binding site interactions [40].

The PDB structures included in the NRLiSt BDB were used to generate SB pharmacophores. RXR_gamma was excluded of this part of the study due to the absence of a holo PDB structure. For the remaining 26 NRs, we were able to create pharmacophores that were selective for agonist ligands and pharmacophores that were selective for antagonist ligands. However this selectivity was not always achieved since the generated SB pharmacophores only covered a small part of the NRLiSt BDB ligands chemical space. Indeed, the recalls for “SB agonist selective pharmacophores” and “SB antagonist selective pharmacophores” were respectively of 55 and 8 % and the mean MCC values were 0.484 and 0.326, both varied greatly with the datasets. Very poor performance (recall of 0 % for 1 agonist dataset and 17 antagonist datasets), to very good performance (recall superior or equal to 90 % for 5 agonist datasets and MCC value superior to 0.8 for 3 agonist dataset) were obtained by screening the SB pharmacophores against the corresponding NRLiSt BDB datasets.

These performance variations were highly linked to the availability of PDB structures and to the structural diversity of the ligands that were co-cristallized in these structures. In particular, no antagonist-bound structure was available for 15 out of the 17 NRs for which no SB antagonist selective pharmacophore could be generated. For the 2 remaining NRs, PPAR_alpha and PPAR_gamma, one antagonist-bound structure was available but the SB pharmacophores created with these structures were non selective for antagonist ligands. In the same way, two agonist-bound structures were available for SF1 but no selective pharmacophore could be obtained. Surprisingly, for ROR_alpha, no antagonist-bound structure was available, but we were able to obtain an antagonist selective SB pharmacophore. Similarly, the higher recall was associated with the PPAR_gamma agonist dataset, the larger NRLiSt BDB dataset in terms of number of PDB complexes with 84 structures available.

**Fig. 10:**
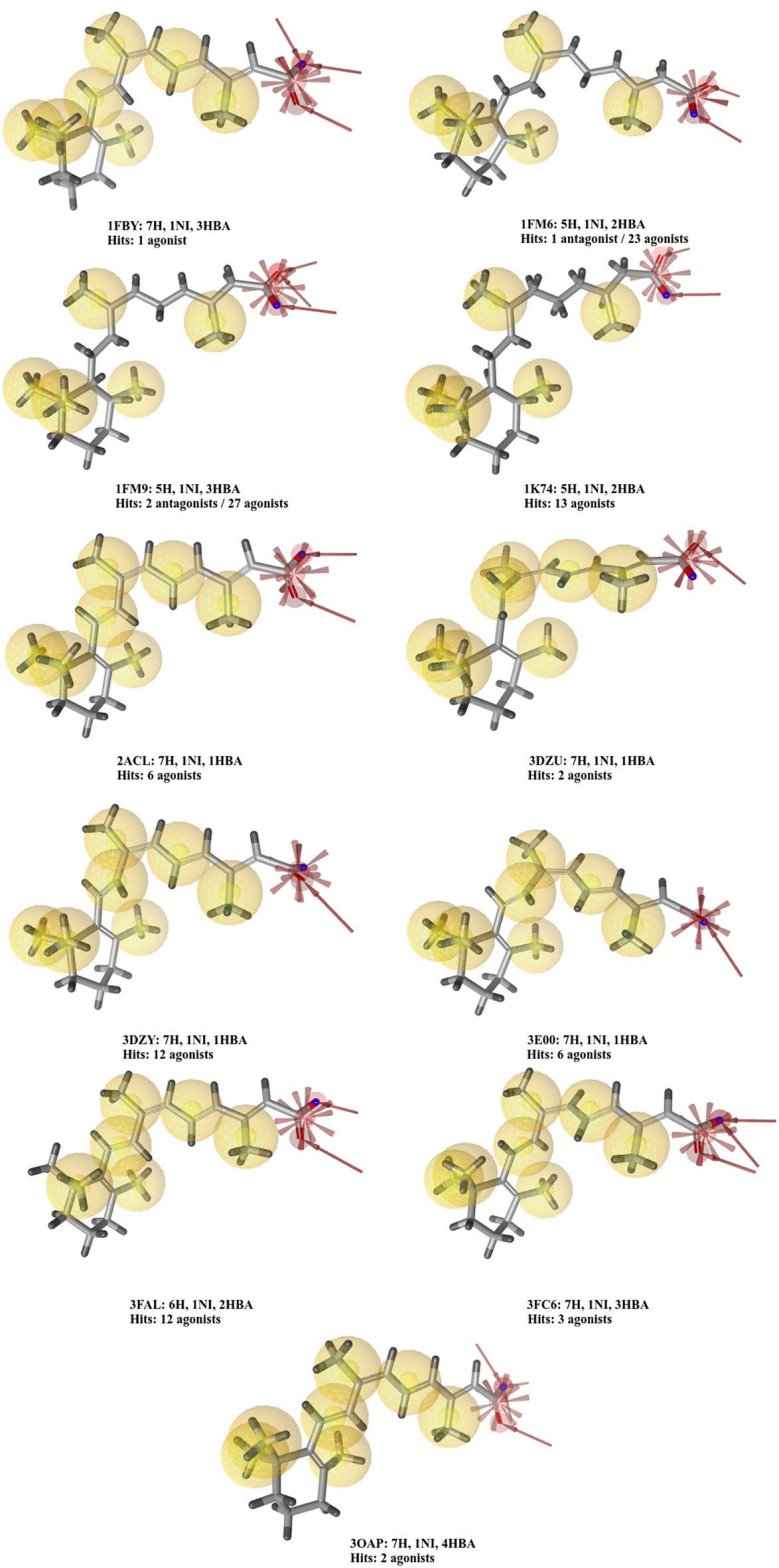
Representation of the structure-based pharmacophores generated with the 11 RXR_alpha PDB structures co-crystallized with 9-*cis*-retinoic acid with their corresponding number of hits identified in virtual screening

Another interesting point was that SB pharmacophores obtained from similar NR–ligand complexes of different PDB structures could be different. For example, 11 RXR_alpha PDB structures co-crystallized with 9-*cis*-retinoic acid were included in the NRLiSt BDB and their corresponding 11 SB pharmacophores differed in the composition and the distribution of pharmacophore features and in the resulting hits (Fig. 10).

#### Ligand-based pharmacophore modeling

The LB pharmacophore modeling identifies the maximum common set of chemical features of an ensemble of ligands supposed to bind to the same active site [34]. The pharmacophore features are presumed to be essential for the ligand-binding site interactions but no structural experimental data of these interactions are used [40].

### Ligands clustering

The NRLiSt BDB datasets of ligands were too large and too structurally dissimilar to be represented by one unique pharmacophore. Thus, we performed a first step of ligand clustering using the LigandScout Ligand-set Clustering tool with default settings except for the cluster distance threshold that was manually adapted for each NRLiSt BDB dataset. The distance, by default set to 0.4, was lowered for some datasets to obtain a homogeneous ligands distribution. The number of resulting clusters varied for each dataset from 1 to 65. Some of the NRLiSt BDB ligands were structurally dissimilar to all the other ligands of their respective dataset and stood alone in their own cluster. Since in the LB approach two or more molecules are necessary to create a pharmacophore, whenever possible, these singletons were attributed to other clusters.

#### 3D ligand-based pharmacophores

For each cluster, a LB pharmacophore was generated and evaluated for its selectivity. Conversely to the outcomes obtained with the SB approach, pharmacophores that were selective for agonist ligands and pharmacophores that were selective for antagonist ligand were obtained for all of the 27 NRs of the NRLiSt BDB. Since the ligand-based approach does not depend on the availability and the diversity of PDB structures, the recalls and the MCC values associated with the 3D LB pharmacophores were superior to those achieved with the 3D SB pharmacophores for all datasets but the PPAR_gamma agonist dataset. Conversely to the SB approach again, using the 3D LB pharmacophores, higher recalls and MCC values were obtained with the antagonist datasets. In particular, a recall of 100 % and a MCC value of 1 were achieved for all antagonist datasets but 5 whereas only 8 agonist datasets were fully covered by the pharmacophores that were generated.

The LB pharmacophores included exclusion volume spheres that were automatically generated around the best alignment of the ligands used for the creation of the pharmacophore. In the SB pharmacophore modeling approach, the exclusion volume spheres actually represent the shape of the active site whereas in the LB approach, the exclusion volume spheres sterically limit the pharmacophore. For some LB pharmacophores, we manually added exclusion volume spheres to remove decoys compounds using the hypothesis that if inactive compounds map all the require features, their inactivity can be due to steric clashes with the binding site [35, 36].

### Combination of structure-based and ligand-based pharmacophores

Virtual ligand screening protocols often combine LB and SB approaches since including all possible information for a target enhance the chance to find hits [7]. Several studies succeeded in identifying active compounds using hierarchical or parallel association of pharmacophore modeling with either LB and SB approaches [28, 41–49] and a few studies associated SB and LB pharmacophores [50–53]. Combinatorial use of multiple pharmacophore models for a target allowed to cover a larger chemical space of actives compared to a single model [38, 54], and can be used to enhance the hit identification success rate [43, 55–57]. In this study, we decided to combine SB and LB approaches by using one VLS method, the LigandScout pharmacophore modeling tool. The SB and LB selective pharmacophores previously generated were gathered in two pharmacophore ensembles: “SBLB agonist selective pharmacophores” and “SBLB antagonist selective pharmacophores”.

#### 3D SBLB pharmacophores performance

The performance obtained by using a combination of SB and LB pharmacophores was largely superior to the one obtained with the SB approach alone and slightly better than the performance obtained with the LB approach alone since the LB pharmacophores were associated with extremely high recalls and MCC values (close to 100 and 1 % respectively). The recalls and MCC values of SBLB selective pharmacophores didn’t reach 100 and 1 % respectively for only 8 datasets out of the 54 that were used for this study. For two of these datasets (the LXR_alpha agonist and LXR_beta agonist datasets), one ligand could only be represented by a pharmacophore formed by two non-independent pharmacophore features (Additional file 1: Figure S7). This pharmacophore could not be used for virtual screening since no defined alignment may be found for a pharmacophore presenting less than three independent features. Thus, the ligand could not be retrieved by any of the LXR_alpha and LXR_beta agonist selective pharmacophores, which explains the resulting recall of 99.6 %. For the 6 remaining datasets, some active ligands could not be separated from decoys because of their high structural similarity (Additional file 1: Figures S8−S13).

#### Pharmacophores composition

To discriminate agonist and antagonist ligands of the NRs and cover the chemical space of the NRLiSt BDB datasets, 718 pharmacophores were necessary, with a mean value of 13 pharmacophores per dataset. The number of pharmacophores per data set was significantly correlated with the number of ligands in the respective datasets (Kendall’s tau coefficient, p-value = 9.55e−15). Since the agonist datasets are in majority larger than the antagonist datasets, the average number of pharmacophores per dataset was higher for agonist datasets compared to antagonist datasets (mean values of 15 and 11 pharmacophores per dataset respectively). Consequently, in a large majority, the number of generated selective SBLB pharmacophores was superior for agonist datasets and 4 out the 5 NRs for which the antagonist SBLB pharmacophores outnumbered the agonist ones presented datasets with more antagonist ligands than agonist ligands.

These pharmacophores were composed of 3–16 features, their majority being composed of 4 or 5 features. This finding is of great interest since it shows that pharmacophores with a limited number of features can be used to discriminate agonist and antagonist ligands of the NRs. However, it is important to note that the count of pharmacophore features did not include the number of exclusion volume spheres. The most represented pharmacophore features were hydrophobic groups and hydrogen bond acceptors, and the less represented pharmacophore features were negative and positive ionizable areas. However, some significant differences between SBLB agonist selective pharmacophores and SBLB antagonist selective pharmacophores could be observed, the main trend being that for 9 NRs, the SBLB agonist selective pharmacophores included less HBA features than the SBLB antagonist selective pharmacophores. Interestingly, some overlap exists between SB and LB pharmacophores. For example, the ER_alpha agonist selective pharmacophores include 5 SB pharmacophores and 34 LB pharmacophores. The 5 SB pharmacophores could be aligned with 5, 19, 20, 23 and 25 LB pharmacophores, with a mean of respectively 90, 78, 64, 89 and 87 % of SB features overlapped by LB features (Additional file 1: Table S55; Additional file 1: Figure S14). Similarly, the 6 SB ER_alpha antagonist selective pharmacophores could be aligned with respectively 10, 10, 10, 12, 13 and 15 out of the 17 LB ER_alpha antagonist selective pharmacophores with a mean of respectively 55, 75, 75, 78, 72, 54 and 77 % of SB features overlapped by LB features (Additional file 1: Table S56). For 7 % of the pharmacophores, one or more pharmacophore features were set as optional, which means the ligands mapping all pharmacophore features and the ligands mapping all pharmacophore features but the optional one were considered as hits.

These pharmacophores are able to retrieve a wide range of ligands, from 1 ligand for the most stringent pharmacophores to 1299 for the most powerful one. We could not identify any significant correlation between the number of pharmacophore features and the number of ligands retrieved by each pharmacophore (Additional file 1: Figure S15). Hence, the pharmacophores presenting a small number of features didn’t necessarily retrieve more ligands than the pharmacophores with a larger number of features.

#### Pharmacophores selectivity

Each SBLB combination of agonist selective pharmacophores and antagonist selective pharmacophores was tested for its selectivity for their dedicated NRs ligands on all NRLiSt BDB datasets. We observed that the pharmacophores generated for this study were selective for the NRs activity for which they have been created, the “SBLB antagonist selective pharmacophores” being more selective than the “SBLB antagonist selective pharmacophores”. Particularly, 6 out of the 8 combinations of pharmacophores for which the average recall of the cross-screening study was above 50 % were “SBLB agonist selective pharmacophores”. In addition, 16 out of the 17 combinations of pharmacophores for which the average recall of the cross-screening study was below 5 %, were “SBLB antagonist selective pharmacophores”. This selectivity is associated to three features: the number of ligands in the dataset used to generate the pharmacophores (Kendall’s tau coefficient, p-value = 3.476e−8), the number of pharmacophores included in the combination for the considered dataset (Kendall’s tau coefficient, p-value = 5.915e−5) and the active ligands over decoys ratio (Kendall’s tau coefficient, p-value = 4.461e−11). Hence, the selectivity of the combination of pharmacophores against its dedicated NRs activity decreased for datasets with a large number of ligands (and consequently with a large number of pharmacophores included in the combination) or with a number of active ligands largely outnumbering the number of decoys. This last point was particularly true for the PPAR_alpha, PPAR_beta and PPAR_gamma agonist datasets that encompassed more than 800 active ligands, and for which not even 10 antagonist decoys were available. A larger number of PPAR antagonist ligands would be necessary to obtain a pharmacophore that would be more selective for their agonist ligands.

Finally, the selectivity of the combination of pharmacophores also depended on the selectivity of the NRs ligands. Indeed, NRs ligands presented cross-reactivity, which means that one ligand can bind to several NRs, and this cross-reactivity is particularly true between NRs isoforms. This cross-reactivity is reflected in the NRLiSt BDB (NRLiSt BDB Supplementary Information [33]) and also in the pharmacophores that were generated for this study. Indeed, the combinations of pharmacophores created for this study displayed a lack of selectivity between the different NRs isoforms (ER_alpha/ER_beta, LXR_alpha/LXR_beta, PPAR_alpha/PPAR_beta/PPAR_gamma, RAR_alpha/RAR_beta/RAR_gamma, ROR_alpha/ROR_gamma, RXR_alpha/RXR_beta/RXR_gamma, TR_alpha/TR_beta), which is clearly visible with the small checkerboard along the central diagonal in the Fig. 9.

## Conclusion

 In the present work, we aimed to create NRs agonist selective pharmacophores and NRs antagonist selective pharmacophores. Our main objective was to evaluate the use of a 3D pharmacophore modeling approach to discriminate agonist and antagonist ligands of the NRs. We generated with the LigandScout software 3D structure-based pharmacophores and 3D ligand-based pharmacophores using respectively the NRs PDB structures and the sets of NRs ligands included in the NRLiSt BDB. We evaluated and compared the performance of these two approaches by focusing on two features: (1) the ability to generate selective pharmacophores for agonist or antagonist ligands, (2) the recalls associated to the combinations of pharmacophores in order to study the coverage the chemical space of the NRLiSt BDB datasets. Using the structure-based approach, we obtained selective pharmacophores for both agonist and antagonist ligands of the NRs, but since the recalls were correlated with the availability and the diversity of PDB structures, no selective pharmacophore could be generated for some datasets. Using the ligand-based approach, we created pharmacophores selective for agonist ligands and pharmacophores selective for antagonist ligands for each NRLiSt BDB datasets that yielded high performances. However, the best performances were obtained by combining the structure-based and the ligand-based approaches. We identified that: (1) the number of pharmacophores necessary to cover each NRLiSt BDB dataset depended on the number of ligands included in the dataset and (2) a limited number of pharmacophore features, mostly 4 or 5, were sufficient to discriminate agonist and antagonist ligands of the NRs. We also study the selectivity of the combination of the pharmacophores obtained for each dataset against all other datasets and we demonstrated that our pharmacophores were selective for their dedicated NRs ligands, and that this selectivity was associated with three features: the number of ligands in the dataset, the number of pharmacophores in the combination and the active ligands over decoys ratio. In conclusion, we have been able to generate 3D agonist and antagonist selective pharmacophores that cover most of the NRLiSt BDB active ligands chemical space. These 3D pharmacophores can be used as a predictor of the pharmacological activity of NRs ligands.

## Additional file

10.1186/s13321-016-0154-2 Correlation between the number of pharmacophores necessary to cover a given dataset and the number of ligands in the dataset. **Figure S2.** **A**–**N** Representation of the pharmacophore features composition of the “SBLB agonist selective pharmacophores” and “SBLB antagonist selective pharmacophores” combinations for each NRLiSt BDB dataset (left graph). The number of ligands found with each pharmacophore and the total number of unique ligands found by combining the pharmacophores are also illustrated (right graph).**Figure S3.** **A**–**C** Comparison of the distribution of pharmacophore features between SBLB agonist selective pharmacophores and SBLB antagonist selective pharmacophores for each NR of the NRLiSt BDB using the Wilcoxon-test. The red line represents the significance threshold (p-value = 0.05). **Figure S4.** Correlation between the selectivity of a combination of pharmacophore towards their dedicated NRs ligands (average recovery rate against all the others NRLiSt BDB datasets) and the number of ligands in the dataset. **Figure S5.** Correlation between the selectivity of a combination of pharmacophore towards their dedicated NRs ligands (average recovery rate against all the others NRLiSt BDB datasets) and the number of pharmacophores included in the combination. **Figure S6.** Correlation between the selectivity of a combination of pharmacophore towards their dedicated NRs ligands (average recovery rate against all the others NRLiSt BDB datasets) and the active ligands over decoys ratio. **Figure S7.** Structure of the LXR_alpha and LXR_beta agonist that could only be represented by a pharmacophore formed of 2 non-independent features. **Figure S8.** Structure of the ER_alpha antagonist ligand and ER_alpha agonist ligand that could not be separated using 3D pharmacophore models. **Figure S9.** Structure of the GR antagonist ligands and GR agonist ligands that could not be separated using 3D pharmacophore models. **Figure S10.** Structure of the PR agonist ligands and PR antagonist ligands that could not be separated using 3D pharmacophore models. **Figure S11.** Structure of the PR antagonist ligand and PR agonist ligand that could not be separated using 3D pharmacophore models. **Figure S12.** Structure of the RAR_alpha agonist ligands and RAR_alpha antagonist ligands that could not be separated using 3D pharmacophore models. **Figure S13.** Structure of the RAR_gamma agonist ligand and RAR_gamma antagonist ligands that could not be separated using 3D pharmacophore models. **Figure S14.** Overlapping features between the SB_pharmacophore5 ER_alpha agonist selective pharmacophore and LB ER_alpha agonist selective pharmacophores (reference point tethers are represented by the orange circle on the alignment, right graph). **Figure S15.** Correlation between the number of pharmacophore features and the number of ligands retrieved by each pharmacophore. **Table S1**–**Table S54.**. For each NR agonist and antagonist datasets. Pharmacophore features composition of the SBLB agonist or antagonist selective pharmacophores (AR: aromatic ring, H:hydrophobic, HBA: Hydrogen Bond Acceptor, HBD: Hydrogen Bond Donor, PI: Positive ionizable area, NI: negative ionisable area, opt.: feature set as optional) and number of active ligands found with each pharmacophore (“Nbr of agonist or antagonist ligands found”), number of unique active ligands found by combining the pharmacophores (“Accumulated nbr of unique agonist or antagonist ligands found”) and number of decoys found (“Nbr of agonist or antagonist ligands found”). **Table S55.** Overlapping features between ER_alpha the SB agonist selective pharmacophores and the LB agonist selective pharmacophores included in the SBLB agonist combination of pharmacophores (Overlap: number of LB pharmacophore features that overlap SB pharmacophore features, RMS: Root Mean Square of matched features pairs, Score: LigandScout alignment score). **Table S56.** Overlapping features between ER_alpha the SB antagonist selective pharmacophores and the LB antagonist selective pharmacophores included in the SBLB antagonist combination of pharmacophores (Overlap: number of LB pharmacophore features that overlap SB pharmacophore features, RMS: Root Mean Square of matched features pairs, Score: LigandScout alignment score).

## Abbreviations

AR: androgen receptor
CAR: constitutive androstane receptor
DUD-E: directory of useful decoys enhanced
ER_alpha: estrogen receptor α
ER_beta: estrogen receptor β
ERR_alpha: estrogen related receptor α
FXR_alpha: farnesoid X receptor α
GR: glucocorticoid receptor
LB: ligand-based
LXR_alpha: liver X receptor α
LXR_beta: liver X receptor β
MCC: Matthew’s correlation coefficient
MR: mineralocorticoid receptor
NRLiSt BDB: nuclear receptors ligands and structures benchmarking database
NR: nuclear receptor
PDB: Protein Data Bank
PPAR_alpha: peroxisome proliferator activated receptor α
PPAR_beta: peroxisome proliferator activated receptor β
PPAR_gamma: peroxisome proliferator activated receptor γ
PR: progesterone receptor
PXR: pregnane X receptor
RAR_alpha: retinoic acid receptor α
RAR_beta: retinoic acid receptor β
RAR_gamma: retinoic acid receptor γ
ROR_alpha: retinoic acid receptor related orphan receptor α
ROR_gamma: retinoic acid receptor related orphan receptor γ
RXR_alpha: retinoid X receptor α
RXR_beta: retinoid X receptor β
RXR_gamma: retinoid X receptor γ
SB: structure-based
SF1: steroidogenic factor 1
TR_alpha: thyroid hormone receptor α
TR_beta: thyroid hormone receptor β
VDR: vitamin D receptor
