# Supplementary material for: Discriminating agonist and antagonist ligands of the nuclear receptors using 3D-pharmacophores
Source: J Cheminform. 2016 Sep 6;8(1):43. doi: 10.1186/s13321-016-0154-2 (PMC5011875; doi:10.1186/s13321-016-0154-2)
Supplement: Supplementary file 1 — 10.1186/s13321-016-0154-2 Correlation between the number of pharmacophores necessary to cover a given dataset and the number of ligands in the dataset. Figure S2. A–N Representation of the pharmacophore features composition of the “SBLB agonist selective pharmacophores” and “SBLB antagonist selective pharmacophores” combinations for each NRLiSt BDB dataset (left graph). The number of ligands found with each pharmacophore and the total number of unique ligands found by combining the pharmacophores are also illustrated (right graph).Figure S3. A–C Comparison of the distribution of pharmacophore features between SBLB agonist selective pharmacophores and SBLB antagonist selective pharmacophores for each NR of the NRLiSt BDB using the Wilcoxon-test. The red line represents the significance threshold (p-value = 0.05). Figure S4. Correlation between the selectivity of a combination of pharmacophore towards their dedicated NRs ligands (average recovery rate against all the others NRLiSt BDB datasets) and the number of ligands in the dataset. Figure S5. Correlation between the selectivity of a combination of pharmacophore towards their dedicated NRs ligands (average recovery rate against all the others NRLiSt BDB datasets) and the number of pharmacophores included in the combination. Figure S6. Correlation between the selectivity of a combination of pharmacophore towards their dedicated NRs ligands (average recovery rate against all the others NRLiSt BDB datasets) and the active ligands over decoys ratio. Figure S7. Structure of the LXR_alpha and LXR_beta agonist that could only be represented by a pharmacophore formed of 2 non-independent features. Figure S8. Structure of the ER_alpha antagonist ligand and ER_alpha agonist ligand that could not be separated using 3D pharmacophore models. Figure S9. Structure of the GR antagonist ligands and GR agonist ligands that could not be separated using 3D pharmacophore models. Figure S10. Structure of the PR agonist ligands and PR an [file 13321_2016_154_MOESM1_ESM.doc]

Discriminating agonist and antagonist ligands of the nuclear receptors using 3D-pharmacophores

Nathalie Lagarde^1^, Solenne Delahaye^1^, Jean-François Zagury^1^, and Matthieu Montes^1*^

^1^Laboratoire Génomique Bioinformatique et Applications, Équipe d’accueil EA 4627, Conservatoire National des Arts et Métiers, 292 rue Saint Martin, 75003 Paris, FRANCE

^*^To whom correspondance should be addressed: Matthieu Montes, PhD, e-mail: matthieu.montes@cnam.fr


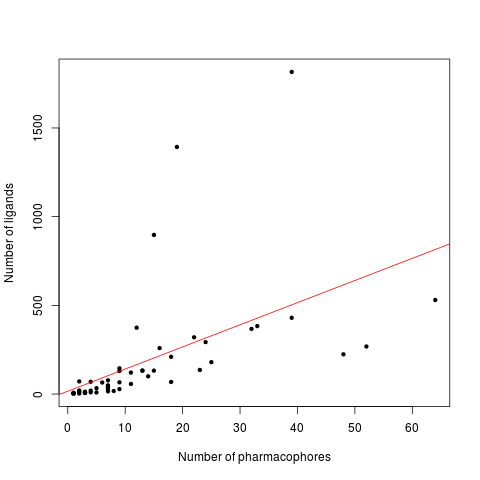


Figure S1. Correlation between the number of pharmacophores necessary to cover a given dataset and the number of ligands in the dataset


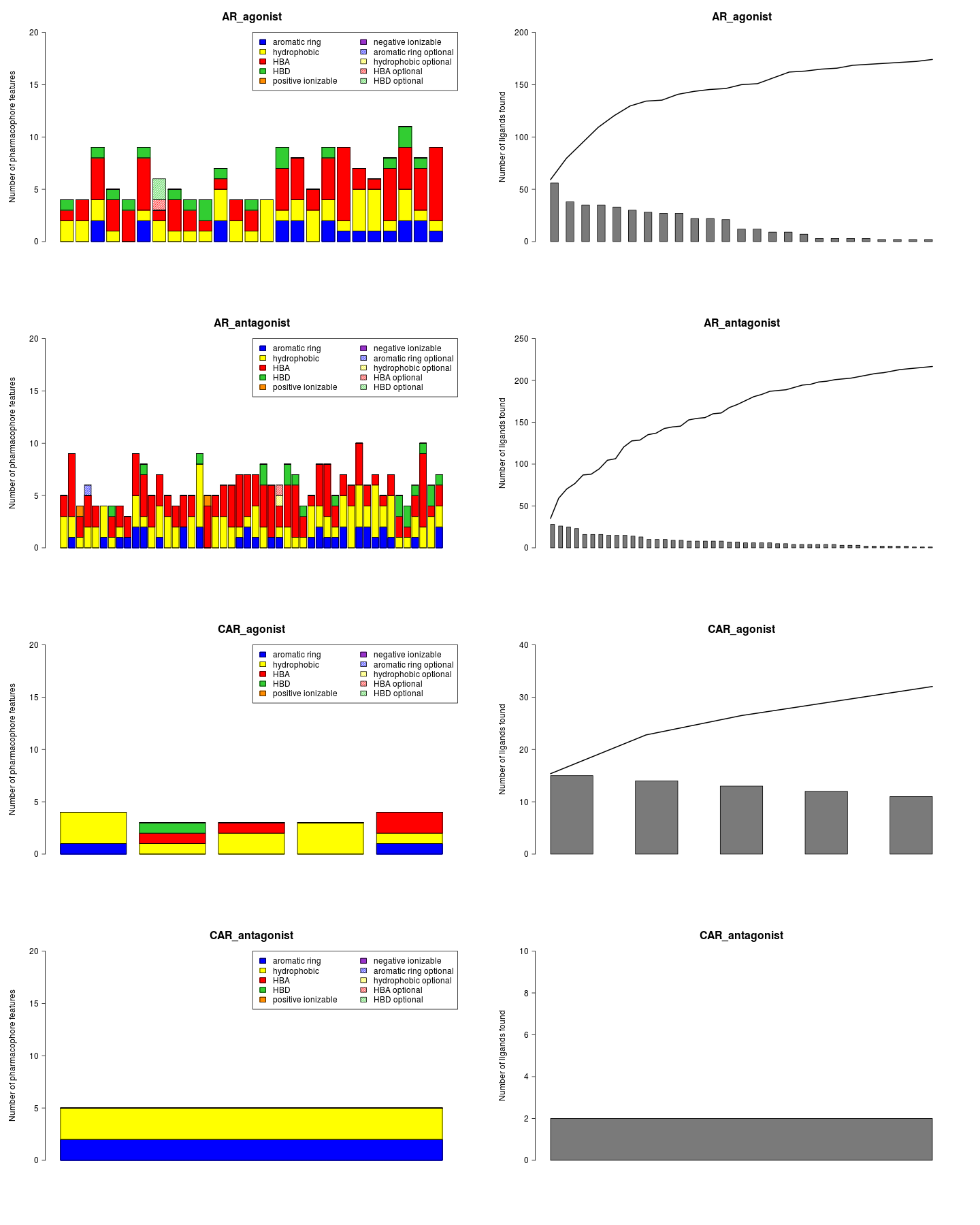


Figure S2A. Representation of the pharmacophore features composition of the “SBLB agonist selective pharmacophores” and “SBLB antagonist selective pharmacophores” combinations for each NRLiSt BDB dataset (left graph). The number of ligands found with each pharmacophore and the total number of unique ligands found by combining the pharmacophores are also illustrated (right graph).


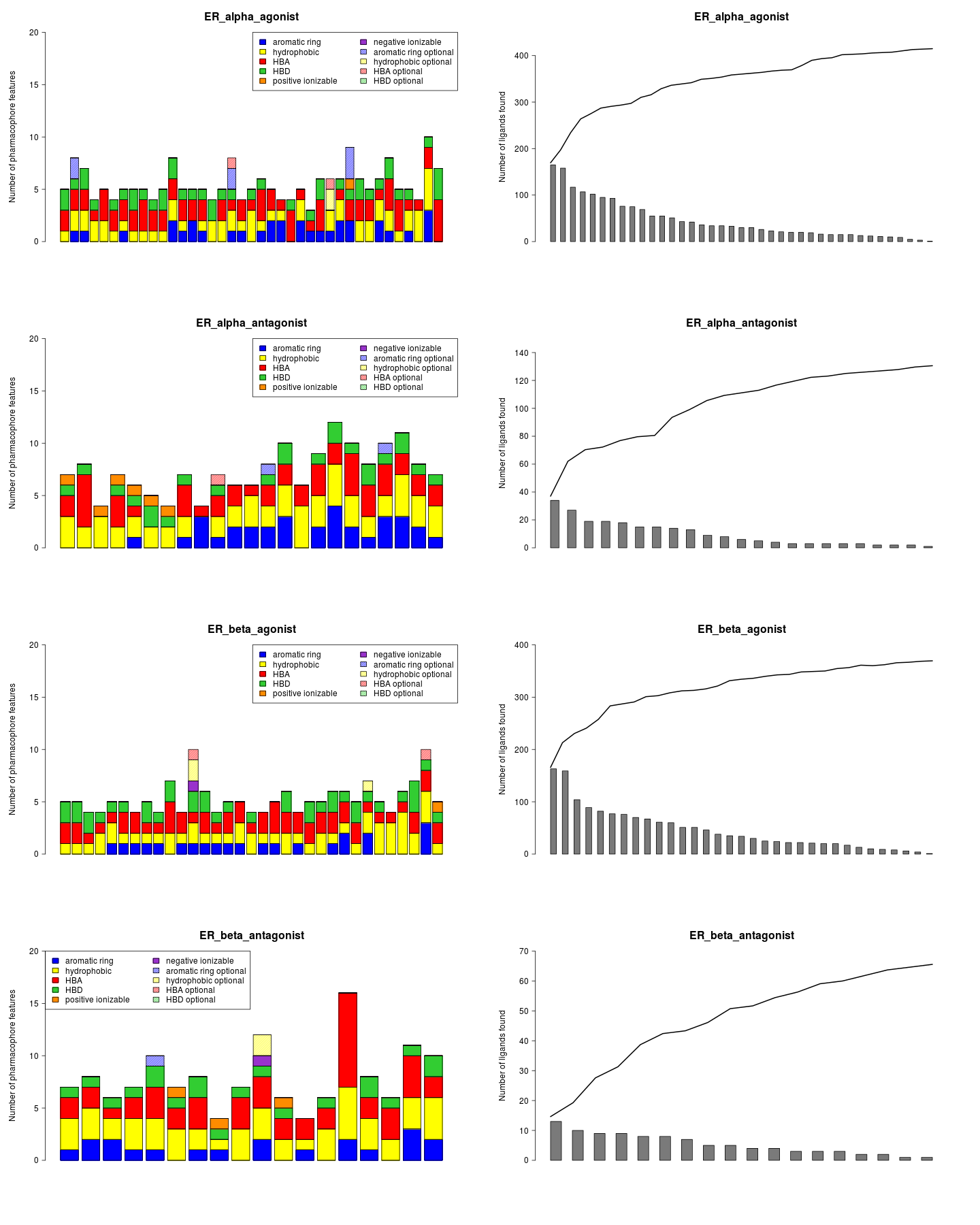


Figure S2B. Representation of the pharmacophore features composition of the “SBLB agonist selective pharmacophores” and “SBLB antagonist selective pharmacophores” combinations for each NRLiSt BDB dataset (left graph). The number of ligands found with each pharmacophore and the total number of unique ligands found by combining the pharmacophores are also illustrated (right graph).


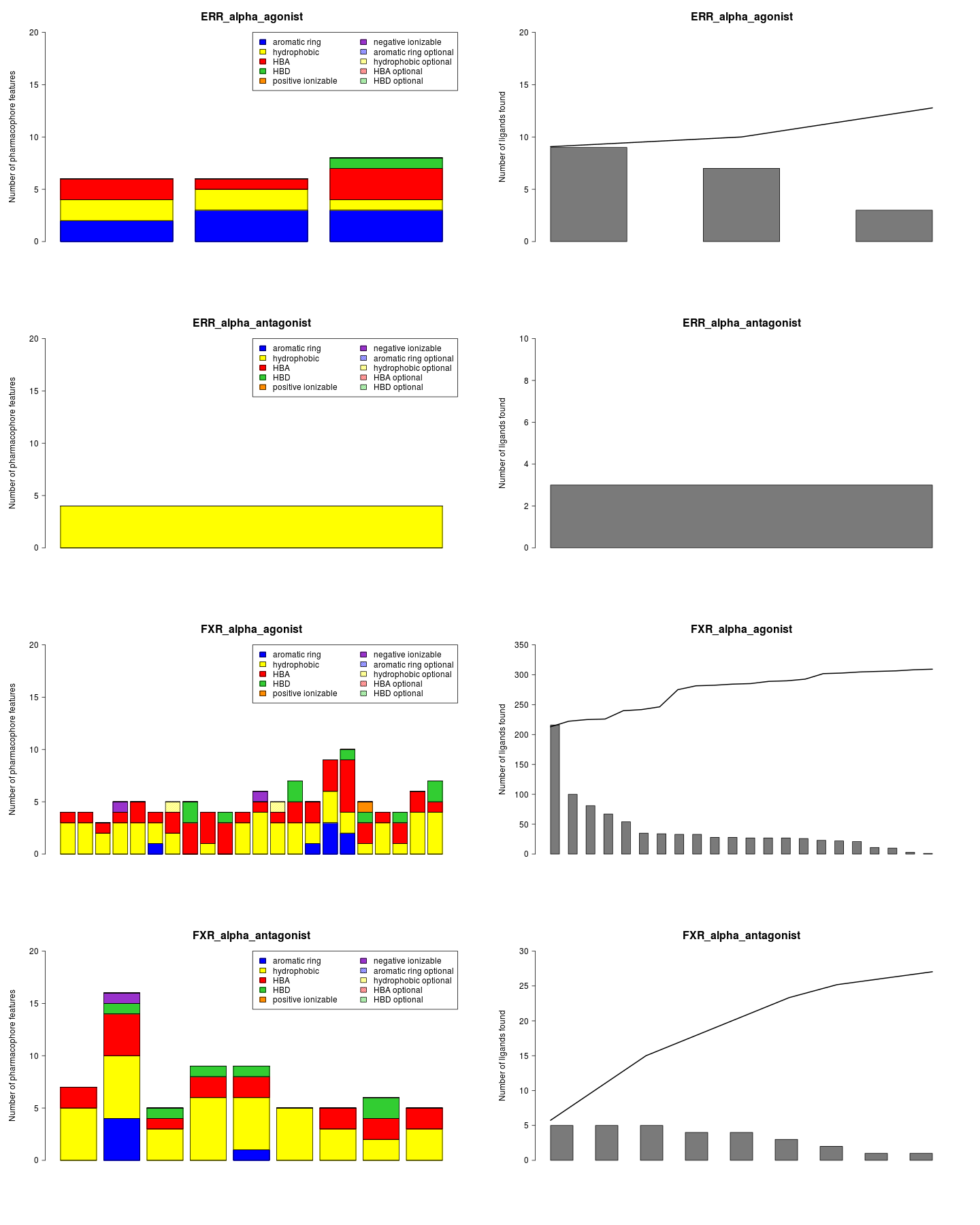


Figure S2C. Representation of the pharmacophore features composition of the “SBLB agonist selective pharmacophores” and “SBLB antagonist selective pharmacophores” combinations for each NRLiSt BDB dataset (left graph). The number of ligands found with each pharmacophore and the total number of unique ligands found by combining the pharmacophores are also illustrated (right graph).


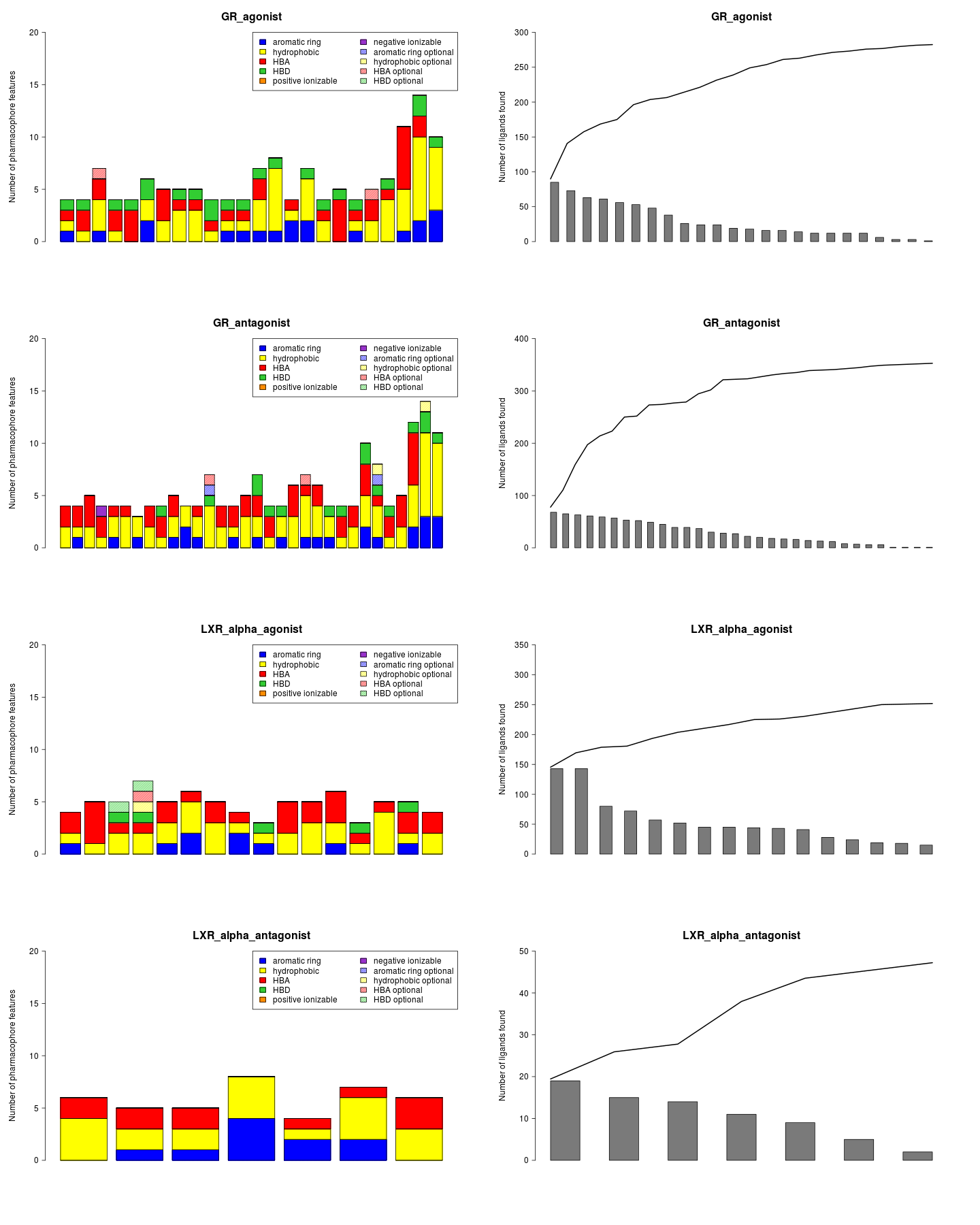


Figure S2D. Representation of the pharmacophore features composition of the “SBLB agonist selective pharmacophores” and “SBLB antagonist selective pharmacophores” combinations for each NRLiSt BDB dataset (left graph). The number of ligands found with each pharmacophore and the total number of unique ligands found by combining the pharmacophores are also illustrated (right graph).


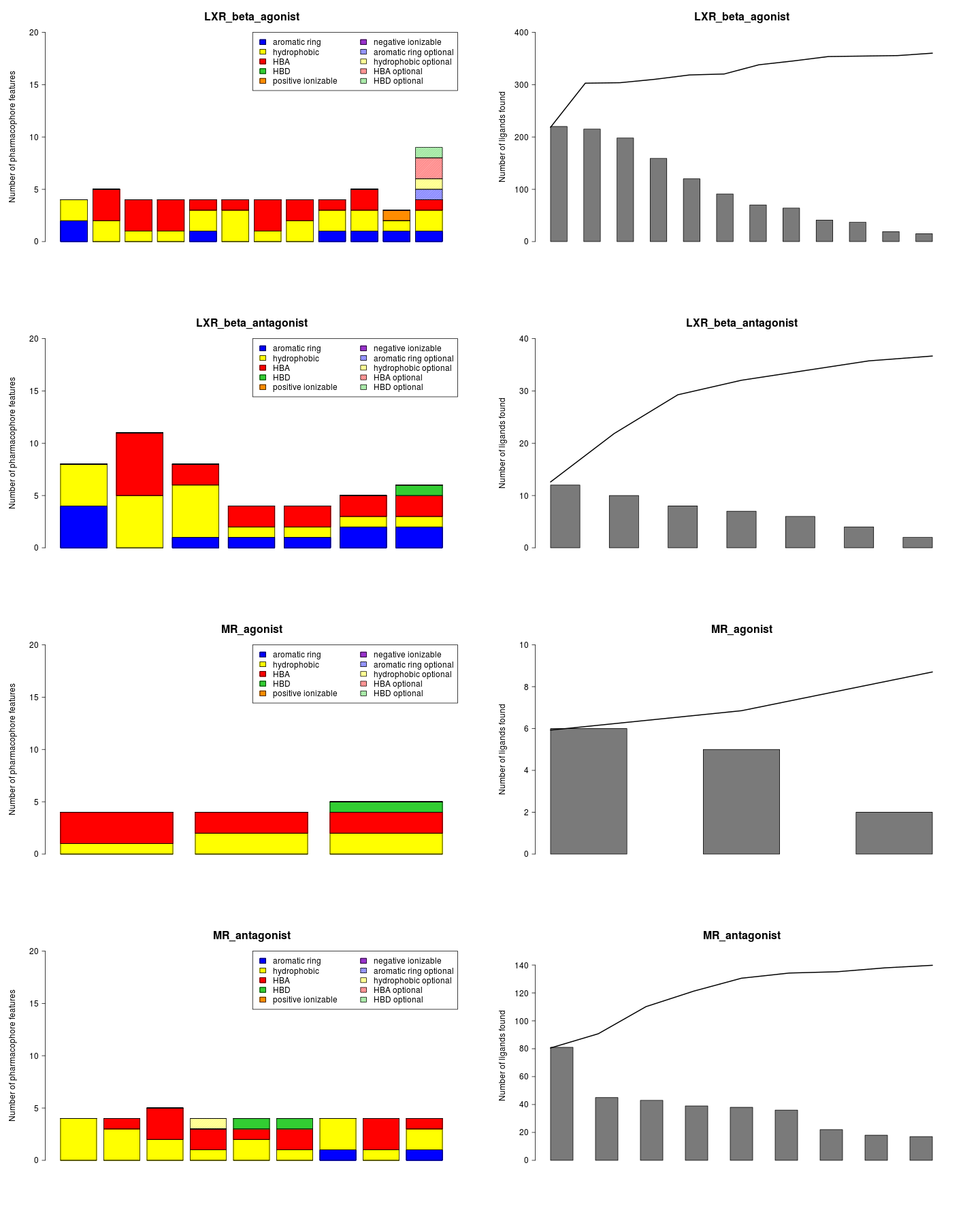


Figure S2E. Representation of the pharmacophore features composition of the “SBLB agonist selective pharmacophores” and “SBLB antagonist selective pharmacophores” combinations for each NRLiSt BDB dataset (left graph). The number of ligands found with each pharmacophore and the total number of unique ligands found by combining the pharmacophores are also illustrated (right graph).


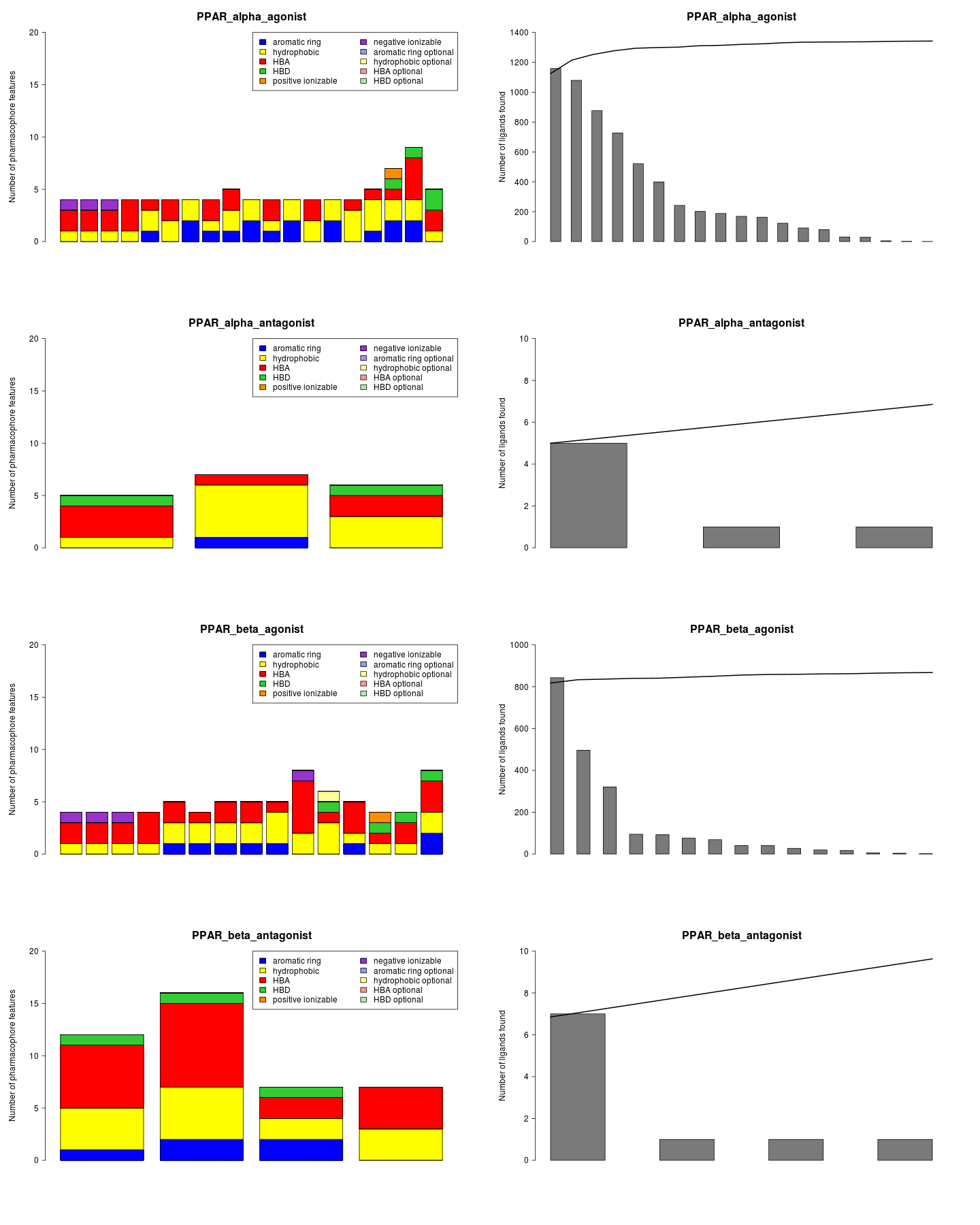


Figure S2F. Representation of the pharmacophore features composition of the “SBLB agonist selective pharmacophores” and “SBLB antagonist selective pharmacophores” combinations for each NRLiSt BDB dataset (left graph). The number of ligands found with each pharmacophore and the total number of unique ligands found by combining the pharmacophores are also illustrated (right graph).


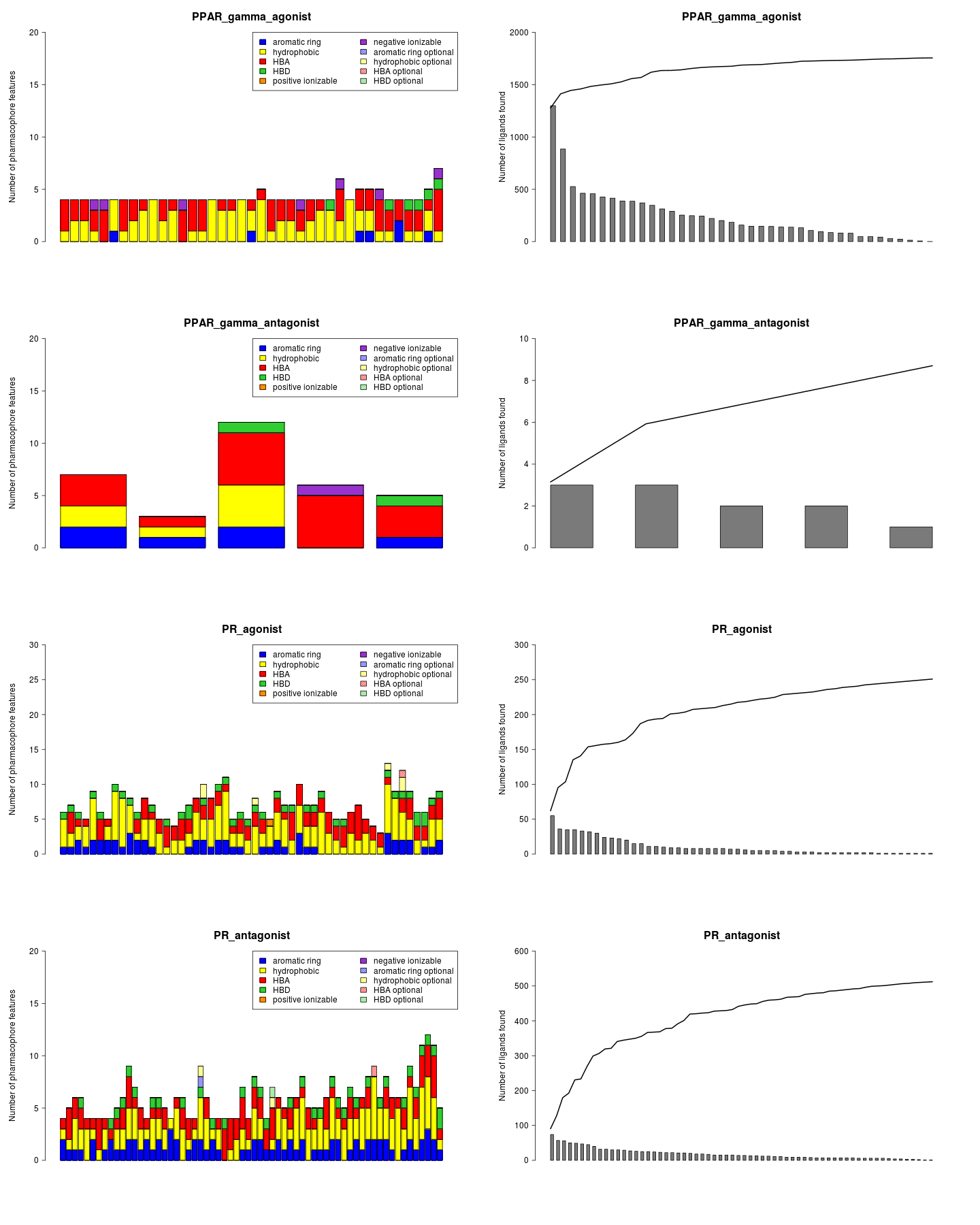


Figure S2G. Representation of the pharmacophore features composition of the “SBLB agonist selective pharmacophores” and “SBLB antagonist selective pharmacophores” combinations for each NRLiSt BDB dataset (left graph). The number of ligands found with each pharmacophore and the total number of unique ligands found by combining the pharmacophores are also illustrated (right graph).


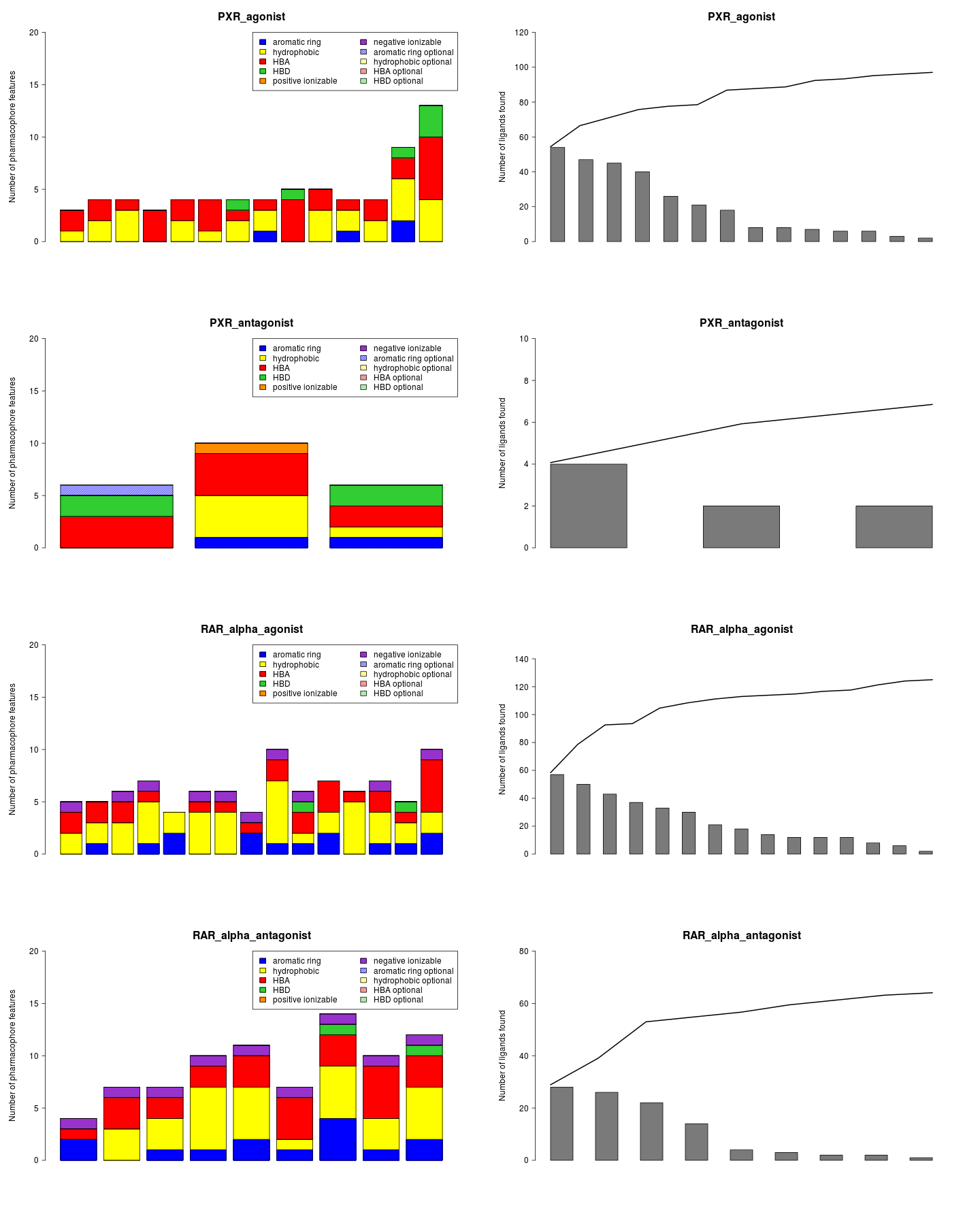


Figure S2H. Representation of the pharmacophore features composition of the “SBLB agonist selective pharmacophores” and “SBLB antagonist selective pharmacophores” combinations for each NRLiSt BDB dataset (left graph). The number of ligands found with each pharmacophore and the total number of unique ligands found by combining the pharmacophores are also illustrated (right graph).


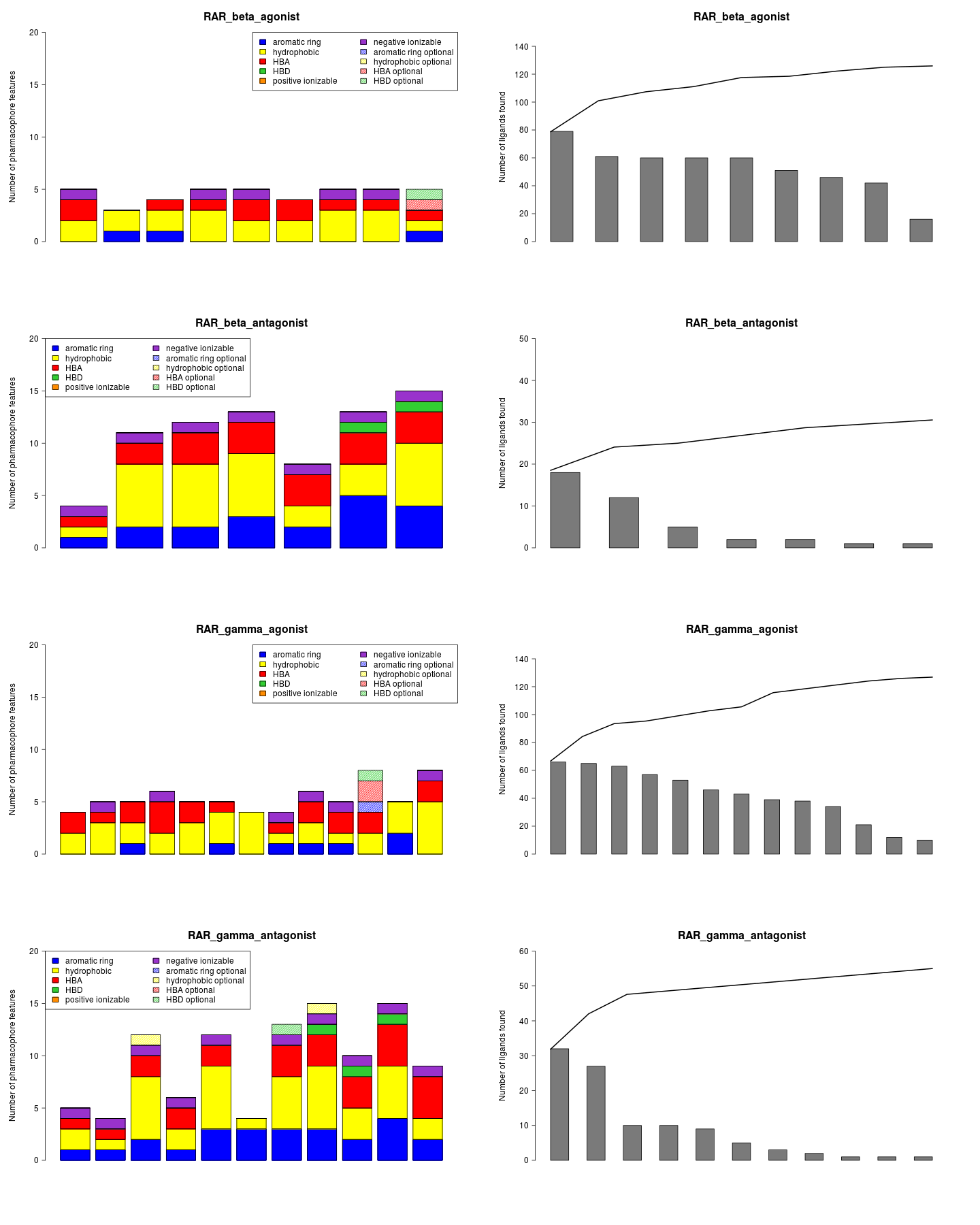


Figure S2I. Representation of the pharmacophore features composition of the “SBLB agonist selective pharmacophores” and “SBLB antagonist selective pharmacophores” combinations for each NRLiSt BDB dataset (left graph). The number of ligands found with each pharmacophore and the total number of unique ligands found by combining the pharmacophores are also illustrated (right graph).


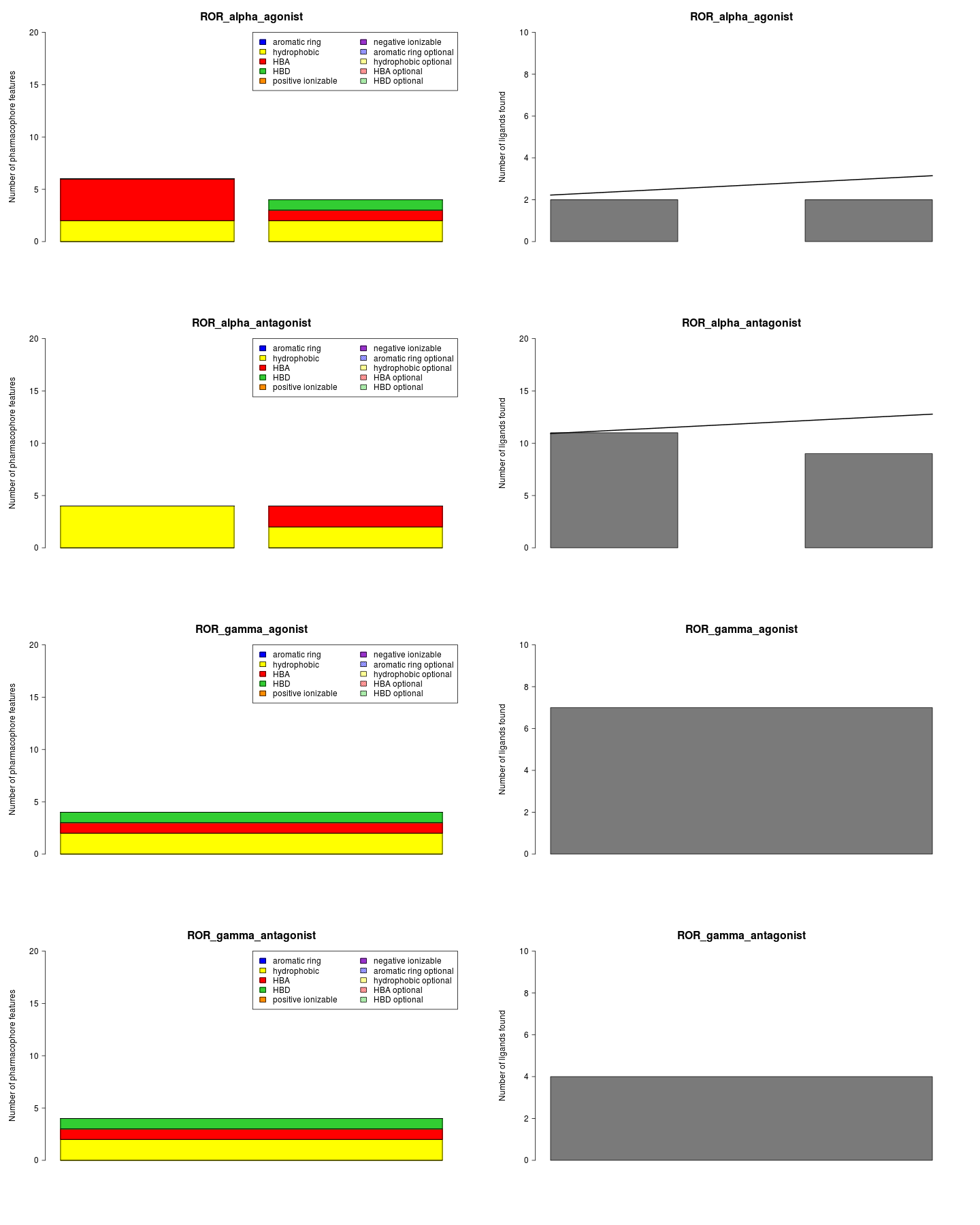


Figure S2J. Representation of the pharmacophore features composition of the “SBLB agonist selective pharmacophores” and “SBLB antagonist selective pharmacophores” combinations for each NRLiSt BDB dataset (left graph). The number of ligands found with each pharmacophore and the total number of unique ligands found by combining the pharmacophores are also illustrated (right graph).


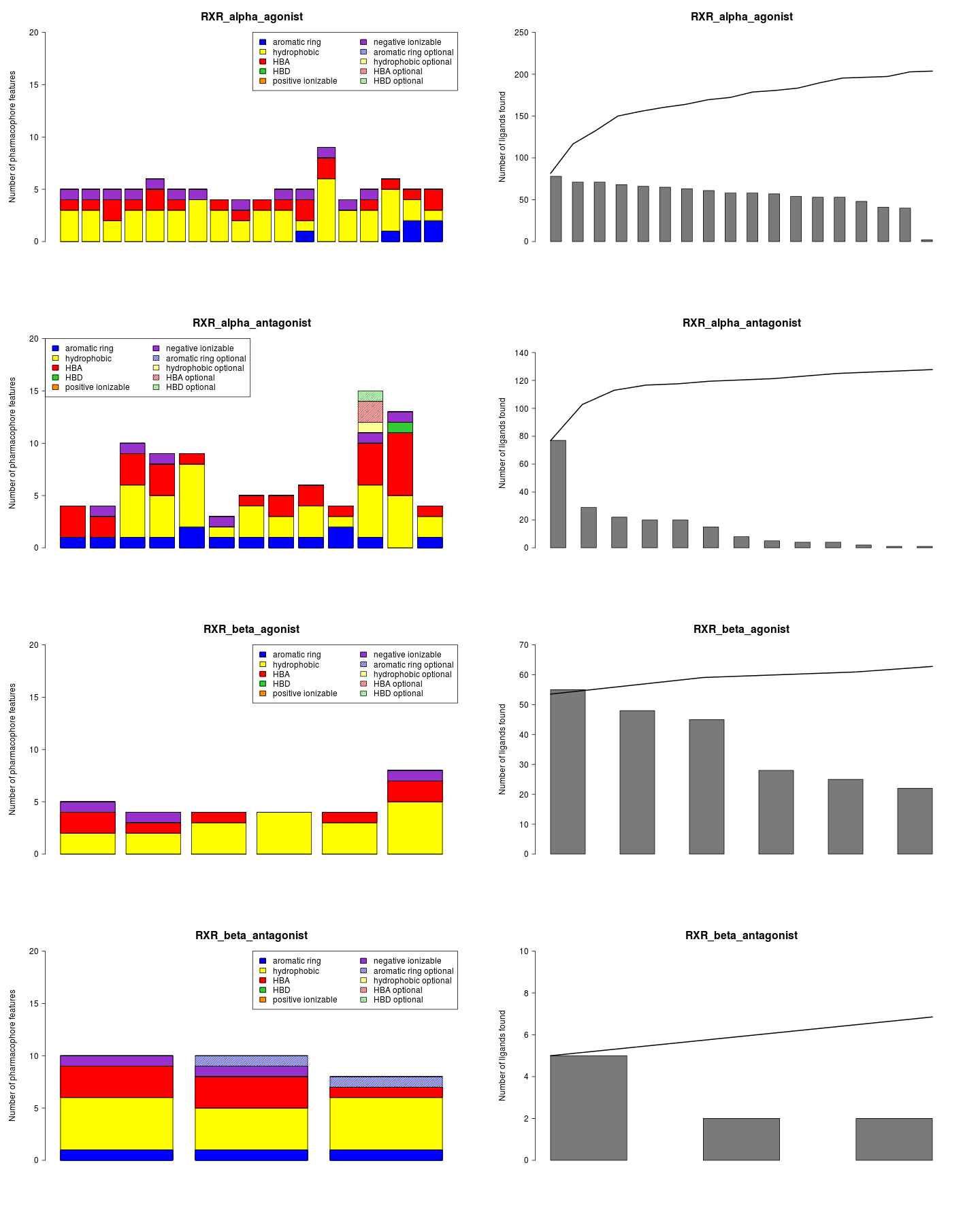


Figure S2K. Representation of the pharmacophore features composition of the “SBLB agonist selective pharmacophores” and “SBLB antagonist selective pharmacophores” combinations for each NRLiSt BDB dataset (left graph). The number of ligands found with each pharmacophore and the total number of unique ligands found by combining the pharmacophores are also illustrated (right graph).


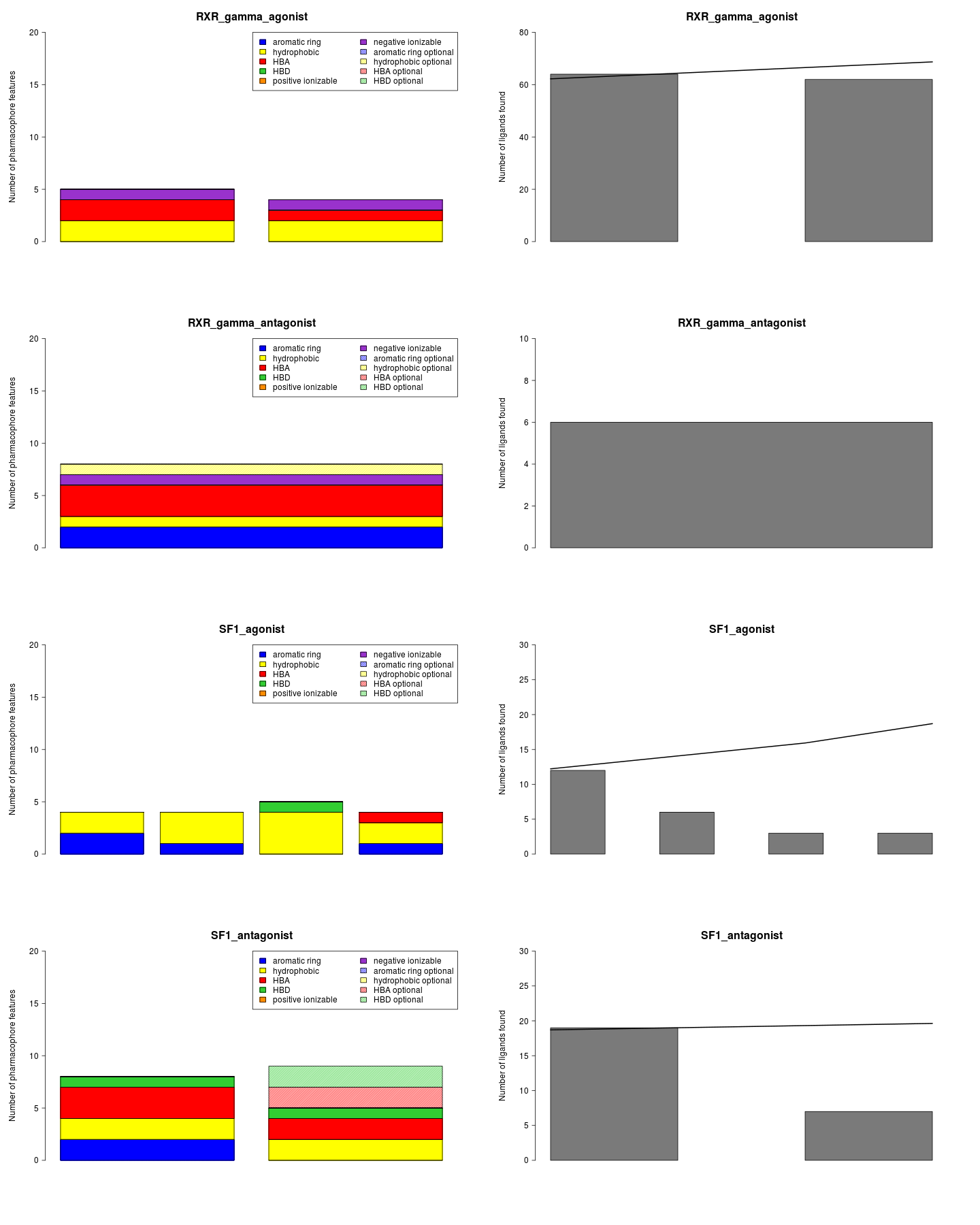


Figure S2L. Representation of the pharmacophore features composition of the “SBLB agonist selective pharmacophores” and “SBLB antagonist selective pharmacophores” combinations for each NRLiSt BDB dataset (left graph). The number of ligands found with each pharmacophore and the total number of unique ligands found by combining the pharmacophores are also illustrated (right graph).


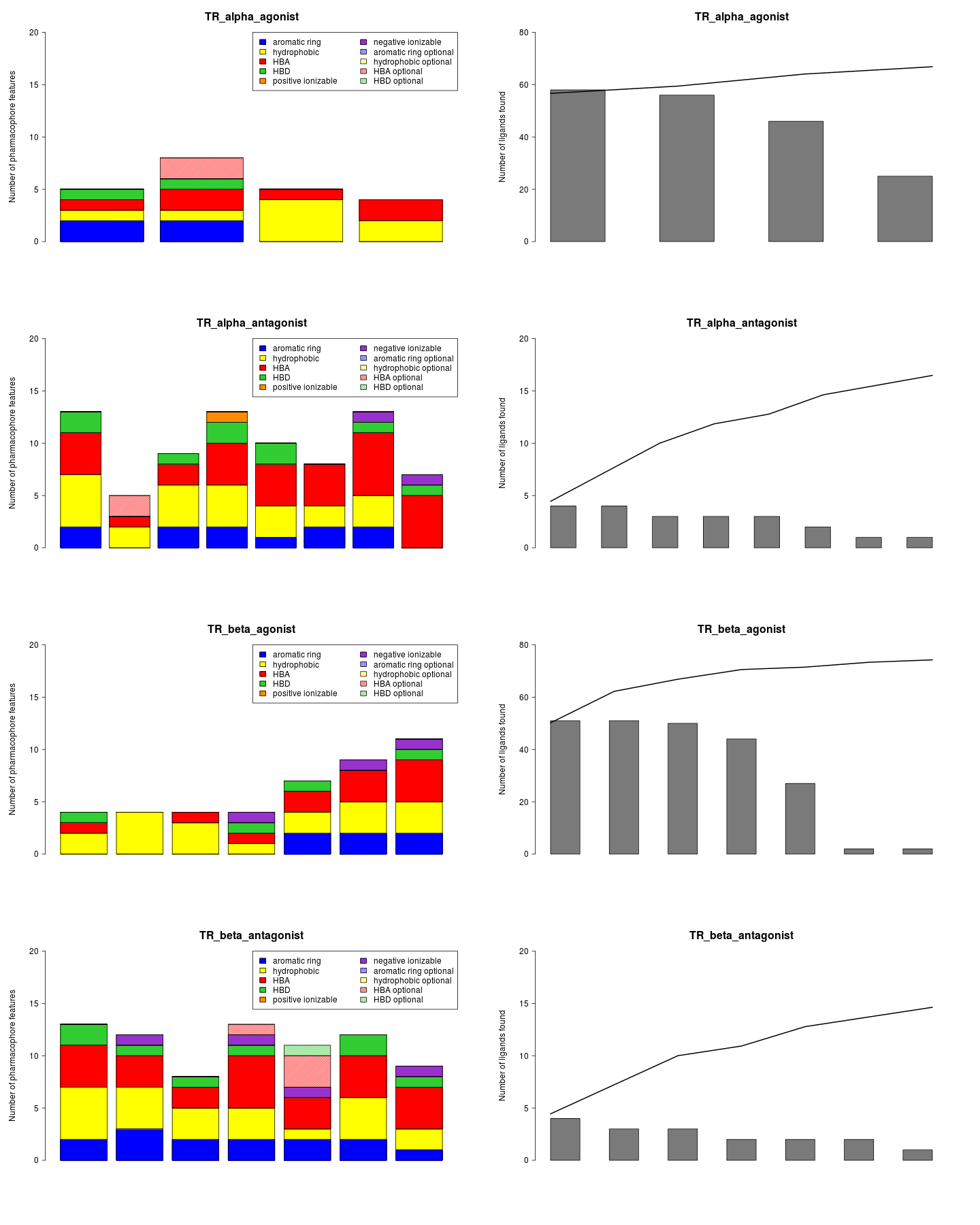


Figure S2M. Representation of the pharmacophore features composition of the “SBLB agonist selective pharmacophores” and “SBLB antagonist selective pharmacophores” combinations for each NRLiSt BDB dataset (left graph). The number of ligands found with each pharmacophore and the total number of unique ligands found by combining the pharmacophores are also illustrated (right graph).


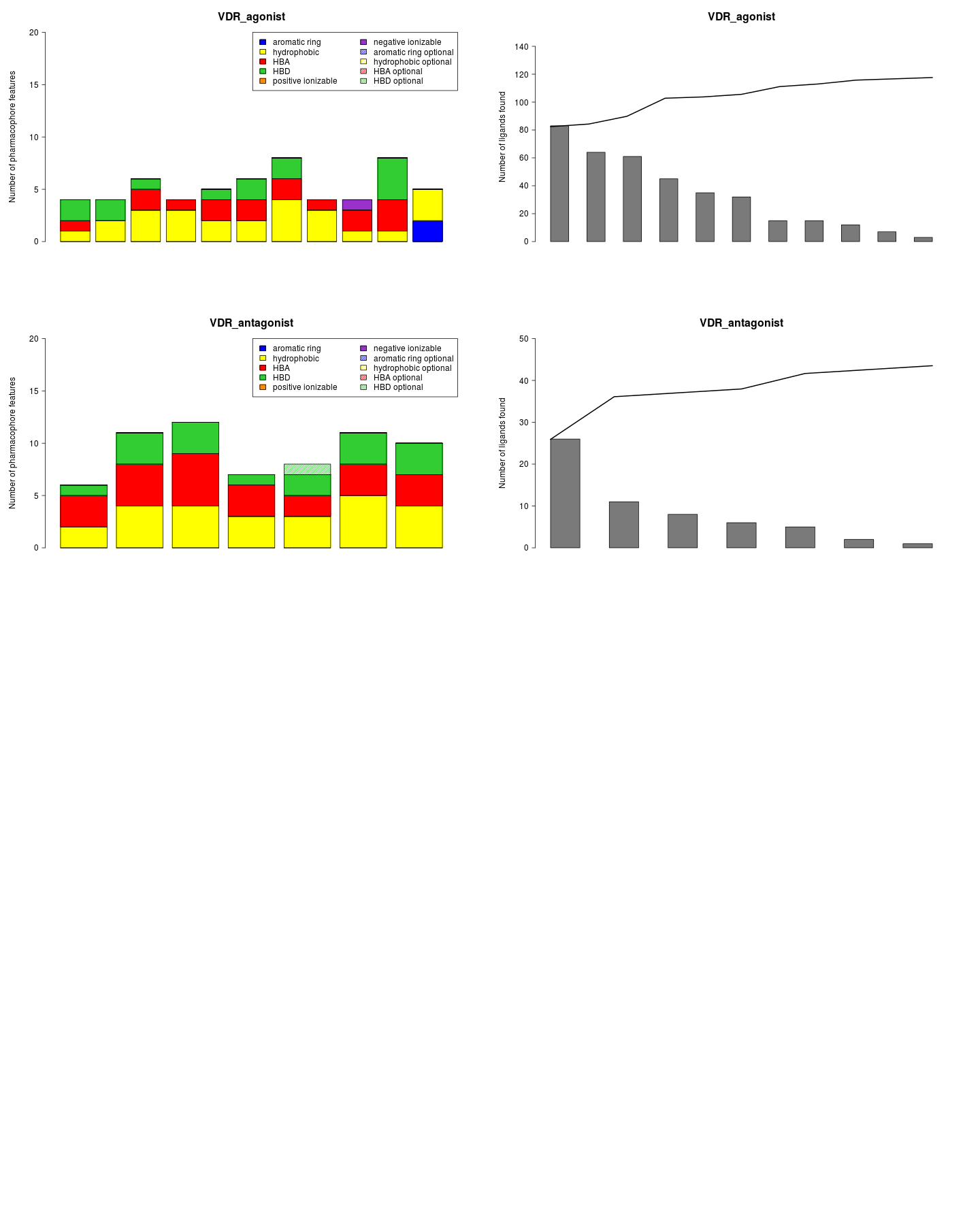


Figure S2N. Representation of the pharmacophore features composition of the “SBLB agonist selective pharmacophores” and “SBLB antagonist selective pharmacophores” combinations for each NRLiSt BDB dataset (left graph). The number of ligands found with each pharmacophore and the total number of unique ligands found by combining the pharmacophores are also illustrated (right graph).


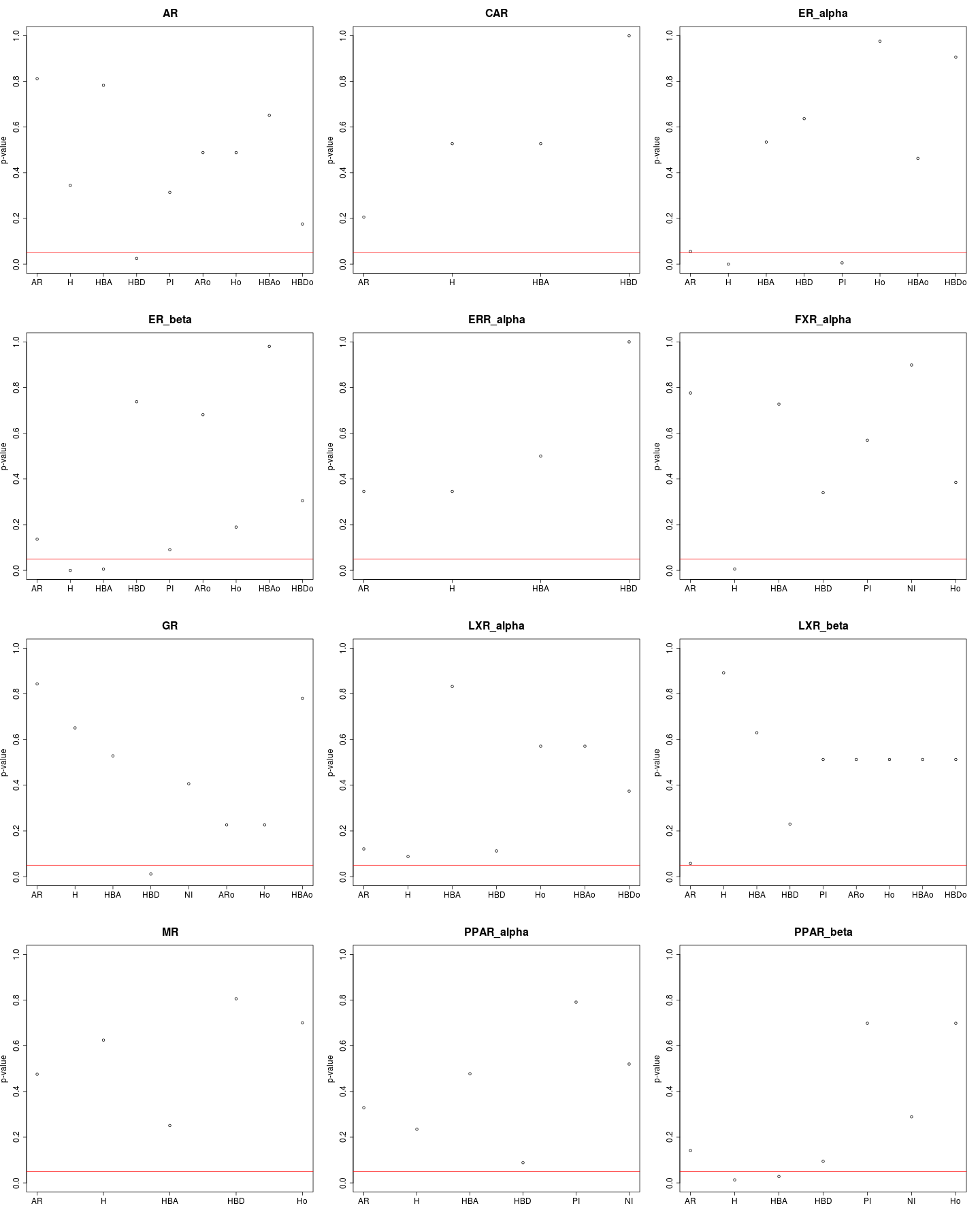


Figure S3A. Comparison of the distribution of pharmacophore features between SBLB agonist selective pharmacophores and SBLB antagonist selective pharmacophores for each NR of the NRLiSt BDB using the Wilcoxon-test. The red line represents the significance threshold (p-value = 0.05).


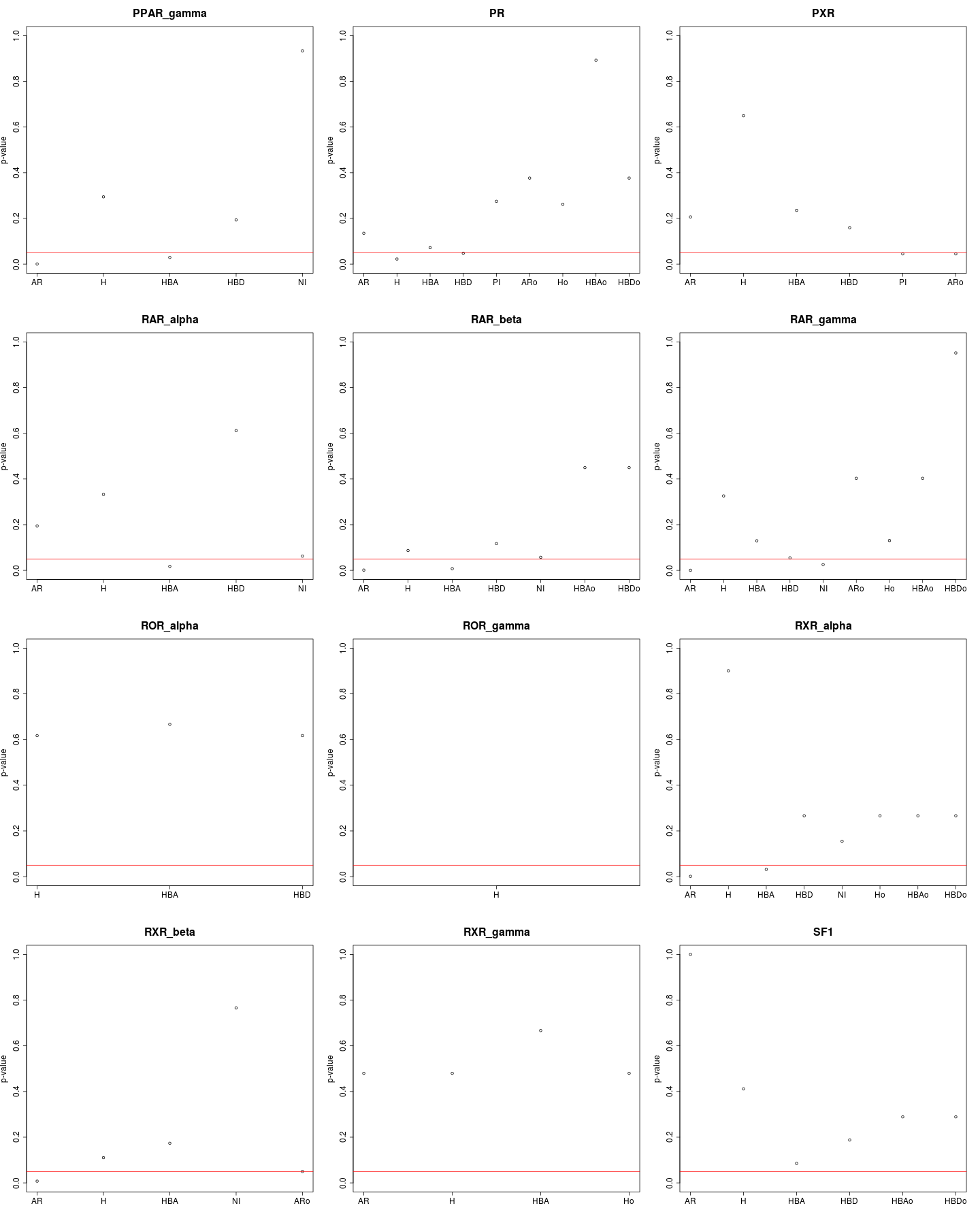


Figure S3B. Comparison of the distribution of pharmacophore features between SBLB agonist selective pharmacophores and SBLB antagonist selective pharmacophores for each NR of the NRLiSt BDB using the Wilcoxon-test. The red line represents the significance threshold (p-value = 0.05).


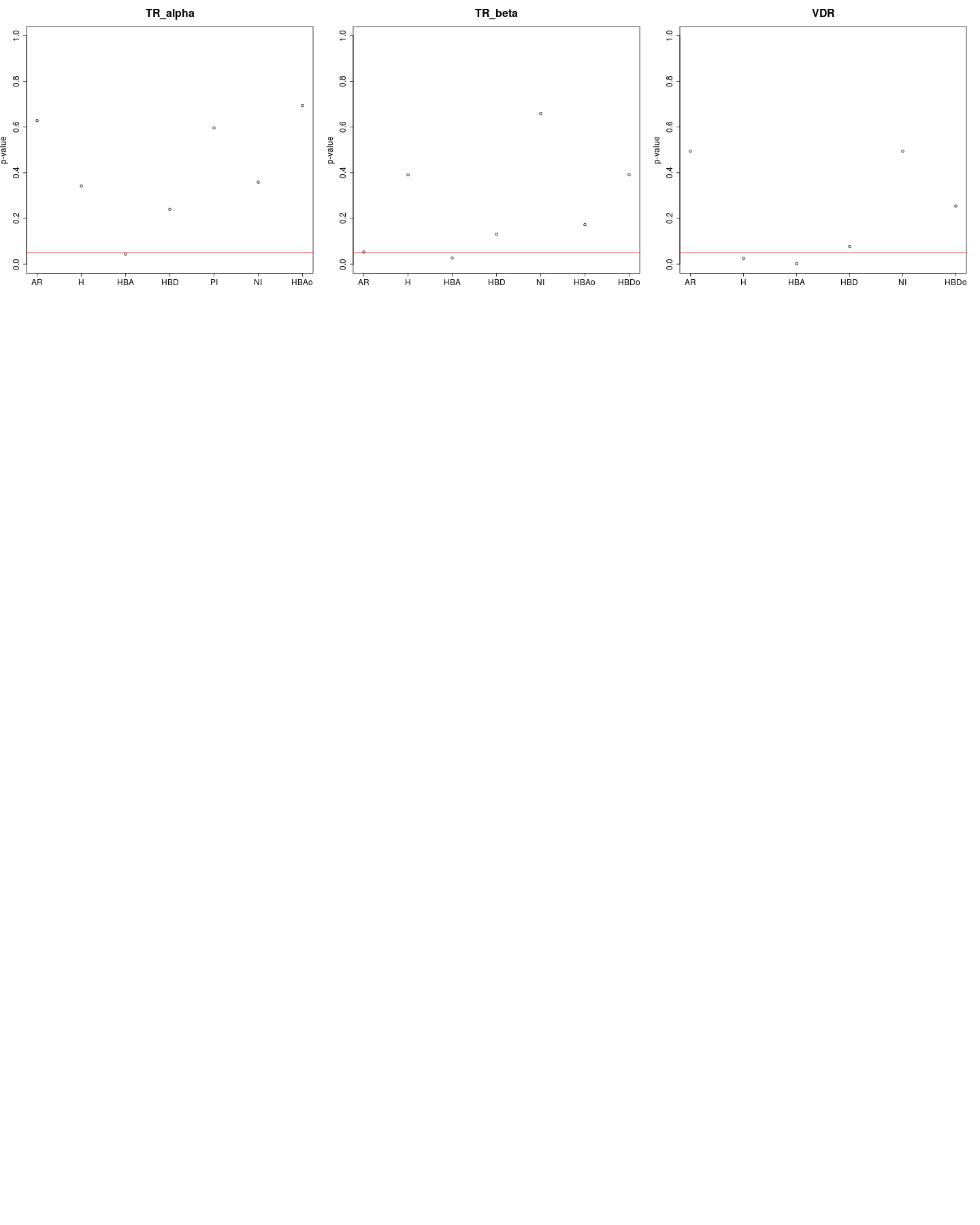


Figure S3C. Comparison of the distribution of pharmacophore features between SBLB agonist selective pharmacophores and SBLB antagonist selective pharmacophores for each NR of the NRLiSt BDB using the Wilcoxon-test. The red line represents the significance threshold (p-value = 0.05).


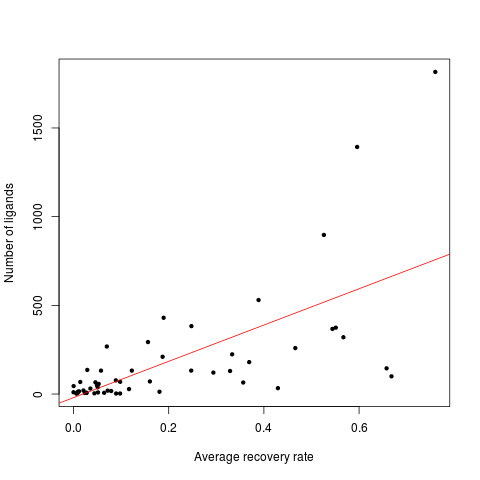


Figure S4. Correlation between the selectivity of a combination of pharmacophore towards their dedicated NRs ligands (average recovery rate against all the others NRLiSt BDB datasets) and the number of ligands in the dataset


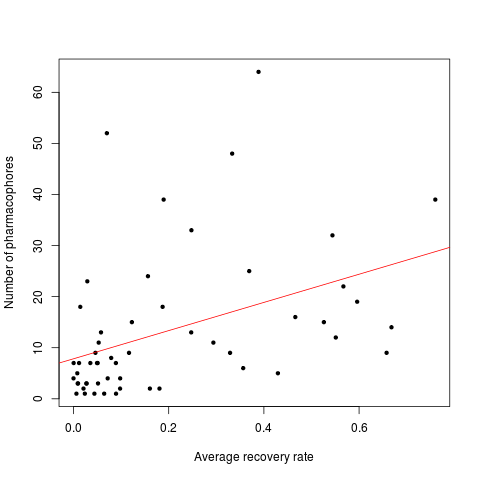


Figure S5. Correlation between the selectivity of a combination of pharmacophore towards their dedicated NRs ligands (average recovery rate against all the others NRLiSt BDB datasets) and the number of pharmacophores included in the combination


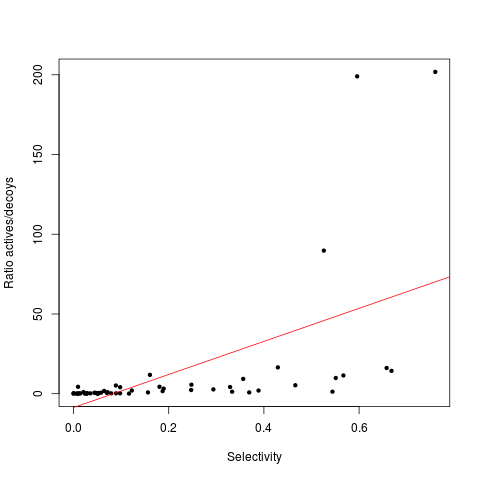


Figure S6. Correlation between the selectivity of a combination of pharmacophore towards their dedicated NRs ligands (average recovery rate against all the others NRLiSt BDB datasets) and the active ligands over decoys ratio


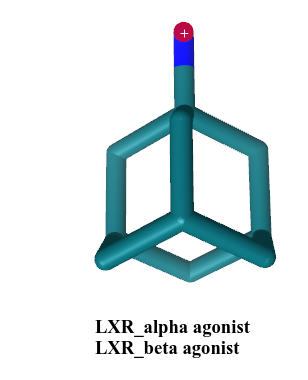


Figure S7. Structure of the LXR_alpha and LXR_beta agonist that could only be represented by a pharmacophore formed of 2 non-independent features


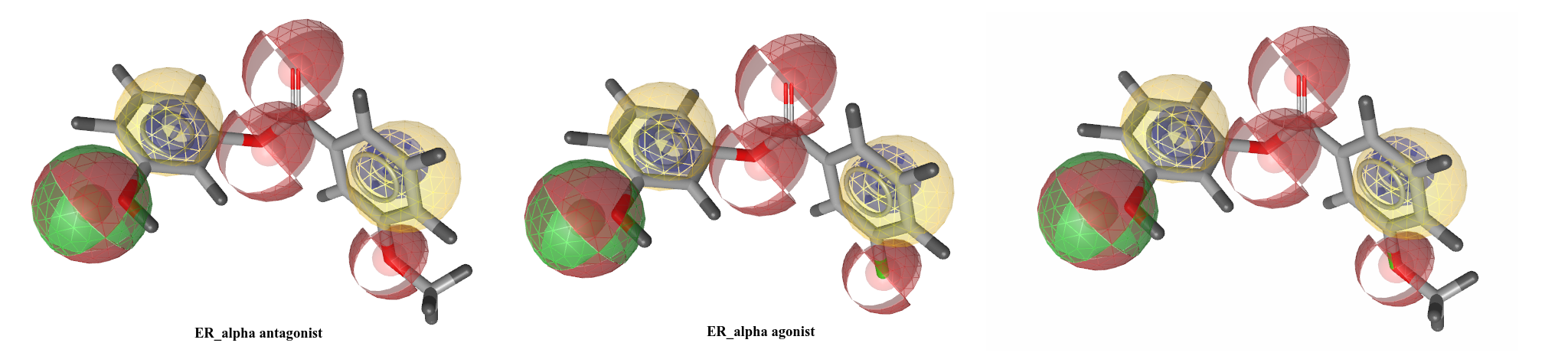


Figure S8. Structure of the ER_alpha antagonist ligand and ER_alpha agonist ligand that could not be separated using 3D pharmacophore models


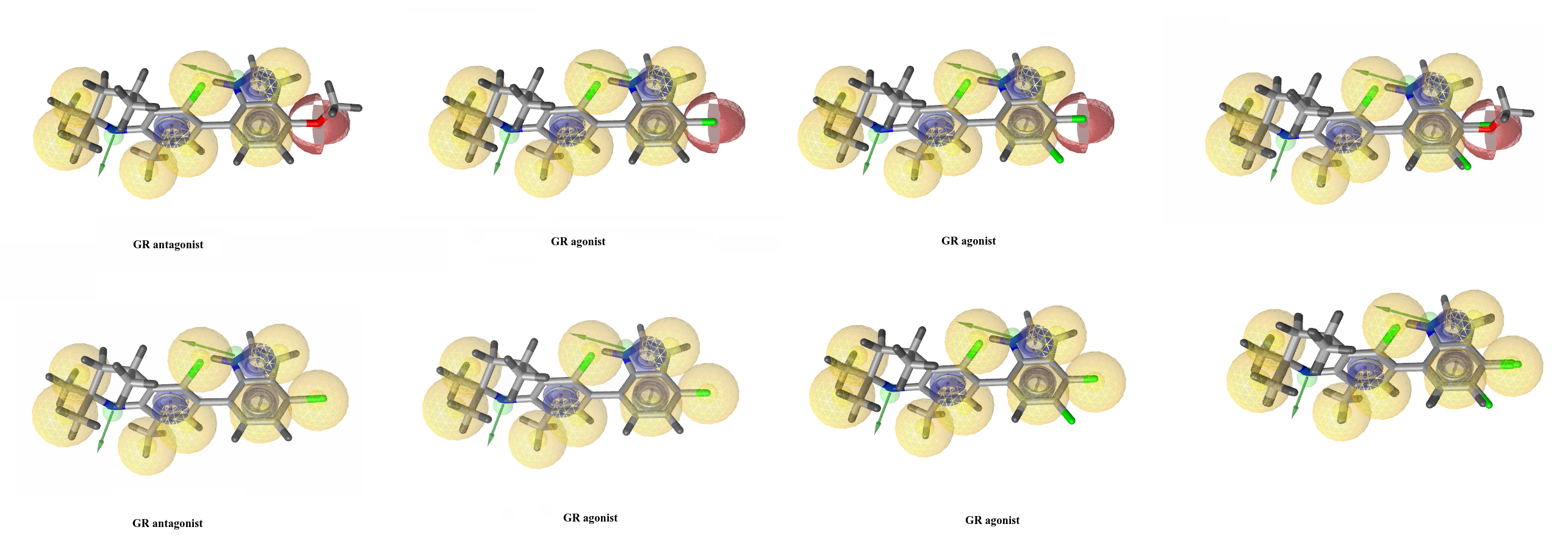


Figure S9. Structure of the GR antagonist ligands and GR agonist ligands that could not be separated using 3D pharmacophore models


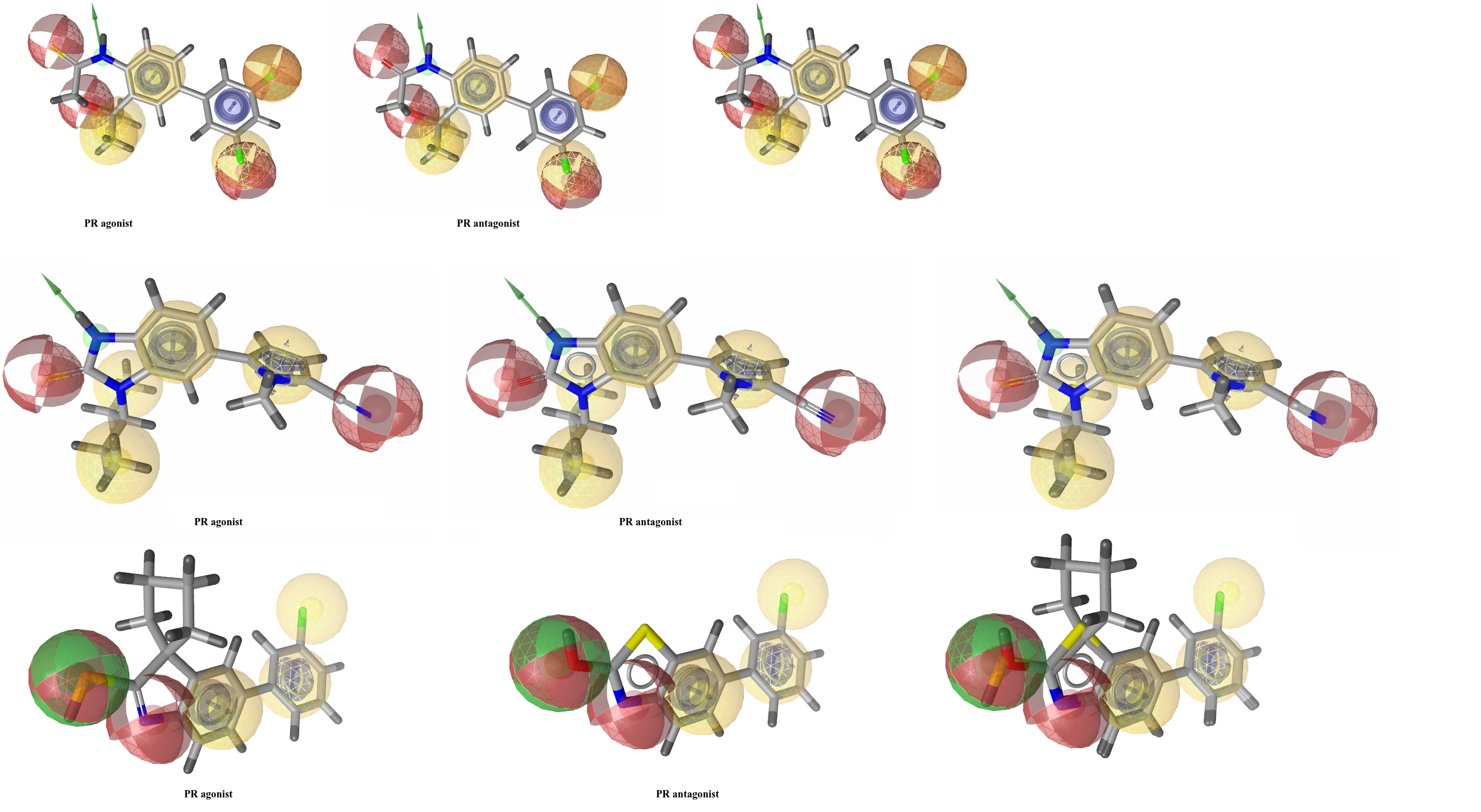


Figure S10. (Continued next page)


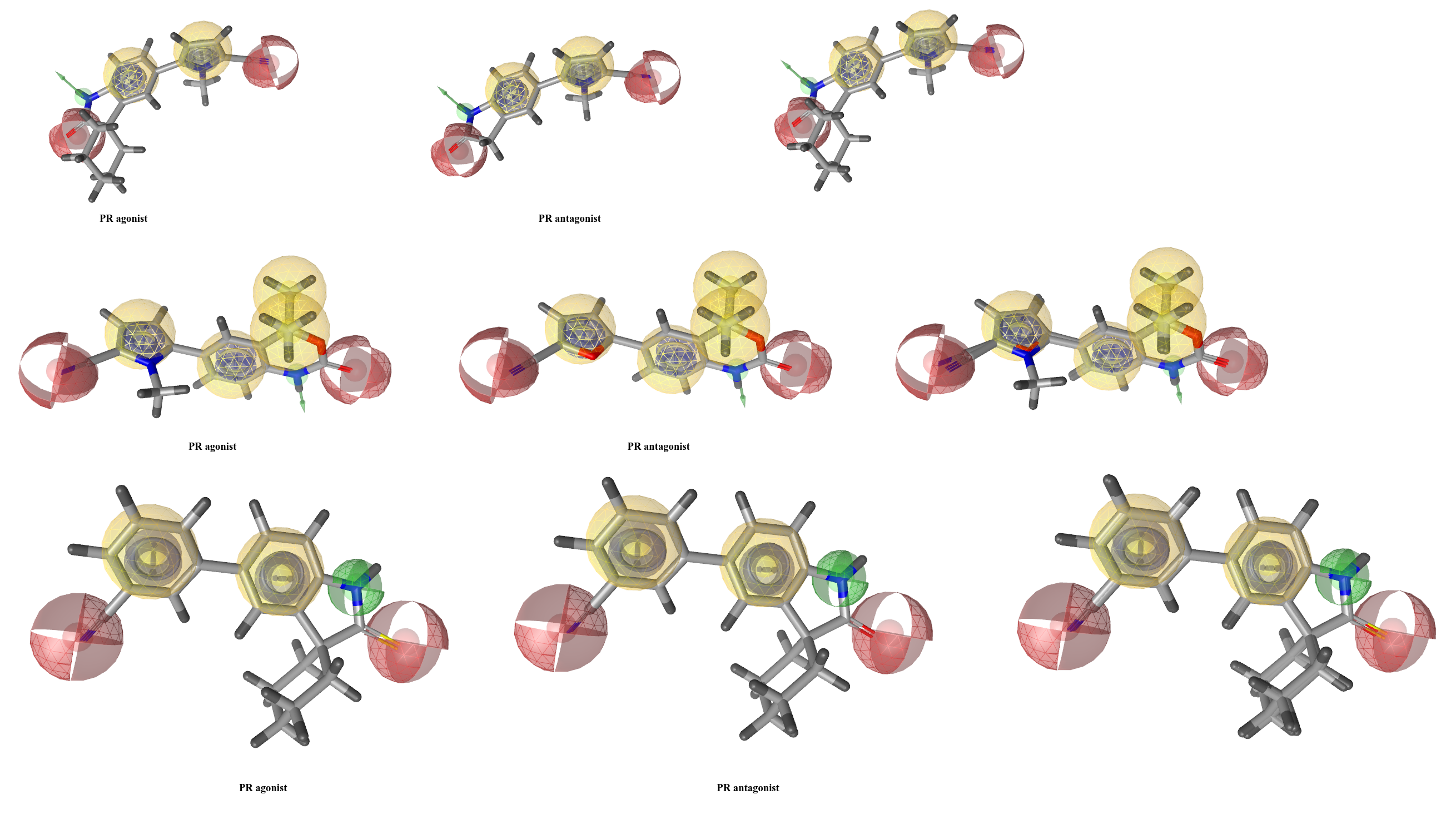


Figure S10. (Continued next page)


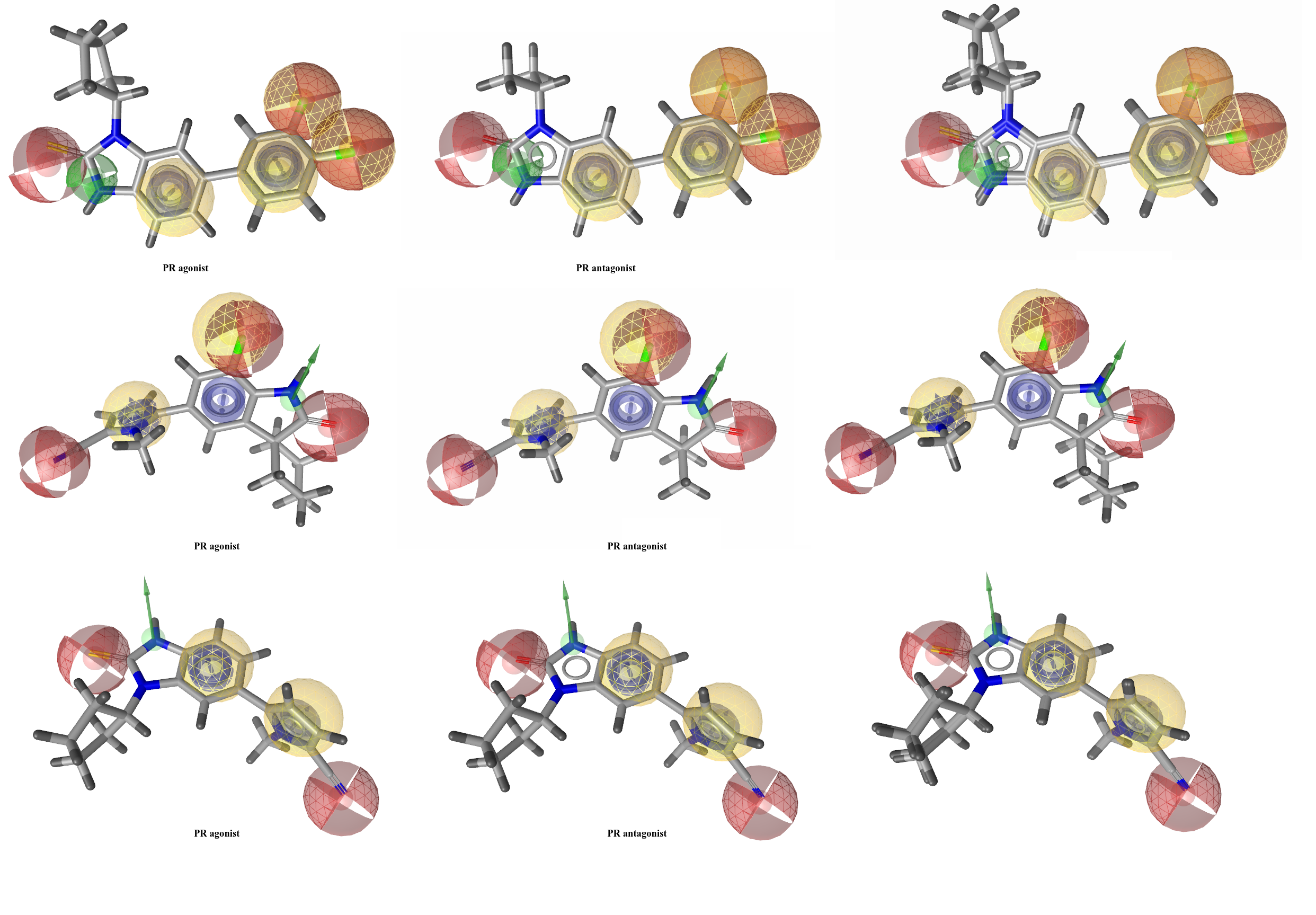


Figure S10. Structure of the PR agonist ligands and PR antagonist ligands that could not be separated using 3D pharmacophore models


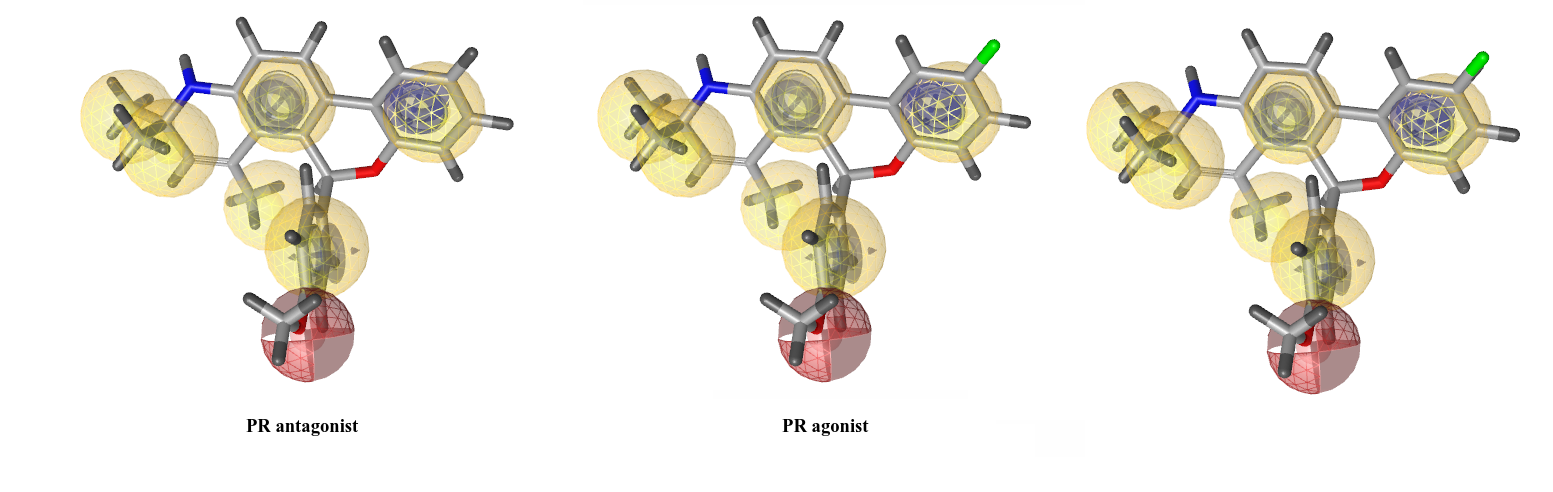


Figure S11. Structure of the PR antagonist ligand and PR agonist ligand that could not be separated using 3D pharmacophore models


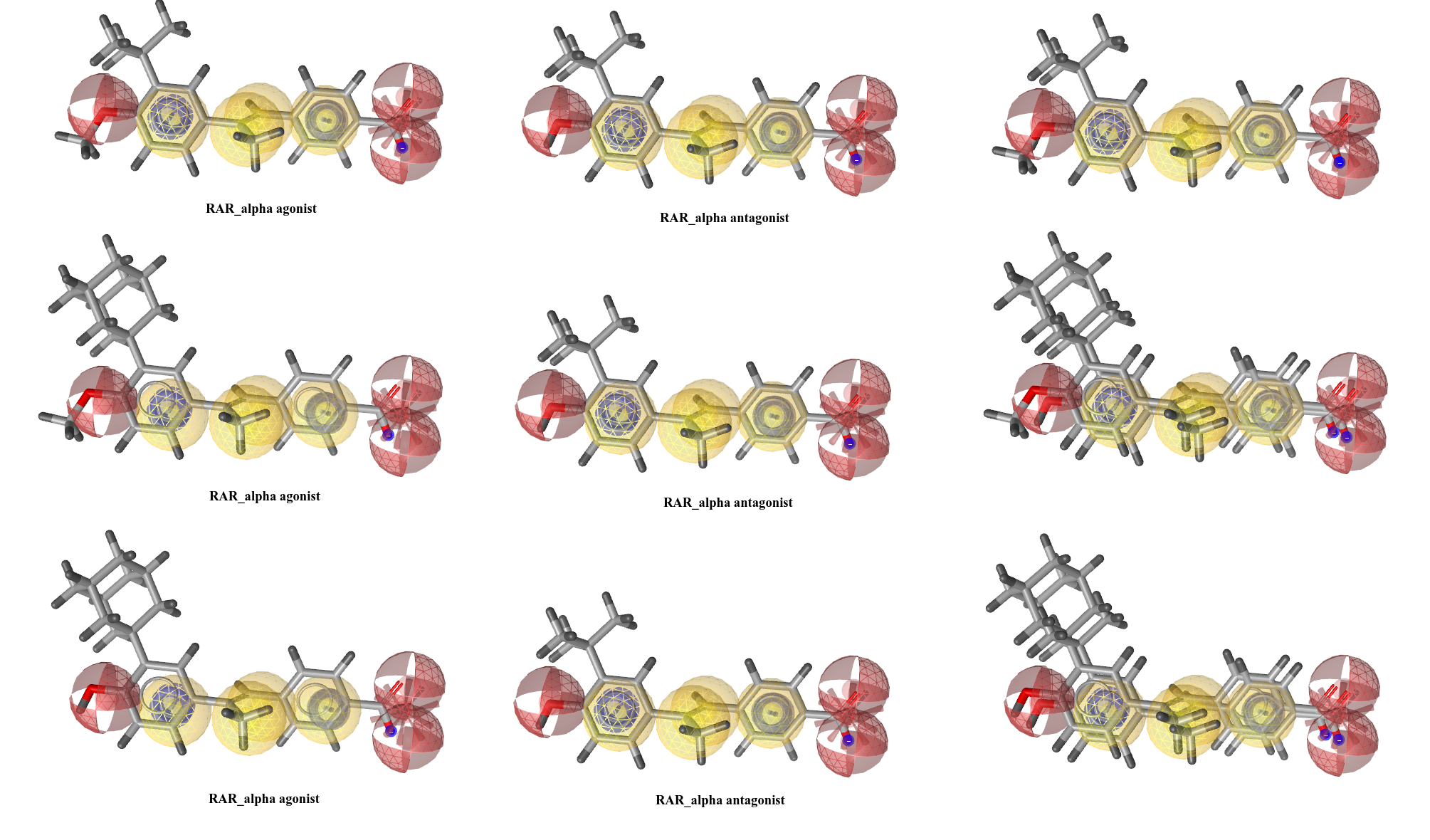


Figure S12. Structure of the RAR_alpha agonist ligands and RAR_alpha antagonist ligands that could not be separated using 3D pharmacophore models


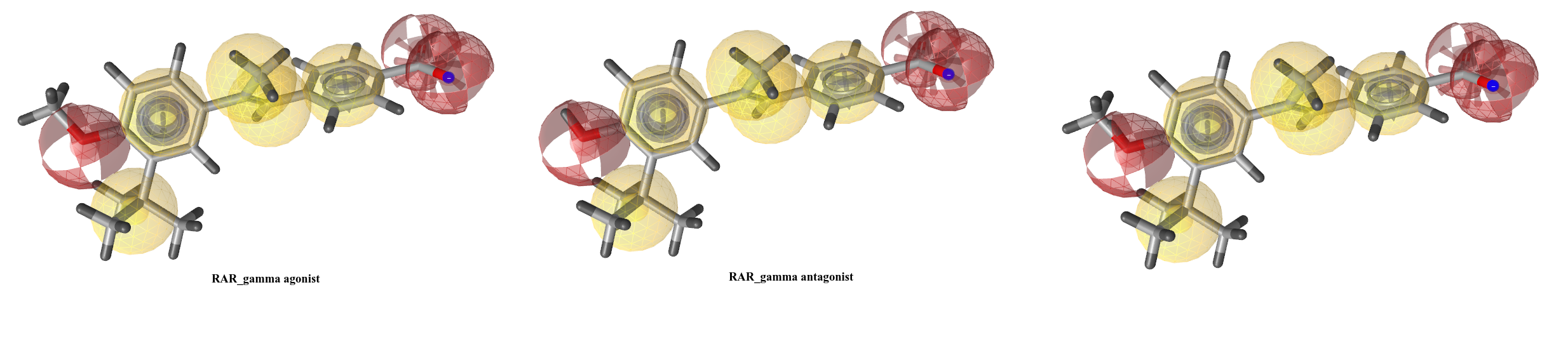


Figure S13. Structure of the RAR_gamma agonist ligand and RAR_gamma antagonist ligands that could not be separated using 3D pharmacophore models


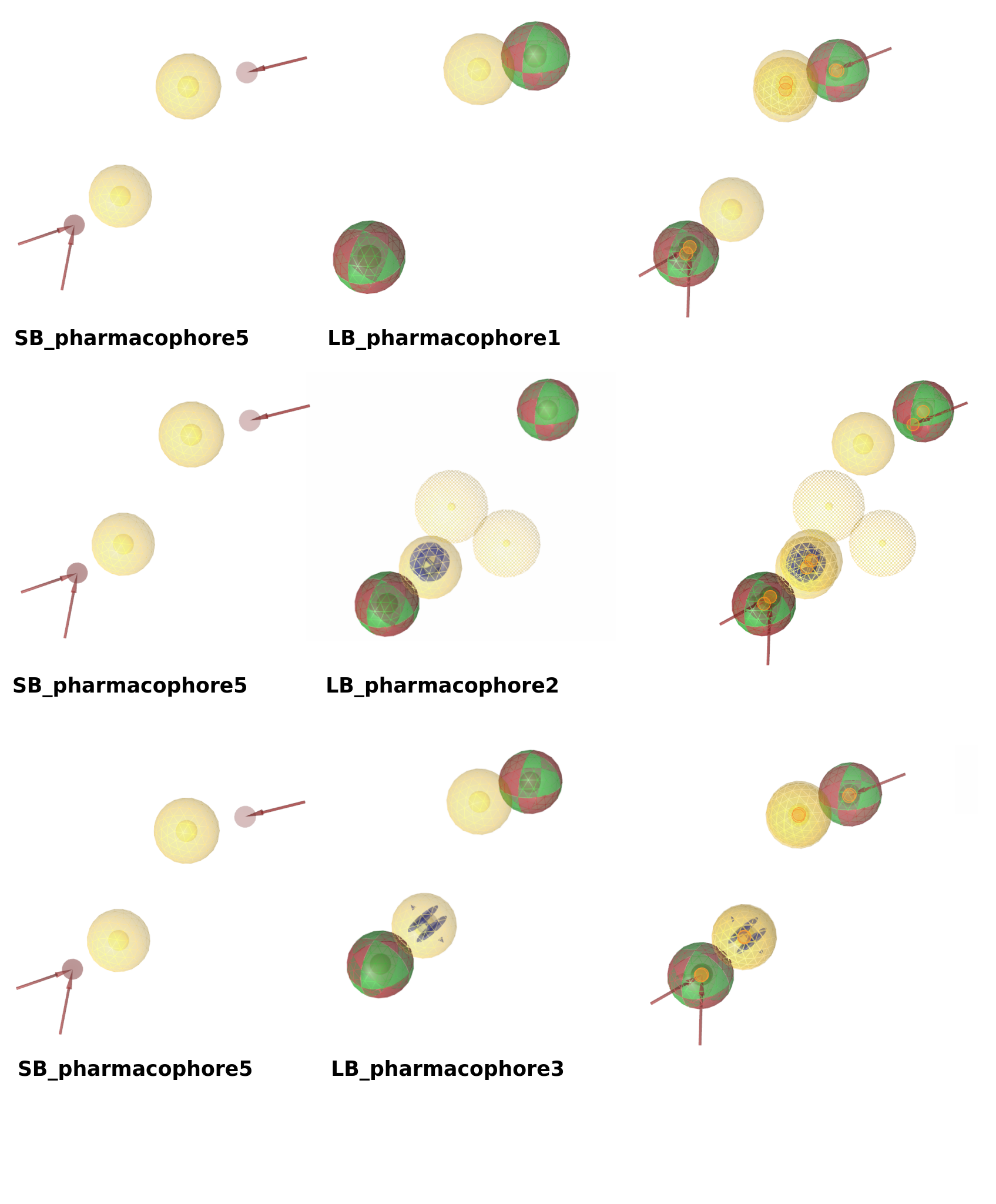
Figure S14. (Continued next page)


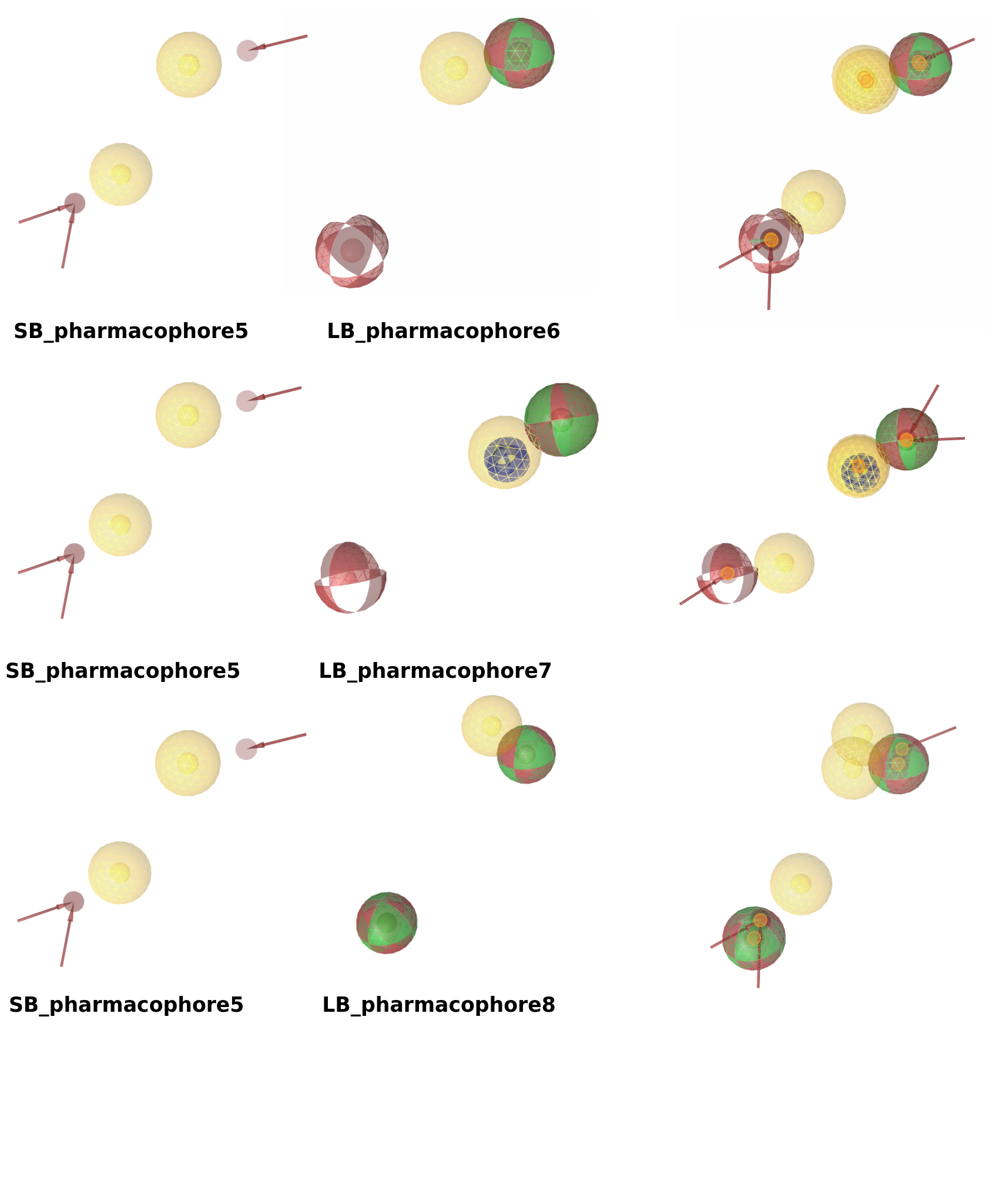
Figure S14. (Continued next page)


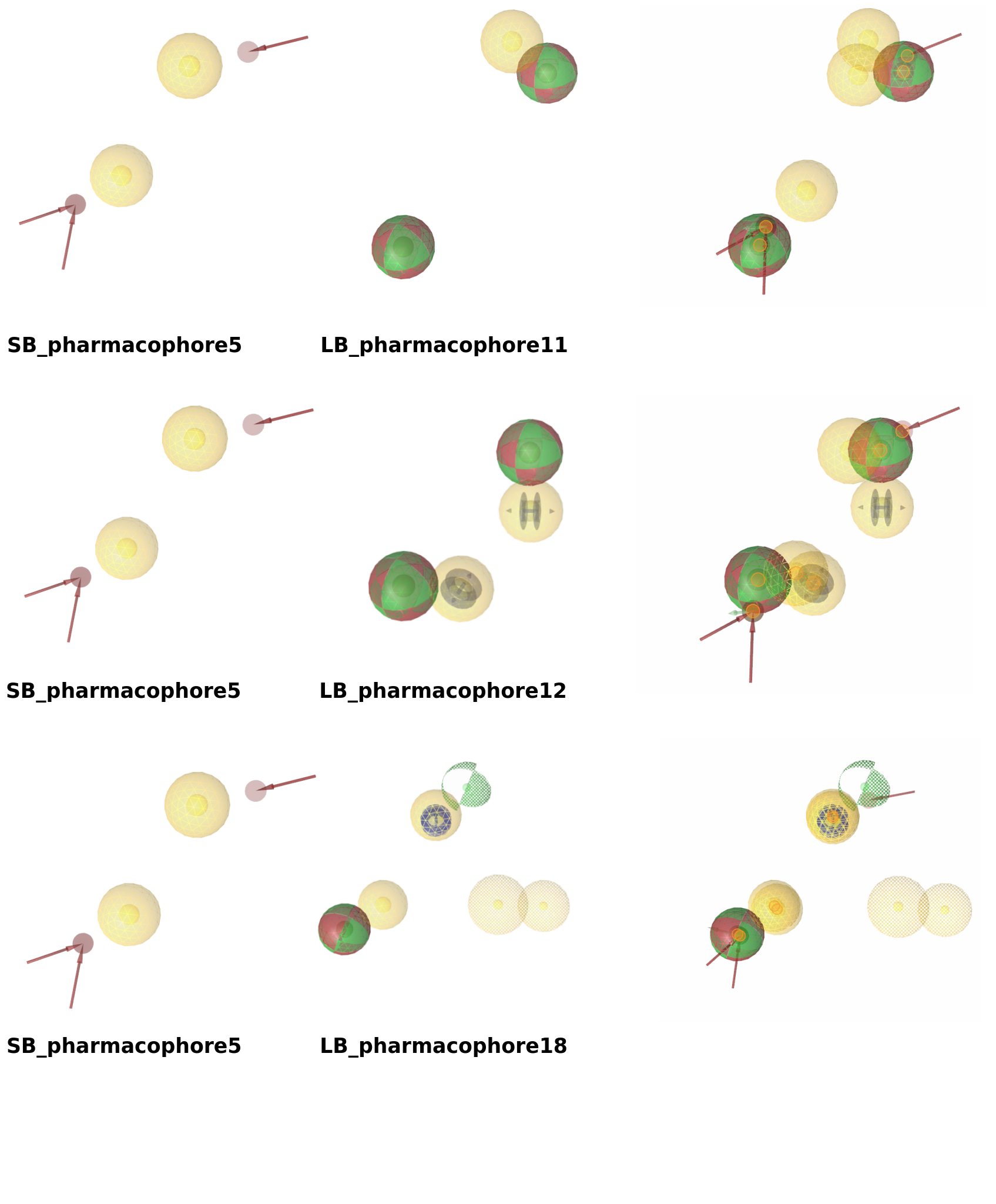
 Figure S14. (Continued next page)


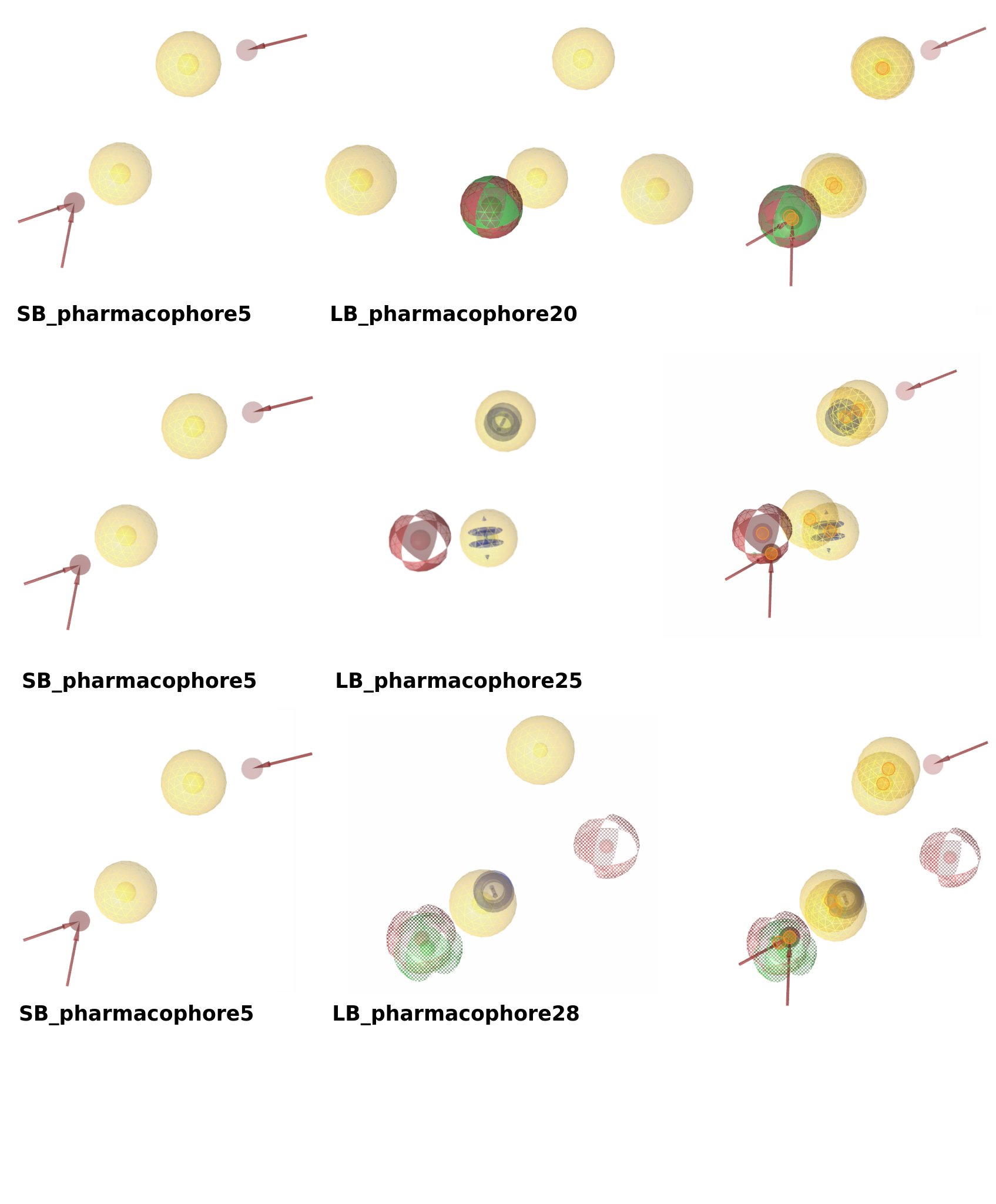
 Figure S14. (Continued next page)


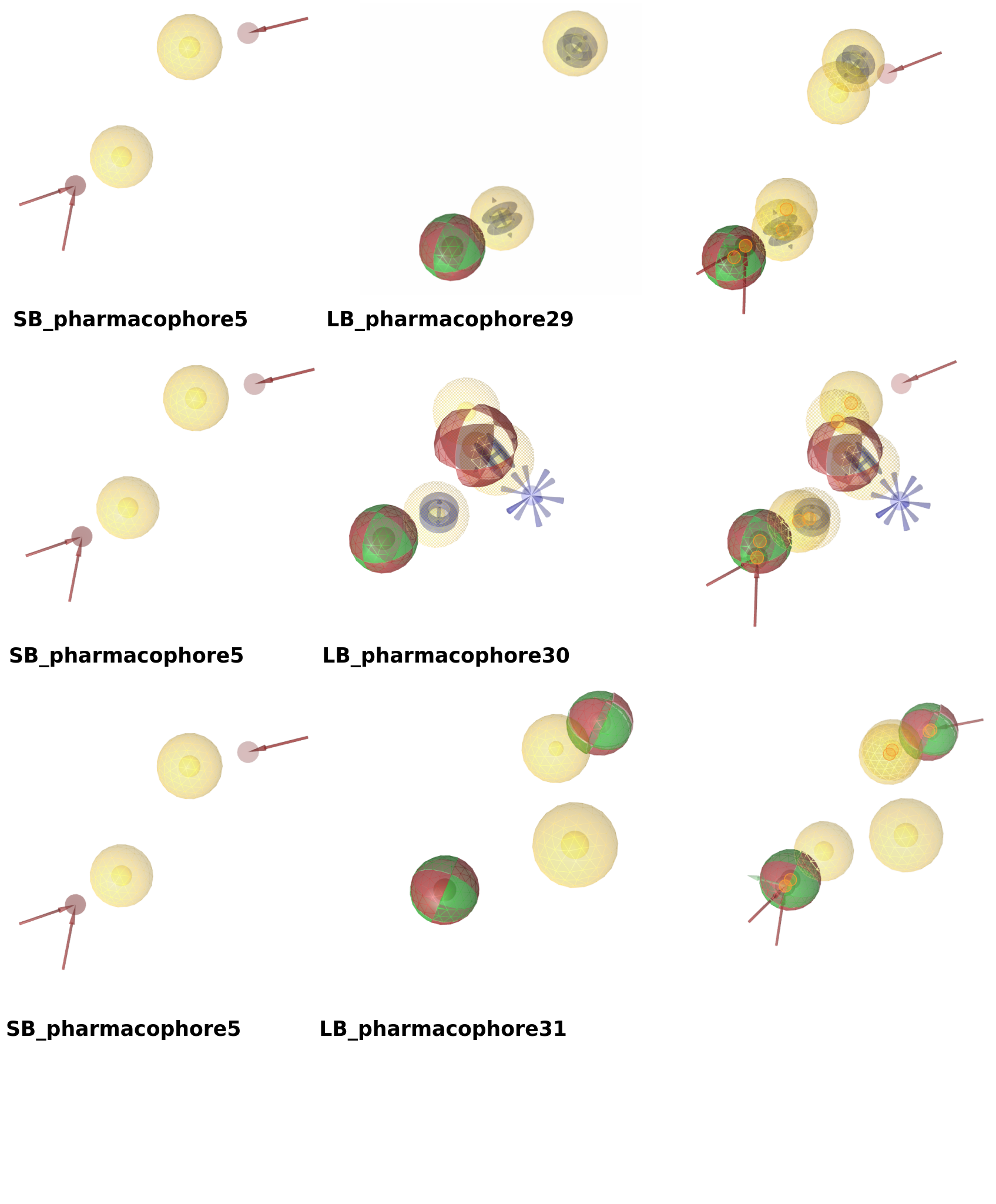
 Figure S14. (Continued next page)


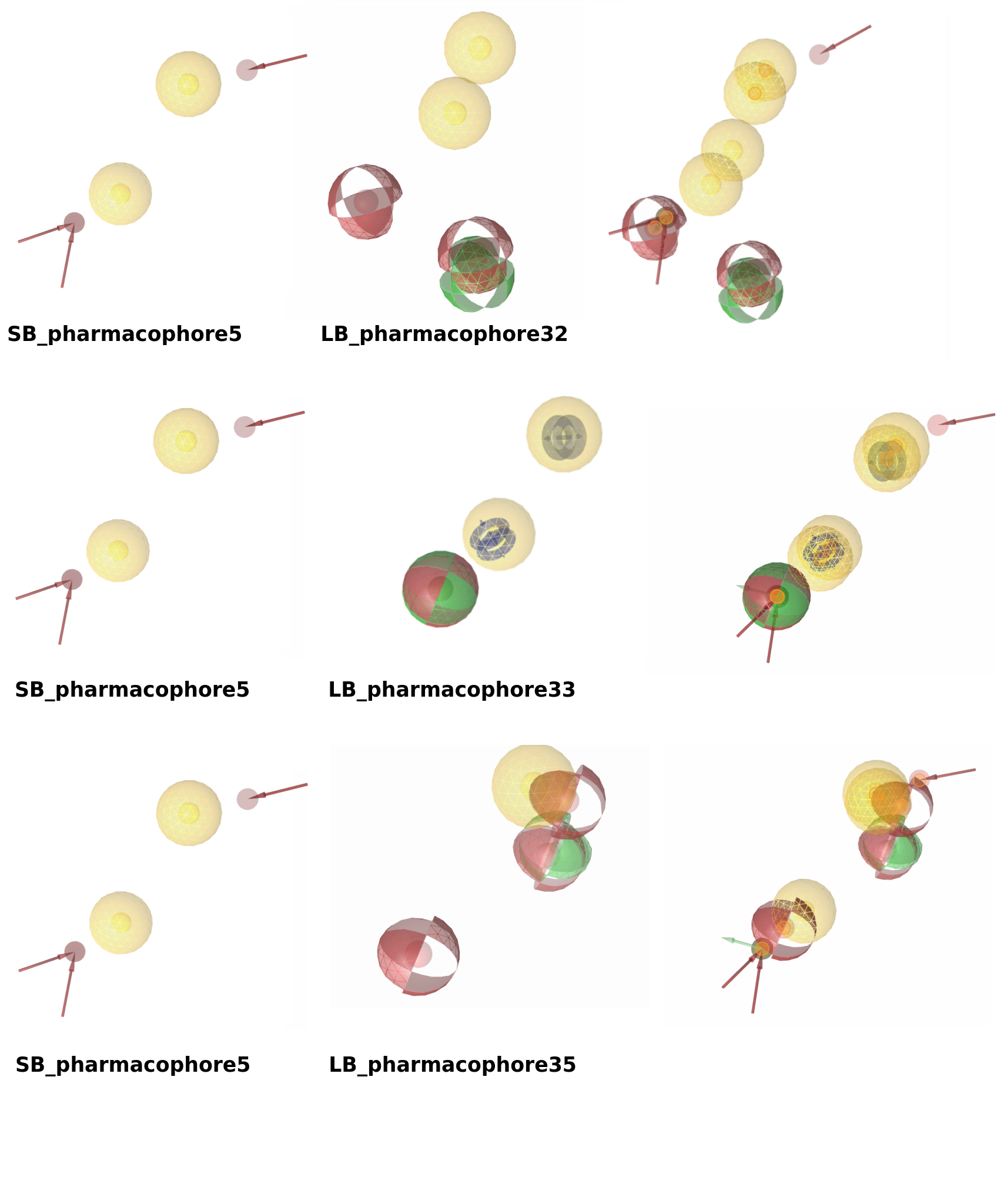
 Figure S14. (Continued next page)


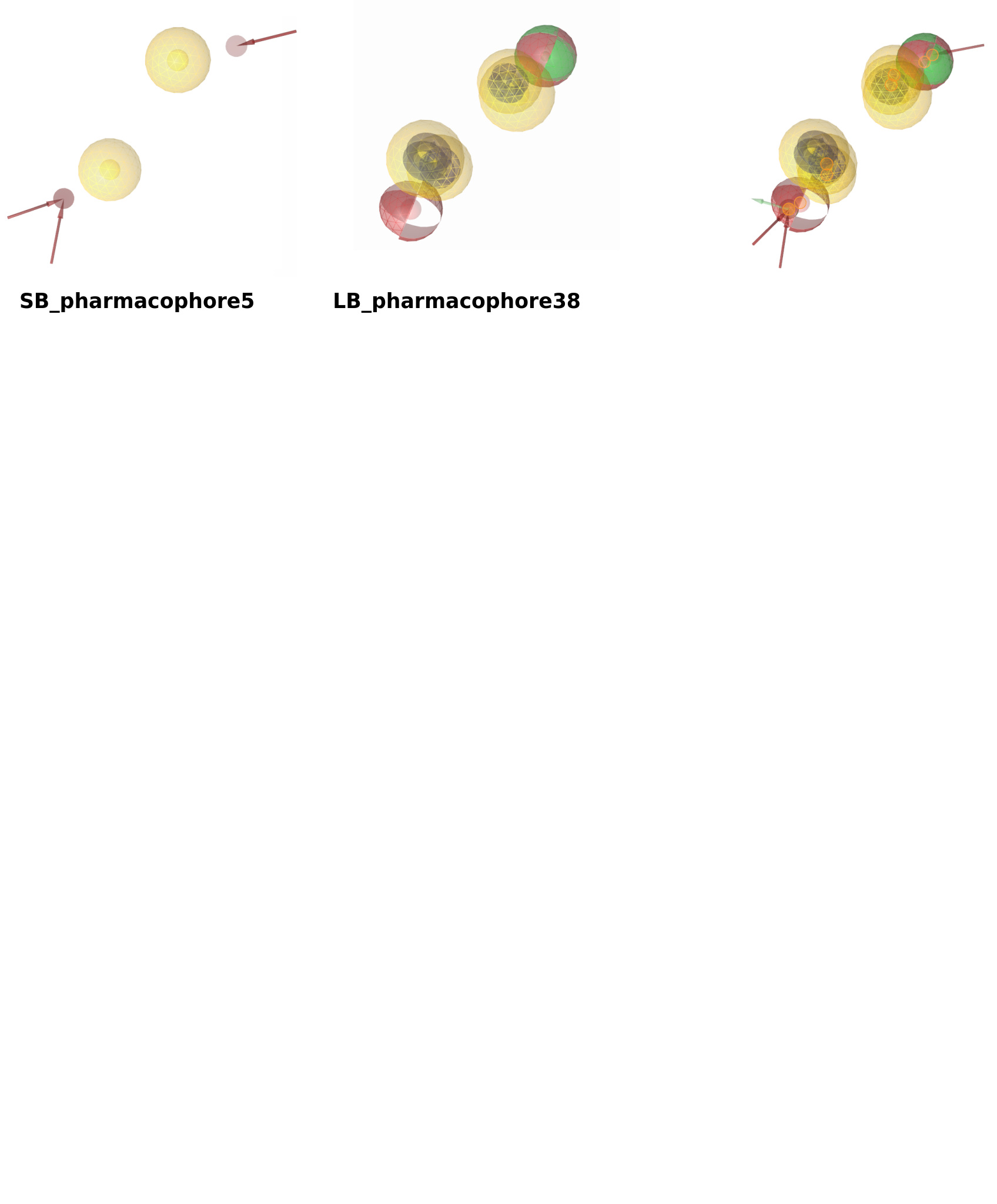
Figure S14. Overlapping features between the SB_pharmacophore5 ER_alpha agonist selective pharmacophore and LB ER_alpha agonist selective pharmacophores (reference point tethers are represented by the orange circle on the alignment, right graph)

Figure S15. (Continued next page)

Figure S15. (Continued next page)

Figure S15. (Continued next page)

Figure S15. (Continued next page)

Figure S15. (Continued next page)

Figure S15. (Continued next page)

Figure S15. (Continued next page)

Figure S15. (Continued next page)

Figure S15. (Continued next page)

Figure S15. (Continued next page)

Figure S15. (Continued next page)

Figure S15. (Continued next page)

Figure S15. (Continued next page)

Figure S15. (Continued next page)

Figure S15. (Continued next page)

Figure S15. (Continued next page)

Figure S15. (Continued next page)

Figure S15. (Continued next page)

Figure S15. (Continued next page)

Figure S15. (Continued next page)

Figure S15. (Continued next page)

Figure S15. (Continued next page)

Figure S15. (Continued next page)

Figure S15. (Continued next page)

Figure S15. (Continued next page)

Figure S15. (Continued next page)

Figure S15. Correlation between the number of pharmacophore features and the number of ligands retrieved by each pharmacophore

|  | Pharmacophore features | | | | | | | | | | Number of positive and negative hits | | |
| --- | --- | --- | --- | --- | --- | --- | --- | --- | --- | --- | --- | --- | --- |
|  | AR | H | HBA | HBD | PI | NI | AR opt. | H opt. | HBA opt. | HBD opt. | Nbr of agonist ligands found | Accumulated nbr of unique agonist ligands found | Nbr of antagonist ligands found |
| LB_pharmacophore1 | 0 | 2 | 1 | 1 | 0 | 0 | 0 | 0 | 0 | 0 | 56 | 56 | 0 |
| SB_pharmacophore2 | 0 | 2 | 2 | 0 | 0 | 0 | 0 | 0 | 0 | 0 | 38 | 78 | 0 |
| LB_pharmacophore3 | 2 | 2 | 4 | 1 | 0 | 0 | 0 | 0 | 0 | 0 | 35 | 94 | 0 |
| LB_pharmacophore4 | 0 | 1 | 3 | 1 | 0 | 0 | 0 | 0 | 0 | 0 | 35 | 110 | 0 |
| SB_pharmacophore5 | 0 | 0 | 3 | 1 | 0 | 0 | 0 | 0 | 0 | 0 | 33 | 122 | 0 |
| LB_pharmacophore6 | 2 | 1 | 5 | 1 | 0 | 0 | 0 | 0 | 0 | 0 | 30 | 132 | 0 |
| LB_pharmacophore7 | 0 | 2 | 1 | 0 | 0 | 0 | 0 | 0 | 1 | 2 | 28 | 137 | 0 |
| SB_pharmacophore8 | 0 | 1 | 3 | 1 | 0 | 0 | 0 | 0 | 0 | 0 | 27 | 138 | 0 |
| LB_pharmacophore9 | 0 | 1 | 2 | 1 | 0 | 0 | 0 | 0 | 0 | 0 | 27 | 144 | 0 |
| SB_pharmacophore10 | 0 | 1 | 1 | 2 | 0 | 0 | 0 | 0 | 0 | 0 | 22 | 147 | 0 |
| LB_pharmacophore11 | 2 | 3 | 1 | 1 | 0 | 0 | 0 | 0 | 0 | 0 | 22 | 149 | 0 |
| LB_pharmacophore12 | 0 | 2 | 2 | 0 | 0 | 0 | 0 | 0 | 0 | 0 | 21 | 150 | 0 |
| SB_pharmacophore13 | 0 | 1 | 2 | 1 | 0 | 0 | 0 | 0 | 0 | 0 | 12 | 154 | 0 |
| LB_pharmacophore14 | 0 | 4 | 0 | 0 | 0 | 0 | 0 | 0 | 0 | 0 | 12 | 155 | 0 |
| LB_pharmacophore15 | 2 | 1 | 4 | 2 | 0 | 0 | 0 | 0 | 0 | 0 | 9 | 161 | 0 |
| LB_pharmacophore16 | 2 | 2 | 4 | 0 | 0 | 0 | 0 | 0 | 0 | 0 | 9 | 167 | 0 |
| LB_pharmacophore17 | 0 | 3 | 2 | 0 | 0 | 0 | 0 | 0 | 0 | 0 | 7 | 168 | 0 |
| LB_pharmacophore18 | 2 | 2 | 4 | 1 | 0 | 0 | 0 | 0 | 0 | 0 | 3 | 170 | 0 |
| LB_pharmacophore19 | 1 | 1 | 7 | 0 | 0 | 0 | 0 | 0 | 0 | 0 | 3 | 171 | 0 |
| LB_pharmacophore20 | 1 | 4 | 2 | 0 | 0 | 0 | 0 | 0 | 0 | 0 | 3 | 174 | 0 |
| LB_pharmacophore21 | 1 | 4 | 1 | 0 | 0 | 0 | 0 | 0 | 0 | 0 | 3 | 175 | 0 |

Table S1.(continued next page)

|  | Pharmacophore features | | | | | | | | | | Number of positive and negative hits | | |
| --- | --- | --- | --- | --- | --- | --- | --- | --- | --- | --- | --- | --- | --- |
|  | AR | H | HBA | HBD | PI | NI | AR opt. | H opt. | HBA opt. | HBD opt. | Nbr of agonist ligands found | Accumulated nbr of unique agonist ligands found | Nbr of antagonist ligands found |
| LB_pharmacophore22 | 1 | 1 | 5 | 1 | 0 | 0 | 0 | 0 | 0 | 0 | 2 | 176 | 0 |
| LB_pharmacophore23 | 2 | 3 | 4 | 2 | 0 | 0 | 0 | 0 | 0 | 0 | 2 | 177 | 0 |
| LB_pharmacophore24 | 2 | 1 | 4 | 1 | 0 | 0 | 0 | 0 | 0 | 0 | 2 | 178 | 0 |
| LB_pharmacophore25 | 1 | 1 | 7 | 0 | 0 | 0 | 0 | 0 | 0 | 0 | 2 | 180 | 0 |

Table S1. AR agonist dataset. Pharmacophore features composition of the SBLB agonist selective pharmacophores (AR: aromatic ring, H:hydrophobic, HBA: Hydrogen Bond Acceptor, HBD: Hydrogen Bond Donor, PI: Positive ionizable area, NI: negative ionisable area, opt.: feature set as optional) and number of agonist ligands found with each pharmacophore (“Nbr of agonist ligands found”), number of unique agonist ligands found by combining the pharmacophores (“Accumulated nbr of unique agonist ligands found”) and number of decoys found (“Nbr of antagonist ligands found”)

|  | Pharmacophore features | | | | | | | | | | Number of positive and negative hits | | |
| --- | --- | --- | --- | --- | --- | --- | --- | --- | --- | --- | --- | --- | --- |
|  | AR | H | HBA | HBD | PI | NI | AR opt. | H opt. | HBA opt. | HBD opt. | Nbr of antagonist ligands found | Accumulated nbr of unique antagonist ligands found | Nbr of agonist ligands found |
| LB_pharmacophore1 | 0 | 3 | 2 | 0 | 0 | 0 | 0 | 0 | 0 | 0 | 28 | 28 | 0 |
| LB_pharmacophore2 | 1 | 2 | 6 | 0 | 0 | 0 | 0 | 0 | 0 | 0 | 26 | 54 | 0 |
| LB_pharmacophore3 | 0 | 1 | 2 | 0 | 1 | 0 | 0 | 0 | 0 | 0 | 25 | 66 | 0 |
| LB_pharmacophore4 | 0 | 2 | 3 | 0 | 0 | 0 | 1 | 0 | 0 | 0 | 23 | 73 | 0 |
| LB_pharmacophore5 | 0 | 2 | 2 | 0 | 0 | 0 | 0 | 0 | 0 | 0 | 16 | 84 | 0 |
| LB_pharmacophore6 | 1 | 3 | 0 | 0 | 0 | 0 | 0 | 0 | 0 | 0 | 16 | 85 | 0 |
| LB_pharmacophore7 | 0 | 1 | 2 | 1 | 0 | 0 | 0 | 0 | 0 | 0 | 16 | 92 | 0 |
| LB_pharmacophore8 | 1 | 1 | 2 | 0 | 0 | 0 | 0 | 0 | 0 | 0 | 15 | 103 | 0 |
| LB_pharmacophore9 | 1 | 0 | 2 | 0 | 0 | 0 | 0 | 0 | 0 | 0 | 15 | 105 | 0 |
| LB_pharmacophore10 | 2 | 3 | 4 | 0 | 0 | 0 | 0 | 0 | 0 | 0 | 15 | 120 | 0 |
| LB_pharmacophore11 | 2 | 1 | 4 | 1 | 0 | 0 | 0 | 0 | 0 | 0 | 14 | 128 | 0 |
| LB_pharmacophore12 | 0 | 2 | 3 | 0 | 0 | 0 | 0 | 0 | 0 | 0 | 13 | 129 | 0 |
| LB_pharmacophore13 | 1 | 3 | 3 | 0 | 0 | 0 | 0 | 0 | 0 | 0 | 10 | 136 | 0 |
| LB_pharmacophore14 | 0 | 3 | 2 | 0 | 0 | 0 | 0 | 0 | 0 | 0 | 10 | 138 | 0 |
| LB_pharmacophore15 | 0 | 2 | 2 | 0 | 0 | 0 | 0 | 0 | 0 | 0 | 10 | 144 | 0 |
| LB_pharmacophore16 | 2 | 0 | 3 | 0 | 0 | 0 | 0 | 0 | 0 | 0 | 9 | 146 | 0 |
| LB_pharmacophore17 | 0 | 3 | 2 | 0 | 0 | 0 | 0 | 0 | 0 | 0 | 9 | 147 | 0 |
| LB_pharmacophore18 | 2 | 6 | 0 | 1 | 0 | 0 | 0 | 0 | 0 | 0 | 8 | 155 | 0 |
| LB_pharmacophore19 | 0 | 0 | 4 | 0 | 1 | 0 | 0 | 0 | 0 | 0 | 8 | 157 | 0 |
| LB_pharmacophore20 | 0 | 3 | 2 | 0 | 0 | 0 | 0 | 0 | 0 | 0 | 8 | 158 | 0 |
| LB_pharmacophore21 | 0 | 3 | 3 | 0 | 0 | 0 | 0 | 0 | 0 | 0 | 8 | 163 | 0 |

Table S2. (continued next page)

|  | Pharmacophore features | | | | | | | | | | Number of positive and negative hits | | |
| --- | --- | --- | --- | --- | --- | --- | --- | --- | --- | --- | --- | --- | --- |
|  | AR | H | HBA | HBD | PI | NI | AR opt. | H opt. | HBA opt. | HBD opt. | Nbr of antagonist ligands found | Accumulated nbr of unique antagonist ligands found | Nbr of agonist ligands found |
| LB_pharmacophore22 | 0 | 2 | 4 | 0 | 0 | 0 | 0 | 0 | 0 | 0 | 8 | 164 | 0 |
| LB_pharmacophore23 | 1 | 1 | 5 | 0 | 0 | 0 | 0 | 0 | 0 | 0 | 7 | 171 | 0 |
| LB_pharmacophore24 | 2 | 1 | 4 | 0 | 0 | 0 | 0 | 0 | 0 | 0 | 7 | 175 | 0 |
| LB_pharmacophore25 | 1 | 3 | 3 | 0 | 0 | 0 | 0 | 0 | 0 | 0 | 6 | 180 | 0 |
| LB_pharmacophore26 | 0 | 2 | 4 | 2 | 0 | 0 | 0 | 0 | 0 | 0 | 6 | 185 | 0 |
| LB_pharmacophore27 | 1 | 0 | 5 | 0 | 0 | 0 | 0 | 0 | 0 | 0 | 6 | 188 | 0 |
| LB_pharmacophore28 | 1 | 1 | 2 | 0 | 0 | 0 | 0 | 1 | 1 | 0 | 6 | 192 | 0 |
| LB_pharmacophore29 | 0 | 2 | 4 | 2 | 0 | 0 | 0 | 0 | 0 | 0 | 5 | 193 | 0 |
| LB_pharmacophore30 | 0 | 1 | 5 | 1 | 0 | 0 | 0 | 0 | 0 | 0 | 5 | 194 | 0 |
| LB_pharmacophore31 | 0 | 1 | 2 | 1 | 0 | 0 | 0 | 0 | 0 | 0 | 4 | 197 | 0 |
| LB_pharmacophore32 | 1 | 3 | 1 | 0 | 0 | 0 | 0 | 0 | 0 | 0 | 4 | 200 | 0 |
| LB_pharmacophore33 | 2 | 2 | 4 | 0 | 0 | 0 | 0 | 0 | 0 | 0 | 4 | 201 | 0 |
| LB_pharmacophore34 | 1 | 2 | 5 | 0 | 0 | 0 | 0 | 0 | 0 | 0 | 4 | 204 | 0 |
| LB_pharmacophore35 | 1 | 1 | 2 | 1 | 0 | 0 | 0 | 0 | 0 | 0 | 4 | 205 | 0 |
| LB_pharmacophore36 | 2 | 3 | 2 | 0 | 0 | 0 | 0 | 0 | 0 | 0 | 4 | 207 | 0 |
| LB_pharmacophore37 | 0 | 4 | 2 | 0 | 0 | 0 | 0 | 0 | 0 | 0 | 3 | 208 | 0 |
| LB_pharmacophore38 | 2 | 4 | 4 | 0 | 0 | 0 | 0 | 0 | 0 | 0 | 3 | 209 | 0 |
| LB_pharmacophore39 | 2 | 2 | 2 | 0 | 0 | 0 | 0 | 0 | 0 | 0 | 3 | 211 | 0 |
| LB_pharmacophore40 | 1 | 5 | 1 | 0 | 0 | 0 | 0 | 0 | 0 | 0 | 2 | 213 | 0 |
| LB_pharmacophore41 | 2 | 2 | 1 | 0 | 0 | 0 | 0 | 0 | 0 | 0 | 2 | 215 | 0 |
| LB_pharmacophore42 | 1 | 4 | 2 | 0 | 0 | 0 | 0 | 0 | 0 | 0 | 2 | 216 | 0 |
| LB_pharmacophore43 | 0 | 1 | 2 | 2 | 0 | 0 | 0 | 0 | 0 | 0 | 2 | 218 | 0 |
| LB_pharmacophore44 | 0 | 1 | 1 | 2 | 0 | 0 | 0 | 0 | 0 | 0 | 2 | 220 | 0 |
| LB_pharmacophore45 | 1 | 2 | 2 | 1 | 0 | 0 | 0 | 0 | 0 | 0 | 2 | 221 | 0 |

Table S2. (continued next page)

|  | Pharmacophore features | | | | | | | | | | Number of positive and negative hits | | |
| --- | --- | --- | --- | --- | --- | --- | --- | --- | --- | --- | --- | --- | --- |
|  | AR | H | HBA | HBD | PI | NI | AR opt. | H opt. | HBA opt. | HBD opt. | Nbr of antagonist ligands found | Accumulated nbr of unique antagonist ligands found | Nbr of agonist ligands found |
| LB_pharmacophore46 | 0 | 2 | 7 | 1 | 0 | 0 | 0 | 0 | 0 | 0 | 1 | 222 | 0 |
| LB_pharmacophore47 | 0 | 3 | 1 | 2 | 0 | 0 | 0 | 0 | 0 | 0 | 1 | 223 | 0 |
| LB_pharmacophore48 | 2 | 2 | 2 | 1 | 0 | 0 | 0 | 0 | 0 | 0 | 1 | 224 | 0 |

Table S2. AR antagonist dataset. Pharmacophore features composition of the SBLB agonist selective pharmacophores (AR: aromatic ring, H:hydrophobic, HBA: Hydrogen Bond Acceptor, HBD: Hydrogen Bond Donor, PI: Positive ionizable area, NI: negative ionisable area, opt.: feature set as optional) and number of antagonist ligands found with each pharmacophore (“Nbr of antagonist ligands found”), number of unique antagonist ligands found by combining the pharmacophores (“Accumulated nbr of unique antagonist ligands found”) and number of decoys found (“Nbr of agonist ligands found”)

|  | Pharmacophore features | | | | | | | | | | Number of positive and negative hits | | |
| --- | --- | --- | --- | --- | --- | --- | --- | --- | --- | --- | --- | --- | --- |
|  | AR | H | HBA | HBD | PI | NI | AR opt. | H opt. | HBA opt. | HBD opt. | Nbr of agonist ligands found | Accumulated nbr of unique agonist ligands found | Nbr of antagonist ligands found |
| LB_pharmacophore1 | 1 | 3 | 0 | 0 | 0 | 0 | 0 | 0 | 0 | 0 | 15 | 15 | 0 |
| LB_pharmacophore2 | 0 | 1 | 1 | 1 | 0 | 0 | 0 | 0 | 0 | 0 | 14 | 23 | 0 |
| LB_pharmacophore3 | 0 | 2 | 1 | 0 | 0 | 0 | 0 | 0 | 0 | 0 | 13 | 27 | 0 |
| LB_pharmacophore4 | 0 | 3 | 0 | 0 | 0 | 0 | 0 | 0 | 0 | 0 | 12 | 30 | 0 |
| LB_pharmacophore5 | 1 | 1 | 2 | 0 | 0 | 0 | 0 | 0 | 0 | 0 | 11 | 33 | 0 |

Table S3. CAR agonist dataset. Pharmacophore features composition of the SBLB agonist selective pharmacophores (AR: aromatic ring, H:hydrophobic, HBA: Hydrogen Bond Acceptor, HBD: Hydrogen Bond Donor, PI: Positive ionizable area, NI: negative ionisable area, opt.: feature set as optional) and number of agonist ligands found with each pharmacophore (“Nbr of agonist ligands found”), number of unique agonist ligands found by combining the pharmacophores (“Accumulated nbr of unique agonist ligands found”) and number of decoys found (“Nbr of antagonist ligands found”)

|  | Pharmacophore features | | | | | | | | | | Number of positive and negative hits | | |
| --- | --- | --- | --- | --- | --- | --- | --- | --- | --- | --- | --- | --- | --- |
|  | AR | H | HBA | HBD | PI | NI | AR opt. | H opt. | HBA opt. | HBD opt. | Nbr of antagonist ligands found | Accumulated nbr of unique antagonist ligands found | Nbr of agonist ligands found |
| LB_pharmacophore1 | 2 | 3 | 0 | 0 | 0 | 0 | 0 | 0 | 0 | 0 | 2 | 2 | 0 |

Table S4. CAR antagonist dataset. Pharmacophore features composition of the SBLB agonist selective pharmacophores (AR: aromatic ring, H:hydrophobic, HBA: Hydrogen Bond Acceptor, HBD: Hydrogen Bond Donor, PI: Positive ionizable area, NI: negative ionisable area, opt.: feature set as optional) and number of antagonist ligands found with each pharmacophore (“Nbr of antagonist ligands found”), number of unique antagonist ligands found by combining the pharmacophores (“Accumulated nbr of unique antagonist ligands found”) and number of decoys found (“Nbr of agonist ligands found”)

|  | Pharmacophore features | | | | | | | | | | Number of positive and negative hits | | |
| --- | --- | --- | --- | --- | --- | --- | --- | --- | --- | --- | --- | --- | --- |
|  | AR | H | HBA | HBD | PI | NI | AR opt. | H opt. | HBA opt. | HBD opt. | Nbr of agonist ligands found | Accumulated nbr of unique agonist ligands found | Nbr of antagonist ligands found |
| LB_pharmacophore1 | 0 | 1 | 2 | 2 | 0 | 0 | 0 | 0 | 0 | 0 | 165 | 165 | 0 |
| LB_pharmacophore2 | 1 | 2 | 2 | 1 | 0 | 0 | 0 | 2 | 0 | 0 | 158 | 195 | 0 |
| LB_pharmacophore3 | 1 | 2 | 2 | 2 | 0 | 0 | 0 | 0 | 0 | 0 | 117 | 235 | 0 |
| SB_pharmacophore4 | 0 | 2 | 1 | 1 | 0 | 0 | 0 | 0 | 0 | 0 | 107 | 267 | 0 |
| SB_pharmacophore5 | 0 | 2 | 3 | 0 | 0 | 0 | 0 | 0 | 0 | 0 | 102 | 279 | 0 |
| LB_pharmacophore6 | 0 | 1 | 2 | 1 | 0 | 0 | 0 | 0 | 0 | 0 | 95 | 292 | 0 |
| LB_pharmacophore7 | 1 | 1 | 2 | 1 | 0 | 0 | 0 | 0 | 0 | 0 | 93 | 296 | 0 |
| LB_pharmacophore8 | 0 | 1 | 2 | 2 | 0 | 0 | 0 | 0 | 0 | 0 | 76 | 299 | 0 |
| SB_pharmacophore9 | 0 | 1 | 3 | 1 | 0 | 0 | 0 | 0 | 0 | 0 | 75 | 303 | 0 |
| LB_pharmacophore10 | 0 | 1 | 2 | 1 | 0 | 0 | 0 | 0 | 0 | 0 | 69 | 317 | 0 |
| LB_pharmacophore11 | 0 | 1 | 2 | 2 | 0 | 0 | 0 | 0 | 0 | 0 | 55 | 323 | 0 |
| LB_pharmacophore12 | 2 | 2 | 2 | 2 | 0 | 0 | 0 | 0 | 0 | 0 | 55 | 337 | 0 |
| LB_pharmacophore13 | 1 | 1 | 2 | 1 | 0 | 0 | 0 | 0 | 0 | 0 | 51 | 345 | 0 |
| LB_pharmacophore14 | 2 | 0 | 2 | 1 | 0 | 0 | 0 | 0 | 0 | 0 | 43 | 348 | 0 |
| LB_pharmacophore15 | 1 | 1 | 2 | 1 | 0 | 0 | 0 | 0 | 0 | 0 | 42 | 351 | 0 |
| SB_pharmacophore16 | 0 | 2 | 0 | 2 | 0 | 0 | 0 | 0 | 0 | 0 | 36 | 359 | 0 |
| SB_pharmacophore17 | 0 | 2 | 2 | 1 | 0 | 0 | 0 | 0 | 0 | 0 | 34 | 361 | 0 |
| LB_pharmacophore18 | 1 | 2 | 1 | 1 | 0 | 0 | 0 | 2 | 0 | 1 | 34 | 364 | 0 |
| LB_pharmacophore19 | 1 | 1 | 2 | 0 | 0 | 0 | 0 | 0 | 0 | 0 | 33 | 369 | 0 |
| LB_pharmacophore20 | 0 | 3 | 1 | 1 | 0 | 0 | 0 | 0 | 0 | 0 | 30 | 371 | 0 |
| LB_pharmacophore21 | 1 | 1 | 3 | 1 | 0 | 0 | 0 | 0 | 0 | 0 | 30 | 373 | 0 |
| LB_pharmacophore22 | 2 | 1 | 2 | 0 | 0 | 0 | 0 | 0 | 0 | 0 | 26 | 375 | 0 |

Table S5. (continued next page)

|  | Pharmacophore features | | | | | | | | | | Number of positive and negative hits | | |
| --- | --- | --- | --- | --- | --- | --- | --- | --- | --- | --- | --- | --- | --- |
|  | AR | H | HBA | HBD | PI | NI | AR opt. | H opt. | HBA opt. | HBD opt. | Nbr of agonist ligands found | Accumulated nbr of unique agonist ligands found | Nbr of antagonist ligands found |
| LB_pharmacophore23 | 2 | 1 | 1 | 0 | 0 | 0 | 0 | 0 | 0 | 0 | 23 | 378 | 0 |
| LB_pharmacophore24 | 0 | 0 | 3 | 1 | 0 | 0 | 0 | 0 | 0 | 0 | 21 | 380 | 0 |
| LB_pharmacophore25 | 2 | 2 | 1 | 0 | 0 | 0 | 0 | 0 | 0 | 0 | 20 | 381 | 0 |
| LB_pharmacophore26 | 1 | 0 | 1 | 1 | 0 | 0 | 0 | 0 | 0 | 0 | 20 | 391 | 0 |
| LB_pharmacophore27 | 1 | 0 | 3 | 2 | 0 | 0 | 0 | 0 | 0 | 0 | 19 | 403 | 0 |
| LB_pharmacophore28 | 1 | 2 | 0 | 0 | 0 | 0 | 0 | 0 | 2 | 1 | 16 | 407 | 0 |
| LB_pharmacophore29 | 2 | 2 | 1 | 1 | 0 | 0 | 0 | 0 | 0 | 0 | 15 | 409 | 0 |
| LB_pharmacophore30 | 2 | 0 | 2 | 1 | 1 | 0 | 0 | 3 | 0 | 0 | 15 | 416 | 0 |
| LB_pharmacophore31 | 0 | 2 | 2 | 2 | 0 | 0 | 0 | 0 | 0 | 0 | 15 | 417 | 0 |
| LB_pharmacophore32 | 0 | 2 | 2 | 1 | 0 | 0 | 0 | 0 | 0 | 0 | 13 | 418 | 0 |
| LB_pharmacophore33 | 2 | 2 | 1 | 1 | 0 | 0 | 0 | 0 | 0 | 0 | 12 | 420 | 0 |
| LB_pharmacophore34 | 1 | 2 | 3 | 2 | 0 | 0 | 0 | 0 | 0 | 0 | 11 | 421 | 0 |
| LB_pharmacophore35 | 0 | 1 | 3 | 1 | 0 | 0 | 0 | 0 | 0 | 0 | 10 | 422 | 0 |
| LB_pharmacophore36 | 1 | 2 | 1 | 1 | 0 | 0 | 0 | 0 | 0 | 0 | 9 | 425 | 0 |
| LB_pharmacophore37 | 0 | 3 | 1 | 0 | 0 | 0 | 0 | 0 | 0 | 0 | 5 | 428 | 0 |
| LB_pharmacophore38 | 3 | 4 | 2 | 1 | 0 | 0 | 0 | 0 | 0 | 0 | 3 | 429 | 0 |
| LB_pharmacophore39 | 0 | 0 | 4 | 3 | 0 | 0 | 0 | 0 | 0 | 0 | 1 | 430 | 0 |

Table S5. ER_alpha agonist dataset. Pharmacophore features composition of the SBLB agonist selective pharmacophores (AR: aromatic ring, H:hydrophobic, HBA: Hydrogen Bond Acceptor, HBD: Hydrogen Bond Donor, PI: Positive ionizable area, NI: negative ionisable area, opt.: feature set as optional) and number of agonist ligands found with each pharmacophore (“Nbr of agonist ligands found”), number of unique agonist ligands found by combining the pharmacophores (“Accumulated nbr of unique agonist ligands found”) and number of decoys found (“Nbr of antagonist ligands found”)

|  | Pharmacophore features | | | | | | | | | | Number of positive and negative hits | | |
| --- | --- | --- | --- | --- | --- | --- | --- | --- | --- | --- | --- | --- | --- |
|  | AR | H | HBA | HBD | PI | NI | AR opt. | H opt. | HBA opt. | HBD opt. | Nbr of antagonist ligands found | Accumulated nbr of unique antagonist ligands found | Nbr of agonist ligands found |
| SB_pharmacophore1 | 0 | 3 | 2 | 1 | 1 | 0 | 0 | 0 | 0 | 0 | 34 | 34 | 0 |
| LB_pharmacophore2 | 0 | 2 | 5 | 1 | 0 | 0 | 0 | 0 | 0 | 0 | 27 | 61 | 0 |
| SB_pharmacophore3 | 0 | 3 | 0 | 0 | 1 | 0 | 0 | 0 | 0 | 0 | 19 | 70 | 0 |
| SB_pharmacophore4 | 0 | 2 | 3 | 1 | 1 | 0 | 0 | 0 | 0 | 0 | 19 | 72 | 0 |
| LB_pharmacophore5 | 1 | 2 | 1 | 1 | 1 | 0 | 0 | 0 | 0 | 0 | 18 | 77 | 0 |
| SB_pharmacophore6 | 0 | 2 | 0 | 2 | 1 | 0 | 0 | 0 | 0 | 0 | 15 | 80 | 0 |
| SB_pharmacophore7 | 0 | 2 | 0 | 1 | 1 | 0 | 0 | 0 | 0 | 0 | 15 | 81 | 0 |
| LB_pharmacophore8 | 1 | 2 | 3 | 1 | 0 | 0 | 0 | 0 | 0 | 0 | 14 | 95 | 0 |
| LB_pharmacophore9 | 3 | 0 | 1 | 0 | 0 | 0 | 0 | 0 | 0 | 0 | 13 | 101 | 0 |
| LB_pharmacophore10 | 1 | 2 | 2 | 1 | 0 | 0 | 0 | 0 | 0 | 1 | 9 | 108 | 0 |
| LB_pharmacophore11 | 2 | 2 | 2 | 0 | 0 | 0 | 0 | 0 | 0 | 0 | 8 | 112 | 0 |
| LB_pharmacophore12 | 2 | 3 | 1 | 0 | 0 | 0 | 0 | 0 | 0 | 0 | 6 | 114 | 0 |
| LB_pharmacophore13 | 2 | 2 | 2 | 1 | 0 | 0 | 0 | 1 | 0 | 0 | 5 | 116 | 0 |
| LB_pharmacophore14 | 3 | 3 | 2 | 2 | 0 | 0 | 0 | 0 | 0 | 0 | 4 | 120 | 0 |
| SB_pharmacophore15 | 0 | 4 | 2 | 0 | 0 | 0 | 0 | 0 | 0 | 0 | 3 | 123 | 0 |
| LB_pharmacophore16 | 2 | 3 | 3 | 1 | 0 | 0 | 0 | 0 | 0 | 0 | 3 | 126 | 0 |
| LB_pharmacophore17 | 4 | 4 | 2 | 2 | 0 | 0 | 0 | 0 | 0 | 0 | 3 | 127 | 0 |
| LB_pharmacophore18 | 2 | 3 | 4 | 1 | 0 | 0 | 0 | 0 | 0 | 0 | 3 | 129 | 0 |
| LB_pharmacophore19 | 1 | 2 | 3 | 2 | 0 | 0 | 0 | 0 | 0 | 0 | 3 | 130 | 0 |
| LB_pharmacophore20 | 3 | 2 | 3 | 1 | 0 | 0 | 0 | 1 | 0 | 0 | 2 | 131 | 0 |
| LB_pharmacophore21 | 3 | 4 | 2 | 2 | 0 | 0 | 0 | 0 | 0 | 0 | 2 | 132 | 0 |

Table S6. (continued next page)

|  | Pharmacophore features | | | | | | | | | | Number of positive and negative hits | | |
| --- | --- | --- | --- | --- | --- | --- | --- | --- | --- | --- | --- | --- | --- |
|  | AR | H | HBA | HBD | PI | NI | AR opt. | H opt. | HBA opt. | HBD opt. | Nbr of antagonist ligands found | Accumulated nbr of unique antagonist ligands found | Nbr of agonist ligands found |
| LB_pharmacophore22 | 2 | 3 | 2 | 1 | 0 | 0 | 0 | 0 | 0 | 0 | 2 | 134 | 0 |
| LB_pharmacophore23 | 1 | 3 | 2 | 1 | 0 | 0 | 0 | 0 | 0 | 0 | 1 | 135 | 0 |

Table S6. ER_alpha antagonist dataset. Pharmacophore features composition of the SBLB agonist selective pharmacophores (AR: aromatic ring, H:hydrophobic, HBA: Hydrogen Bond Acceptor, HBD: Hydrogen Bond Donor, PI: Positive ionizable area, NI: negative ionisable area, opt.: feature set as optional) and number of antagonist ligands found with each pharmacophore (“Nbr of antagonist ligands found”), number of unique antagonist ligands found by combining the pharmacophores (“Accumulated nbr of unique antagonist ligands found”) and number of decoys found (“Nbr of agonist ligands found”)

|  | Pharmacophore features | | | | | | | | | | Number of positive and negative hits | | |
| --- | --- | --- | --- | --- | --- | --- | --- | --- | --- | --- | --- | --- | --- |
|  | AR | H | HBA | HBD | PI | NI | AR opt. | H opt. | HBA opt. | HBD opt. | Nbr of agonist ligands found | Accumulated nbr of unique agonist ligands found | Nbr of antagonist ligands found |
| LB_pharmacophore1 | 0 | 1 | 2 | 2 | 0 | 0 | 0 | 0 | 0 | 0 | 163 | 163 | 0 |
| LB_pharmacophore2 | 0 | 1 | 2 | 2 | 0 | 0 | 0 | 0 | 0 | 0 | 159 | 214 | 0 |
| SB_pharmacophore3 | 0 | 1 | 1 | 2 | 0 | 0 | 0 | 0 | 0 | 0 | 104 | 233 | 0 |
| LB_pharmacophore4 | 0 | 2 | 1 | 1 | 0 | 0 | 0 | 0 | 0 | 0 | 89 | 244 | 0 |
| LB_pharmacophore5 | 1 | 2 | 1 | 1 | 0 | 0 | 0 | 0 | 0 | 0 | 82 | 262 | 0 |
| LB_pharmacophore6 | 1 | 1 | 2 | 1 | 0 | 0 | 0 | 0 | 0 | 0 | 77 | 290 | 0 |
| LB_pharmacophore7 | 1 | 1 | 2 | 0 | 0 | 0 | 0 | 0 | 0 | 0 | 76 | 294 | 0 |
| LB_pharmacophore8 | 1 | 1 | 1 | 2 | 0 | 0 | 0 | 0 | 0 | 0 | 70 | 298 | 0 |
| LB_pharmacophore9 | 1 | 1 | 1 | 1 | 0 | 0 | 0 | 0 | 0 | 0 | 67 | 309 | 0 |
| LB_pharmacophore10 | 0 | 2 | 3 | 2 | 0 | 0 | 0 | 0 | 0 | 0 | 61 | 311 | 0 |
| SB_pharmacophore11 | 1 | 1 | 2 | 0 | 0 | 0 | 0 | 0 | 0 | 0 | 60 | 317 | 0 |
| LB_pharmacophore12 | 1 | 2 | 1 | 2 | 0 | 0 | 1 | 0 | 2 | 1 | 51 | 321 | 0 |
| LB_pharmacophore13 | 1 | 1 | 2 | 2 | 0 | 0 | 0 | 0 | 0 | 0 | 51 | 322 | 0 |
| LB_pharmacophore14 | 1 | 1 | 1 | 1 | 0 | 0 | 0 | 0 | 0 | 0 | 46 | 325 | 0 |
| LB_pharmacophore15 | 1 | 1 | 2 | 1 | 0 | 0 | 0 | 0 | 0 | 0 | 38 | 331 | 0 |
| LB_pharmacophore16 | 1 | 2 | 2 | 0 | 0 | 0 | 0 | 0 | 0 | 0 | 35 | 342 | 0 |
| SB_pharmacophore17 | 0 | 2 | 1 | 1 | 0 | 0 | 0 | 0 | 0 | 0 | 34 | 345 | 0 |
| LB_pharmacophore18 | 1 | 1 | 2 | 0 | 0 | 0 | 0 | 0 | 0 | 0 | 30 | 347 | 0 |
| LB_pharmacophore19 | 1 | 1 | 3 | 0 | 0 | 0 | 0 | 0 | 0 | 0 | 25 | 351 | 0 |
| SB_pharmacophore20 | 0 | 2 | 2 | 2 | 0 | 0 | 0 | 0 | 0 | 0 | 24 | 354 | 0 |
| LB_pharmacophore21 | 1 | 1 | 2 | 0 | 0 | 0 | 0 | 0 | 0 | 0 | 22 | 355 | 0 |
| LB_pharmacophore22 | 0 | 1 | 2 | 2 | 0 | 0 | 0 | 0 | 0 | 0 | 22 | 360 | 0 |

Table S7. (continued next page)

|  | Pharmacophore features | | | | | | | | | | Number of positive and negative hits | | |
| --- | --- | --- | --- | --- | --- | --- | --- | --- | --- | --- | --- | --- | --- |
|  | AR | H | HBA | HBD | PI | NI | AR opt. | H opt. | HBA opt. | HBD opt. | Nbr of agonist ligands found | Accumulated nbr of unique agonist ligands found | Nbr of antagonist ligands found |
| SB_pharmacophore23 | 0 | 2 | 2 | 1 | 0 | 0 | 0 | 0 | 0 | 0 | 21 | 361 | 0 |
| LB_pharmacophore24 | 1 | 1 | 2 | 2 | 0 | 0 | 0 | 0 | 0 | 0 | 20 | 362 | 0 |
| LB_pharmacophore25 | 2 | 1 | 2 | 1 | 0 | 0 | 0 | 0 | 0 | 0 | 20 | 367 | 0 |
| SB_pharmacophore26 | 0 | 1 | 2 | 2 | 0 | 0 | 0 | 0 | 0 | 0 | 17 | 369 | 0 |
| LB_pharmacophore27 | 2 | 2 | 1 | 1 | 0 | 0 | 0 | 0 | 1 | 0 | 13 | 374 | 0 |
| LB_pharmacophore28 | 0 | 3 | 1 | 1 | 0 | 0 | 0 | 0 | 0 | 0 | 10 | 373 | 0 |
| SB_pharmacophore29 | 0 | 3 | 1 | 0 | 0 | 0 | 0 | 0 | 0 | 0 | 9 | 375 | 0 |
| LB_pharmacophore30 | 0 | 4 | 1 | 1 | 0 | 0 | 0 | 0 | 0 | 0 | 8 | 379 | 0 |
| SB_pharmacophore31 | 0 | 2 | 2 | 3 | 0 | 0 | 0 | 0 | 0 | 0 | 6 | 380 | 0 |
| LB_pharmacophore32 | 3 | 3 | 2 | 1 | 0 | 0 | 0 | 0 | 0 | 1 | 4 | 382 | 0 |
| LB_pharmacophore33 | 0 | 1 | 2 | 1 | 1 | 0 | 0 | 0 | 0 | 0 | 1 | 383 | 0 |

Table S7. ER_beta agonist dataset. Pharmacophore features composition of the SBLB agonist selective pharmacophores (AR: aromatic ring, H:hydrophobic, HBA: Hydrogen Bond Acceptor, HBD: Hydrogen Bond Donor, PI: Positive ionizable area, NI: negative ionisable area, opt.: feature set as optional) and number of agonist ligands found with each pharmacophore (“Nbr of agonist ligands found”), number of unique agonist ligands found by combining the pharmacophores (“Accumulated nbr of unique agonist ligands found”) and number of decoys found (“Nbr of antagonist ligands found”)

|  | Pharmacophore features | | | | | | | | | | Number of positive and negative hits | | |
| --- | --- | --- | --- | --- | --- | --- | --- | --- | --- | --- | --- | --- | --- |
|  | AR | H | HBA | HBD | PI | NI | AR opt. | H opt. | HBA opt. | HBD opt. | Nbr of antagonist ligands found | Accumulated nbr of unique antagonist ligands found | Nbr of agonist ligands found |
| LB_pharmacophore1 | 1 | 3 | 2 | 1 | 0 | 0 | 0 | 0 | 0 | 0 | 13 | 13 | 0 |
| LB_pharmacophore2 | 2 | 3 | 2 | 1 | 0 | 0 | 0 | 0 | 0 | 0 | 10 | 18 | 0 |
| LB_pharmacophore3 | 2 | 2 | 1 | 1 | 0 | 0 | 0 | 0 | 0 | 0 | 9 | 27 | 0 |
| LB_pharmacophore4 | 1 | 3 | 2 | 1 | 0 | 0 | 0 | 0 | 0 | 0 | 9 | 31 | 0 |
| LB_pharmacophore5 | 1 | 3 | 3 | 2 | 0 | 0 | 0 | 1 | 0 | 0 | 8 | 39 | 0 |
| LB_pharmacophore6 | 0 | 3 | 2 | 1 | 1 | 0 | 0 | 0 | 0 | 0 | 8 | 43 | 0 |
| LB_pharmacophore7 | 1 | 2 | 3 | 2 | 0 | 0 | 0 | 0 | 0 | 0 | 7 | 44 | 0 |
| SB_pharmacophore8 | 0 | 1 | 0 | 1 | 1 | 0 | 0 | 0 | 0 | 0 | 5 | 47 | 0 |
| LB_pharmacophore9 | 0 | 3 | 3 | 1 | 0 | 0 | 0 | 0 | 0 | 0 | 5 | 52 | 0 |
| LB_pharmacophore10 | 2 | 3 | 3 | 1 | 0 | 0 | 1 | 0 | 2 | 0 | 4 | 53 | 0 |
| LB_pharmacophore11 | 0 | 2 | 2 | 1 | 1 | 0 | 0 | 0 | 0 | 0 | 4 | 56 | 0 |
| LB_pharmacophore12 | 1 | 1 | 2 | 0 | 0 | 0 | 0 | 0 | 0 | 0 | 3 | 58 | 0 |
| LB_pharmacophore13 | 0 | 3 | 2 | 1 | 0 | 0 | 0 | 0 | 0 | 0 | 3 | 61 | 0 |
| LB_pharmacophore14 | 2 | 5 | 9 | 0 | 0 | 0 | 0 | 0 | 0 | 0 | 3 | 62 | 0 |
| LB_pharmacophore15 | 1 | 3 | 2 | 2 | 0 | 0 | 0 | 0 | 0 | 0 | 2 | 64 | 0 |
| LB_pharmacophore16 | 0 | 2 | 3 | 1 | 0 | 0 | 0 | 0 | 0 | 0 | 2 | 66 | 0 |
| LB_pharmacophore17 | 3 | 3 | 4 | 1 | 0 | 0 | 0 | 0 | 0 | 0 | 1 | 67 | 0 |
| LB_pharmacophore18 | 2 | 4 | 2 | 2 | 0 | 0 | 0 | 0 | 0 | 0 | 1 | 68 | 0 |

Table S8. ER_beta antagonist dataset. Pharmacophore features composition of the SBLB agonist selective pharmacophores (AR: aromatic ring, H:hydrophobic, HBA: Hydrogen Bond Acceptor, HBD: Hydrogen Bond Donor, PI: Positive ionizable area, NI: negative ionisable area, opt.: feature set as optional) and number of antagonist ligands found with each pharmacophore (“Nbr of antagonist ligands found”), number of unique antagonist ligands found by combining the pharmacophores (“Accumulated nbr of unique antagonist ligands found”) and number of decoys found (“Nbr of agonist ligands found”)

|  | Pharmacophore features | | | | | | | | | | Number of positive and negative hits | | |
| --- | --- | --- | --- | --- | --- | --- | --- | --- | --- | --- | --- | --- | --- |
|  | AR | H | HBA | HBD | PI | NI | AR opt. | H opt. | HBA opt. | HBD opt. | Nbr of agonist ligands found | Accumulated nbr of unique agonist ligands found | Nbr of antagonist ligands found |
| LB_pharmacophore1 | 2 | 2 | 2 | 0 | 0 | 0 | 0 | 0 | 0 | 0 | 9 | 9 | 0 |
| LB_pharmacophore2 | 3 | 2 | 1 | 0 | 0 | 0 | 0 | 0 | 0 | 0 | 7 | 10 | 0 |
| LB_pharmacophore3 | 3 | 1 | 3 | 1 | 0 | 0 | 0 | 0 | 0 | 0 | 3 | 13 | 0 |

Table S9. ERR_alpha agonist dataset. Pharmacophore features composition of the SBLB agonist selective pharmacophores (AR: aromatic ring, H:hydrophobic, HBA: Hydrogen Bond Acceptor, HBD: Hydrogen Bond Donor, PI: Positive ionizable area, NI: negative ionisable area, opt.: feature set as optional) and number of agonist ligands found with each pharmacophore (“Nbr of agonist ligands found”), number of unique agonist ligands found by combining the pharmacophores (“Accumulated nbr of unique agonist ligands found”) and number of decoys found (“Nbr of antagonist ligands found”)

|  | Pharmacophore features | | | | | | | | | | Number of positive and negative hits | | |
| --- | --- | --- | --- | --- | --- | --- | --- | --- | --- | --- | --- | --- | --- |
|  | AR | H | HBA | HBD | PI | NI | AR opt. | H opt. | HBA opt. | HBD opt. | Nbr of antagonist ligands found | Accumulated nbr of unique antagonist ligands found | Nbr of agonist ligands found |
| LB_pharmacophore1 | 0 | 4 | 0 | 0 | 0 | 0 | 0 | 0 | 0 | 0 | 3 | 3 | 0 |

Table S10. ERR_alpha antagonist dataset. Pharmacophore features composition of the SBLB agonist selective pharmacophores (AR: aromatic ring, H:hydrophobic, HBA: Hydrogen Bond Acceptor, HBD: Hydrogen Bond Donor, PI: Positive ionizable area, NI: negative ionisable area, opt.: feature set as optional) and number of antagonist ligands found with each pharmacophore (“Nbr of antagonist ligands found”), number of unique antagonist ligands found by combining the pharmacophores (“Accumulated nbr of unique antagonist ligands found”) and number of decoys found (“Nbr of agonist ligands found”)

|  | Pharmacophore features | | | | | | | | | | Number of positive and negative hits | | |
| --- | --- | --- | --- | --- | --- | --- | --- | --- | --- | --- | --- | --- | --- |
|  | AR | H | HBA | HBD | PI | NI | AR opt. | H opt. | HBA opt. | HBD opt. | Nbr of agonist ligands found | Accumulated nbr of unique agonist ligands found | Nbr of antagonist ligands found |
| LB_pharmacophore1 | 0 | 3 | 1 | 0 | 0 | 0 | 0 | 0 | 0 | 0 | 216 | 216 | 0 |
| SB_pharmacophore2 | 0 | 3 | 1 | 0 | 0 | 0 | 0 | 0 | 0 | 0 | 100 | 226 | 0 |
| SB_pharmacophore3 | 0 | 2 | 1 | 0 | 0 | 0 | 0 | 0 | 0 | 0 | 81 | 229 | 0 |
| SB_pharmacophore4 | 0 | 3 | 1 | 0 | 0 | 1 | 0 | 0 | 0 | 0 | 67 | 230 | 0 |
| LB_pharmacophore5 | 0 | 3 | 2 | 0 | 0 | 0 | 0 | 0 | 0 | 0 | 54 | 245 | 0 |
| LB_pharmacophore6 | 1 | 2 | 1 | 0 | 0 | 0 | 0 | 0 | 0 | 0 | 35 | 247 | 0 |
| LB_pharmacophore7 | 0 | 2 | 2 | 0 | 0 | 0 | 0 | 1 | 0 | 0 | 34 | 252 | 0 |
| LB_pharmacophore8 | 0 | 0 | 3 | 2 | 0 | 0 | 0 | 0 | 0 | 0 | 33 | 283 | 0 |
| LB_pharmacophore9 | 0 | 1 | 3 | 0 | 0 | 0 | 0 | 0 | 0 | 0 | 33 | 290 | 0 |
| LB_pharmacophore10 | 0 | 0 | 3 | 1 | 0 | 0 | 0 | 0 | 0 | 0 | 28 | 291 | 0 |
| LB_pharmacophore11 | 0 | 3 | 1 | 0 | 0 | 0 | 0 | 0 | 0 | 0 | 28 | 293 | 0 |
| SB_pharmacophore12 | 0 | 4 | 1 | 0 | 0 | 1 | 0 | 0 | 0 | 0 | 27 | 294 | 0 |
| LB_pharmacophore13 | 0 | 3 | 1 | 0 | 0 | 0 | 0 | 1 | 0 | 0 | 27 | 298 | 0 |
| LB_pharmacophore14 | 0 | 3 | 2 | 2 | 0 | 0 | 0 | 0 | 0 | 0 | 27 | 299 | 0 |
| LB_pharmacophore15 | 1 | 2 | 2 | 0 | 0 | 0 | 0 | 0 | 0 | 0 | 26 | 302 | 0 |
| LB_pharmacophore16 | 3 | 3 | 3 | 0 | 0 | 0 | 0 | 0 | 0 | 0 | 23 | 312 | 0 |
| LB_pharmacophore17 | 2 | 2 | 5 | 1 | 0 | 0 | 0 | 0 | 0 | 0 | 22 | 313 | 0 |
| LB_pharmacophore18 | 0 | 1 | 2 | 1 | 1 | 0 | 0 | 0 | 0 | 0 | 21 | 315 | 0 |
| LB_pharmacophore19 | 0 | 3 | 1 | 0 | 0 | 0 | 0 | 0 | 0 | 0 | 11 | 316 | 0 |
| LB_pharmacophore20 | 0 | 1 | 2 | 1 | 0 | 0 | 0 | 0 | 0 | 0 | 10 | 317 | 0 |

Table S11. (continued next page)

|  | Pharmacophore features | | | | | | | | | | Number of positive and negative hits | | |
| --- | --- | --- | --- | --- | --- | --- | --- | --- | --- | --- | --- | --- | --- |
|  | AR | H | HBA | HBD | PI | NI | AR opt. | H opt. | HBA opt. | HBD opt. | Nbr of agonist ligands found | Accumulated nbr of unique agonist ligands found | Nbr of antagonist ligands found |
| LB_pharmacophore21 | 0 | 4 | 2 | 0 | 0 | 0 | 0 | 0 | 0 | 0 | 3 | 319 | 0 |
| LB_pharmacophore22 | 0 | 4 | 1 | 2 | 0 | 0 | 0 | 0 | 0 | 0 | 1 | 320 | 0 |

Table S11. FXR_alpha agonist dataset. Pharmacophore features composition of the SBLB agonist selective pharmacophores (AR: aromatic ring, H:hydrophobic, HBA: Hydrogen Bond Acceptor, HBD: Hydrogen Bond Donor, PI: Positive ionizable area, NI: negative ionisable area, opt.: feature set as optional) and number of agonist ligands found with each pharmacophore (“Nbr of agonist ligands found”), number of unique agonist ligands found by combining the pharmacophores (“Accumulated nbr of unique agonist ligands found”) and number of decoys found (“Nbr of antagonist ligands found”)

|  | Pharmacophore features | | | | | | | | | | Number of positive and negative hits | | |
| --- | --- | --- | --- | --- | --- | --- | --- | --- | --- | --- | --- | --- | --- |
|  | AR | H | HBA | HBD | PI | NI | AR opt. | H opt. | HBA opt. | HBD opt. | Nbr of antagonist ligands found | Accumulated nbr of unique antagonist ligands found | Nbr of agonist ligands found |
| LB_pharmacophore1 | 0 | 5 | 2 | 0 | 0 | 0 | 0 | 0 | 0 | 0 | 5 | 5 | 0 |
| LB_pharmacophore2 | 4 | 6 | 4 | 1 | 0 | 1 | 0 | 0 | 0 | 0 | 5 | 10 | 0 |
| LB_pharmacophore3 | 0 | 3 | 1 | 1 | 0 | 0 | 0 | 0 | 0 | 0 | 5 | 15 | 0 |
| LB_pharmacophore4 | 0 | 6 | 2 | 1 | 0 | 0 | 0 | 0 | 0 | 0 | 4 | 18 | 0 |
| LB_pharmacophore5 | 1 | 5 | 2 | 1 | 0 | 0 | 0 | 0 | 0 | 0 | 4 | 21 | 0 |
| LB_pharmacophore6 | 0 | 5 | 0 | 0 | 0 | 0 | 0 | 0 | 0 | 0 | 3 | 24 | 0 |
| LB_pharmacophore7 | 0 | 3 | 2 | 0 | 0 | 0 | 0 | 0 | 0 | 0 | 2 | 26 | 0 |
| LB_pharmacophore8 | 0 | 2 | 2 | 2 | 0 | 0 | 0 | 0 | 0 | 0 | 1 | 27 | 0 |
| LB_pharmacophore9 | 0 | 3 | 2 | 0 | 0 | 0 | 0 | 0 | 0 | 0 | 1 | 28 | 0 |

Table S12. FXR_alpha antagonist dataset. Pharmacophore features composition of the SBLB agonist selective pharmacophores (AR: aromatic ring, H:hydrophobic, HBA: Hydrogen Bond Acceptor, HBD: Hydrogen Bond Donor, PI: Positive ionizable area, NI: negative ionisable area, opt.: feature set as optional) and number of antagonist ligands found with each pharmacophore (“Nbr of antagonist ligands found”), number of unique antagonist ligands found by combining the pharmacophores (“Accumulated nbr of unique antagonist ligands found”) and number of decoys found (“Nbr of agonist ligands found”)

|  | Pharmacophore features | | | | | | | | | | Number of positive and negative hits | | |
| --- | --- | --- | --- | --- | --- | --- | --- | --- | --- | --- | --- | --- | --- |
|  | AR | H | HBA | HBD | PI | NI | AR opt. | H opt. | HBA opt. | HBD opt. | Nbr of agonist ligands found | Accumulated nbr of unique agonist ligands found | Nbr of antagonist ligands found |
| LB_pharmacophore1 | 1 | 1 | 1 | 1 | 0 | 0 | 0 | 0 | 0 | 0 | 85 | 85 | 0 |
| LB_pharmacophore2 | 0 | 1 | 2 | 1 | 0 | 0 | 0 | 0 | 0 | 0 | 73 | 140 | 0 |
| LB_pharmacophore3 | 1 | 3 | 2 | 0 | 0 | 0 | 0 | 0 | 1 | 0 | 63 | 158 | 0 |
| LB_pharmacophore4 | 0 | 1 | 2 | 1 | 0 | 0 | 0 | 0 | 0 | 0 | 61 | 170 | 0 |
| LB_pharmacophore5 | 0 | 0 | 3 | 1 | 0 | 0 | 0 | 0 | 0 | 0 | 56 | 177 | 0 |
| LB_pharmacophore6 | 2 | 2 | 0 | 2 | 0 | 0 | 0 | 0 | 0 | 0 | 53 | 200 | 0 |
| LB_pharmacophore7 | 0 | 2 | 3 | 0 | 0 | 0 | 0 | 0 | 0 | 0 | 48 | 208 | 0 |
| LB_pharmacophore8 | 0 | 3 | 1 | 1 | 0 | 0 | 0 | 0 | 0 | 0 | 38 | 211 | 0 |
| LB_pharmacophore9 | 0 | 3 | 1 | 1 | 0 | 0 | 0 | 0 | 0 | 0 | 26 | 219 | 0 |
| LB_pharmacophore10 | 0 | 1 | 1 | 2 | 0 | 0 | 0 | 0 | 0 | 0 | 24 | 227 | 0 |
| LB_pharmacophore11 | 1 | 1 | 1 | 1 | 0 | 0 | 0 | 0 | 0 | 0 | 24 | 238 | 0 |
| LB_pharmacophore12 | 1 | 1 | 1 | 1 | 0 | 0 | 0 | 0 | 0 | 0 | 19 | 246 | 0 |
| LB_pharmacophore13 | 1 | 3 | 2 | 1 | 0 | 0 | 0 | 0 | 0 | 0 | 18 | 257 | 0 |
| LB_pharmacophore14 | 1 | 6 | 0 | 1 | 0 | 0 | 0 | 0 | 0 | 0 | 16 | 262 | 0 |
| LB_pharmacophore15 | 2 | 1 | 1 | 0 | 0 | 0 | 0 | 0 | 0 | 0 | 16 | 270 | 0 |
| LB_pharmacophore16 | 2 | 4 | 0 | 1 | 0 | 0 | 0 | 0 | 0 | 0 | 14 | 272 | 0 |
| SB_pharmacophore17 | 0 | 2 | 1 | 1 | 0 | 0 | 0 | 0 | 0 | 0 | 12 | 277 | 0 |
| LB_pharmacophore18 | 0 | 0 | 4 | 1 | 0 | 0 | 0 | 0 | 0 | 0 | 12 | 281 | 0 |
| LB_pharmacophore19 | 1 | 1 | 1 | 1 | 0 | 0 | 0 | 0 | 0 | 0 | 12 | 283 | 0 |
| LB_pharmacophore20 | 0 | 2 | 2 | 0 | 0 | 0 | 0 | 0 | 1 | 0 | 12 | 286 | 0 |
| LB_pharmacophore21 | 0 | 4 | 1 | 1 | 0 | 0 | 0 | 0 | 0 | 0 | 6 | 287 | 0 |
| LB_pharmacophore22 | 1 | 4 | 6 | 0 | 0 | 0 | 0 | 0 | 0 | 0 | 3 | 290 | 0 |

Table S13. (continued next page)

|  | Pharmacophore features | | | | | | | | | | Number of positive and negative hits | | |
| --- | --- | --- | --- | --- | --- | --- | --- | --- | --- | --- | --- | --- | --- |
|  | AR | H | HBA | HBD | PI | NI | AR opt. | H opt. | HBA opt. | HBD opt. | Nbr of agonist ligands found | Accumulated nbr of unique agonist ligands found | Nbr of antagonist ligands found |
| LB_pharmacophore23 | 2 | 8 | 2 | 2 | 0 | 0 | 0 | 0 | 0 | 0 | 3 | 292 | 0 |
| LB_pharmacophore24 | 3 | 6 | 0 | 1 | 0 | 0 | 0 | 0 | 0 | 0 | 1 | 293 | 0 |

Table S13. GR agonist dataset. Pharmacophore features composition of the SBLB agonist selective pharmacophores (AR: aromatic ring, H:hydrophobic, HBA: Hydrogen Bond Acceptor, HBD: Hydrogen Bond Donor, PI: Positive ionizable area, NI: negative ionisable area, opt.: feature set as optional) and number of agonist ligands found with each pharmacophore (“Nbr of agonist ligands found”), number of unique agonist ligands found by combining the pharmacophores (“Accumulated nbr of unique agonist ligands found”) and number of decoys found (“Nbr of antagonist ligands found”)

|  | Pharmacophore features | | | | | | | | | | Number of positive and negative hits | | |
| --- | --- | --- | --- | --- | --- | --- | --- | --- | --- | --- | --- | --- | --- |
|  | AR | H | HBA | HBD | PI | NI | AR opt. | H opt. | HBA opt. | HBD opt. | Nbr of antagonist ligands found | Accumulated nbr of unique antagonist ligands found | Nbr of agonist ligands found |
| LB_pharmacophore1 | 0 | 2 | 2 | 0 | 0 | 0 | 0 | 0 | 0 | 0 | 68 | 68 | 0 |
| LB_pharmacophore2 | 1 | 1 | 2 | 0 | 0 | 0 | 0 | 0 | 0 | 0 | 65 | 103 | 0 |
| LB_pharmacophore3 | 0 | 2 | 3 | 0 | 0 | 0 | 0 | 0 | 0 | 0 | 63 | 156 | 0 |
| LB_pharmacophore4 | 0 | 1 | 2 | 0 | 0 | 1 | 0 | 0 | 0 | 0 | 61 | 197 | 0 |
| LB_pharmacophore5 | 1 | 2 | 1 | 0 | 0 | 0 | 0 | 0 | 0 | 0 | 59 | 215 | 0 |
| LB_pharmacophore6 | 0 | 3 | 1 | 0 | 0 | 0 | 0 | 0 | 0 | 0 | 57 | 225 | 0 |
| LB_pharmacophore7 | 1 | 2 | 0 | 0 | 0 | 0 | 0 | 0 | 0 | 0 | 53 | 254 | 0 |
| LB_pharmacophore8 | 0 | 2 | 2 | 0 | 0 | 0 | 0 | 0 | 0 | 0 | 52 | 256 | 0 |
| LB_pharmacophore9 | 0 | 1 | 2 | 1 | 0 | 0 | 0 | 0 | 0 | 0 | 49 | 279 | 0 |
| LB_pharmacophore10 | 1 | 2 | 2 | 0 | 0 | 0 | 0 | 0 | 0 | 0 | 45 | 280 | 0 |
| LB_pharmacophore11 | 2 | 2 | 0 | 0 | 0 | 0 | 0 | 0 | 0 | 0 | 39 | 283 | 0 |
| LB_pharmacophore12 | 1 | 2 | 1 | 0 | 0 | 0 | 0 | 0 | 0 | 0 | 39 | 285 | 0 |
| LB_pharmacophore13 | 0 | 4 | 0 | 1 | 0 | 0 | 1 | 0 | 1 | 0 | 37 | 302 | 0 |
| LB_pharmacophore14 | 0 | 2 | 2 | 0 | 0 | 0 | 0 | 0 | 0 | 0 | 30 | 310 | 0 |
| LB_pharmacophore15 | 1 | 1 | 2 | 0 | 0 | 0 | 0 | 0 | 0 | 0 | 28 | 331 | 0 |
| LB_pharmacophore16 | 0 | 3 | 2 | 0 | 0 | 0 | 0 | 0 | 0 | 0 | 27 | 332 | 0 |
| LB_pharmacophore17 | 1 | 2 | 2 | 2 | 0 | 0 | 0 | 0 | 0 | 0 | 22 | 333 | 0 |
| LB_pharmacophore18 | 0 | 1 | 2 | 1 | 0 | 0 | 0 | 0 | 0 | 0 | 20 | 337 | 0 |
| LB_pharmacophore19 | 1 | 2 | 0 | 1 | 0 | 0 | 0 | 0 | 0 | 0 | 18 | 341 | 0 |
| LB_pharmacophore20 | 0 | 3 | 3 | 0 | 0 | 0 | 0 | 0 | 0 | 0 | 17 | 344 | 0 |
| LB_pharmacophore21 | 1 | 4 | 1 | 0 | 0 | 0 | 0 | 0 | 1 | 0 | 16 | 346 | 0 |

Table S14. (continued next page)

|  | Pharmacophore features | | | | | | | | | | Number of positive and negative hits | | |
| --- | --- | --- | --- | --- | --- | --- | --- | --- | --- | --- | --- | --- | --- |
|  | AR | H | HBA | HBD | PI | NI | AR opt. | H opt. | HBA opt. | HBD opt. | Nbr of antagonist ligands found | Accumulated nbr of unique antagonist ligands found | Nbr of agonist ligands found |
| LB_pharmacophore22 | 1 | 3 | 2 | 0 | 0 | 0 | 0 | 0 | 0 | 0 | 14 | 350 | 0 |
| LB_pharmacophore23 | 1 | 2 | 0 | 1 | 0 | 0 | 0 | 0 | 0 | 0 | 13 | 351 | 0 |
| LB_pharmacophore24 | 0 | 1 | 2 | 1 | 0 | 0 | 0 | 0 | 0 | 0 | 12 | 352 | 0 |
| LB_pharmacophore25 | 0 | 2 | 2 | 0 | 0 | 0 | 0 | 0 | 0 | 0 | 8 | 354 | 0 |
| LB_pharmacophore26 | 2 | 3 | 3 | 2 | 0 | 0 | 0 | 0 | 0 | 0 | 7 | 356 | 0 |
| LB_pharmacophore27 | 1 | 3 | 1 | 1 | 0 | 0 | 1 | 1 | 0 | 0 | 6 | 359 | 0 |
| LB_pharmacophore28 | 0 | 1 | 2 | 1 | 0 | 0 | 0 | 0 | 0 | 0 | 6 | 361 | 0 |
| LB_pharmacophore29 | 0 | 2 | 3 | 0 | 0 | 0 | 0 | 0 | 0 | 0 | 1 | 362 | 0 |
| LB_pharmacophore30 | 2 | 4 | 5 | 1 | 0 | 0 | 0 | 0 | 0 | 0 | 1 | 363 | 0 |
| LB_pharmacophore31 | 3 | 8 | 0 | 2 | 0 | 0 | 0 | 1 | 0 | 0 | 1 | 364 | 0 |
| LB_pharmacophore32 | 3 | 7 | 0 | 1 | 0 | 0 | 0 | 0 | 0 | 0 | 1 | 365 | 0 |

Table S14. GR antagonist dataset. Pharmacophore features composition of the SBLB agonist selective pharmacophores (AR: aromatic ring, H:hydrophobic, HBA: Hydrogen Bond Acceptor, HBD: Hydrogen Bond Donor, PI: Positive ionizable area, NI: negative ionisable area, opt.: feature set as optional) and number of antagonist ligands found with each pharmacophore (“Nbr of antagonist ligands found”), number of unique antagonist ligands found by combining the pharmacophores (“Accumulated nbr of unique antagonist ligands found”) and number of decoys found (“Nbr of agonist ligands found”)

|  | Pharmacophore features | | | | | | | | | | Number of positive and negative hits | | |
| --- | --- | --- | --- | --- | --- | --- | --- | --- | --- | --- | --- | --- | --- |
|  | AR | H | HBA | HBD | PI | NI | AR opt. | H opt. | HBA opt. | HBD opt. | Nbr of agonist ligands found | Accumulated nbr of unique agonist ligands found | Nbr of antagonist ligands found |
| LB_pharmacophore1 | 1 | 1 | 2 | 0 | 0 | 0 | 0 | 0 | 0 | 0 | 143 | 143 | 0 |
| LB_pharmacophore2 | 0 | 1 | 4 | 0 | 0 | 0 | 0 | 0 | 0 | 0 | 143 | 169 | 0 |
| LB_pharmacophore3 | 0 | 2 | 1 | 1 | 0 | 0 | 0 | 0 | 0 | 1 | 80 | 179 | 0 |
| LB_pharmacophore4 | 0 | 2 | 1 | 1 | 0 | 0 | 0 | 1 | 1 | 1 | 72 | 181 | 0 |
| LB_pharmacophore5 | 1 | 2 | 2 | 0 | 0 | 0 | 0 | 0 | 0 | 0 | 57 | 195 | 0 |
| LB_pharmacophore6 | 2 | 3 | 1 | 0 | 0 | 0 | 0 | 0 | 0 | 0 | 52 | 206 | 0 |
| LB_pharmacophore7 | 0 | 3 | 2 | 0 | 0 | 0 | 0 | 0 | 0 | 0 | 45 | 213 | 0 |
| LB_pharmacophore8 | 2 | 1 | 1 | 0 | 0 | 0 | 0 | 0 | 0 | 0 | 45 | 220 | 0 |
| LB_pharmacophore9 | 1 | 1 | 0 | 1 | 0 | 0 | 0 | 0 | 0 | 0 | 44 | 229 | 0 |
| LB_pharmacophore10 | 0 | 2 | 3 | 0 | 0 | 0 | 0 | 0 | 0 | 0 | 43 | 230 | 0 |
| LB_pharmacophore11 | 0 | 3 | 2 | 0 | 0 | 0 | 0 | 0 | 0 | 0 | 41 | 235 | 0 |
| LB_pharmacophore12 | 1 | 2 | 3 | 0 | 0 | 0 | 0 | 0 | 0 | 0 | 28 | 242 | 0 |
| LB_pharmacophore13 | 0 | 1 | 1 | 1 | 0 | 0 | 0 | 0 | 0 | 0 | 24 | 249 | 0 |
| LB_pharmacophore14 | 0 | 4 | 1 | 0 | 0 | 0 | 0 | 0 | 0 | 0 | 19 | 256 | 0 |
| LB_pharmacophore15 | 1 | 1 | 2 | 1 | 0 | 0 | 0 | 0 | 0 | 0 | 18 | 257 | 0 |
| LB_pharmacophore16 | 0 | 2 | 2 | 0 | 0 | 0 | 0 | 0 | 0 | 0 | 15 | 258 | 0 |

Table S15. LXR_alpha agonist dataset. Pharmacophore features composition of the SBLB agonist selective pharmacophores (AR: aromatic ring, H:hydrophobic, HBA: Hydrogen Bond Acceptor, HBD: Hydrogen Bond Donor, PI: Positive ionizable area, NI: negative ionisable area, opt.: feature set as optional) and number of agonist ligands found with each pharmacophore (“Nbr of agonist ligands found”), number of unique agonist ligands found by combining the pharmacophores (“Accumulated nbr of unique agonist ligands found”) and number of decoys found (“Nbr of antagonist ligands found”)

|  | Pharmacophore features | | | | | | | | | | Number of positive and negative hits | | |
| --- | --- | --- | --- | --- | --- | --- | --- | --- | --- | --- | --- | --- | --- |
|  | AR | H | HBA | HBD | PI | NI | AR opt. | H opt. | HBA opt. | HBD opt. | Nbr of antagonist ligands found | Accumulated nbr of unique antagonist ligands found | Nbr of agonist ligands found |
| LB_pharmacophore1 | 0 | 4 | 2 | 0 | 0 | 0 | 0 | 0 | 0 | 0 | 19 | 19 | 0 |
| LB_pharmacophore2 | 1 | 2 | 2 | 0 | 0 | 0 | 0 | 0 | 0 | 0 | 15 | 26 | 0 |
| LB_pharmacophore3 | 1 | 2 | 2 | 0 | 0 | 0 | 0 | 0 | 0 | 0 | 14 | 28 | 0 |
| LB_pharmacophore4 | 4 | 4 | 0 | 0 | 0 | 0 | 0 | 0 | 0 | 0 | 11 | 39 | 0 |
| LB_pharmacophore5 | 2 | 1 | 1 | 0 | 0 | 0 | 0 | 0 | 0 | 0 | 9 | 45 | 0 |
| LB_pharmacophore6 | 2 | 4 | 1 | 0 | 0 | 0 | 0 | 0 | 0 | 0 | 5 | 47 | 0 |
| LB_pharmacophore7 | 0 | 3 | 3 | 0 | 0 | 0 | 0 | 0 | 0 | 0 | 2 | 49 | 0 |

Table S16. LXR_alpha antagonist dataset. Pharmacophore features composition of the SBLB agonist selective pharmacophores (AR: aromatic ring, H:hydrophobic, HBA: Hydrogen Bond Acceptor, HBD: Hydrogen Bond Donor, PI: Positive ionizable area, NI: negative ionisable area, opt.: feature set as optional) and number of antagonist ligands found with each pharmacophore (“Nbr of antagonist ligands found”), number of unique antagonist ligands found by combining the pharmacophores (“Accumulated nbr of unique antagonist ligands found”) and number of decoys found (“Nbr of agonist ligands found”)

|  | Pharmacophore features | | | | | | | | | | Number of positive and negative hits | | |
| --- | --- | --- | --- | --- | --- | --- | --- | --- | --- | --- | --- | --- | --- |
|  | AR | H | HBA | HBD | PI | NI | AR opt. | H opt. | HBA opt. | HBD opt. | Nbr of agonist ligands found | Accumulated nbr of unique agonist ligands found | Nbr of antagonist ligands found |
| LB_pharmacophore1 | 2 | 2 | 0 | 0 | 0 | 0 | 0 | 0 | 0 | 0 | 220 | 220 | 0 |
| LB_pharmacophore2 | 0 | 2 | 3 | 0 | 0 | 0 | 0 | 0 | 0 | 0 | 215 | 311 | 0 |
| LB_pharmacophore3 | 0 | 1 | 3 | 0 | 0 | 0 | 0 | 0 | 0 | 0 | 198 | 312 | 0 |
| LB_pharmacophore4 | 0 | 1 | 3 | 0 | 0 | 0 | 0 | 0 | 0 | 0 | 159 | 319 | 0 |
| LB_pharmacophore5 | 1 | 2 | 1 | 0 | 0 | 0 | 0 | 0 | 0 | 0 | 120 | 328 | 0 |
| SB_pharmacophore6 | 0 | 3 | 1 | 0 | 0 | 0 | 0 | 0 | 0 | 0 | 91 | 330 | 0 |
| LB_pharmacophore7 | 0 | 1 | 3 | 0 | 0 | 0 | 0 | 0 | 0 | 0 | 70 | 349 | 0 |
| LB_pharmacophore8 | 0 | 2 | 2 | 0 | 0 | 0 | 0 | 0 | 0 | 0 | 64 | 357 | 0 |
| LB_pharmacophore9 | 1 | 2 | 1 | 0 | 0 | 0 | 0 | 0 | 0 | 0 | 41 | 366 | 0 |
| LB_pharmacophore10 | 1 | 2 | 2 | 0 | 0 | 0 | 0 | 0 | 0 | 0 | 37 | 367 | 0 |
| LB_pharmacophore11 | 1 | 1 | 0 | 0 | 1 | 0 | 0 | 0 | 0 | 0 | 19 | 368 | 0 |
| LB_pharmacophore12 | 1 | 2 | 1 | 0 | 0 | 0 | 1 | 1 | 2 | 1 | 15 | 373 | 0 |

Table S17. LXR_beta agonist dataset. Pharmacophore features composition of the SBLB agonist selective pharmacophores (AR: aromatic ring, H:hydrophobic, HBA: Hydrogen Bond Acceptor, HBD: Hydrogen Bond Donor, PI: Positive ionizable area, NI: negative ionisable area, opt.: feature set as optional) and number of agonist ligands found with each pharmacophore (“Nbr of agonist ligands found”), number of unique agonist ligands found by combining the pharmacophores (“Accumulated nbr of unique agonist ligands found”) and number of decoys found (“Nbr of antagonist ligands found”)

|  | Pharmacophore features | | | | | | | | | | Number of positive and negative hits | | |
| --- | --- | --- | --- | --- | --- | --- | --- | --- | --- | --- | --- | --- | --- |
|  | AR | H | HBA | HBD | PI | NI | AR opt. | H opt. | HBA opt. | HBD opt. | Nbr of antagonist ligands found | Accumulated nbr of unique antagonist ligands found | Nbr of agonist ligands found |
| LB_pharmacophore1 | 4 | 4 | 0 | 0 | 0 | 0 | 0 | 0 | 0 | 0 | 12 | 12 | 0 |
| LB_pharmacophore2 | 0 | 5 | 6 | 0 | 0 | 0 | 0 | 0 | 0 | 0 | 10 | 22 | 0 |
| LB_pharmacophore3 | 1 | 5 | 2 | 0 | 0 | 0 | 0 | 0 | 0 | 0 | 8 | 30 | 0 |
| LB_pharmacophore4 | 1 | 1 | 2 | 0 | 0 | 0 | 0 | 0 | 0 | 0 | 7 | 33 | 0 |
| LB_pharmacophore5 | 1 | 1 | 2 | 0 | 0 | 0 | 0 | 0 | 0 | 0 | 6 | 35 | 0 |
| LB_pharmacophore6 | 2 | 1 | 2 | 0 | 0 | 0 | 0 | 0 | 0 | 0 | 4 | 37 | 0 |
| LB_pharmacophore7 | 2 | 1 | 2 | 1 | 0 | 0 | 0 | 0 | 0 | 0 | 2 | 38 | 0 |

Table S18. LXR_beta antagonist dataset. Pharmacophore features composition of the SBLB agonist selective pharmacophores (AR: aromatic ring, H:hydrophobic, HBA: Hydrogen Bond Acceptor, HBD: Hydrogen Bond Donor, PI: Positive ionizable area, NI: negative ionisable area, opt.: feature set as optional) and number of antagonist ligands found with each pharmacophore (“Nbr of antagonist ligands found”), number of unique antagonist ligands found by combining the pharmacophores (“Accumulated nbr of unique antagonist ligands found”) and number of decoys found (“Nbr of agonist ligands found”)

|  | Pharmacophore features | | | | | | | | | | Number of positive and negative hits | | |
| --- | --- | --- | --- | --- | --- | --- | --- | --- | --- | --- | --- | --- | --- |
|  | AR | H | HBA | HBD | PI | NI | AR opt. | H opt. | HBA opt. | HBD opt. | Nbr of agonist ligands found | Accumulated nbr of unique agonist ligands found | Nbr of antagonist ligands found |
| LB_pharmacophore1 | 0 | 1 | 3 | 0 | 0 | 0 | 0 | 0 | 0 | 0 | 6 | 6 | 0 |
| SB_pharmacophore2 | 0 | 2 | 2 | 0 | 0 | 0 | 0 | 0 | 0 | 0 | 5 | 7 | 0 |
| LB_pharmacophore3 | 0 | 2 | 2 | 1 | 0 | 0 | 0 | 0 | 0 | 0 | 2 | 9 | 0 |

Table S19. MR agonist dataset. Pharmacophore features composition of the SBLB agonist selective pharmacophores (AR: aromatic ring, H:hydrophobic, HBA: Hydrogen Bond Acceptor, HBD: Hydrogen Bond Donor, PI: Positive ionizable area, NI: negative ionisable area, opt.: feature set as optional) and number of agonist ligands found with each pharmacophore (“Nbr of agonist ligands found”), number of unique agonist ligands found by combining the pharmacophores (“Accumulated nbr of unique agonist ligands found”) and number of decoys found (“Nbr of antagonist ligands found”)

|  | Pharmacophore features | | | | | | | | | | Number of positive and negative hits | | |
| --- | --- | --- | --- | --- | --- | --- | --- | --- | --- | --- | --- | --- | --- |
|  | AR | H | HBA | HBD | PI | NI | AR opt. | H opt. | HBA opt. | HBD opt. | Nbr of antagonist ligands found | Accumulated nbr of unique antagonist ligands found | Nbr of agonist ligands found |
| LB_pharmacophore1 | 0 | 4 | 0 | 0 | 0 | 0 | 0 | 0 | 0 | 0 | 81 | 81 | 0 |
| LB_pharmacophore2 | 0 | 3 | 1 | 0 | 0 | 0 | 0 | 0 | 0 | 0 | 45 | 92 | 0 |
| LB_pharmacophore3 | 0 | 2 | 3 | 0 | 0 | 0 | 0 | 0 | 0 | 0 | 43 | 113 | 0 |
| LB_pharmacophore4 | 0 | 1 | 2 | 0 | 0 | 0 | 0 | 1 | 0 | 0 | 39 | 125 | 0 |
| LB_pharmacophore5 | 0 | 2 | 1 | 1 | 0 | 0 | 0 | 0 | 0 | 0 | 38 | 135 | 0 |
| LB_pharmacophore6 | 0 | 1 | 2 | 1 | 0 | 0 | 0 | 0 | 0 | 0 | 36 | 139 | 0 |
| LB_pharmacophore7 | 1 | 3 | 0 | 0 | 0 | 0 | 0 | 0 | 0 | 0 | 22 | 140 | 0 |
| LB_pharmacophore8 | 0 | 1 | 3 | 0 | 0 | 0 | 0 | 0 | 0 | 0 | 18 | 143 | 0 |
| LB_pharmacophore9 | 1 | 2 | 1 | 0 | 0 | 0 | 0 | 0 | 0 | 0 | 17 | 145 | 0 |

Table S20. MR antagonist dataset. Pharmacophore features composition of the SBLB agonist selective pharmacophores (AR: aromatic ring, H:hydrophobic, HBA: Hydrogen Bond Acceptor, HBD: Hydrogen Bond Donor, PI: Positive ionizable area, NI: negative ionisable area, opt.: feature set as optional) and number of antagonist ligands found with each pharmacophore (“Nbr of antagonist ligands found”), number of unique antagonist ligands found by combining the pharmacophores (“Accumulated nbr of unique antagonist ligands found”) and number of decoys found (“Nbr of agonist ligands found”)

|  | Pharmacophore features | | | | | | | | | | Number of positive and negative hits | | |
| --- | --- | --- | --- | --- | --- | --- | --- | --- | --- | --- | --- | --- | --- |
|  | AR | H | HBA | HBD | PI | NI | AR opt. | H opt. | HBA opt. | HBD opt. | Nbr of agonist ligands found | Accumulated nbr of unique agonist ligands found | Nbr of antagonist ligands found |
| LB_pharmacophore1 | 0 | 1 | 2 | 0 | 0 | 1 | 0 | 0 | 0 | 0 | 1158 | 1158 | 0 |
| LB_pharmacophore2 | 0 | 1 | 2 | 0 | 0 | 1 | 0 | 0 | 0 | 0 | 1079 | 1255 | 0 |
| LB_pharmacophore3 | 0 | 1 | 2 | 0 | 0 | 1 | 0 | 0 | 0 | 0 | 876 | 1296 | 0 |
| SB_pharmacophore4 | 0 | 1 | 3 | 0 | 0 | 0 | 0 | 0 | 0 | 0 | 727 | 1323 | 0 |
| LB_pharmacophore5 | 1 | 2 | 1 | 0 | 0 | 0 | 0 | 0 | 0 | 0 | 521 | 1341 | 0 |
| SB_pharmacophore6 | 0 | 2 | 2 | 0 | 0 | 0 | 0 | 0 | 0 | 0 | 399 | 1346 | 0 |
| LB_pharmacophore7 | 2 | 2 | 0 | 0 | 0 | 0 | 0 | 0 | 0 | 0 | 242 | 1349 | 0 |
| LB_pharmacophore8 | 1 | 1 | 2 | 0 | 0 | 0 | 0 | 0 | 0 | 0 | 202 | 1359 | 0 |
| LB_pharmacophore9 | 1 | 2 | 2 | 0 | 0 | 0 | 0 | 0 | 0 | 0 | 188 | 1362 | 0 |
| LB_pharmacophore10 | 2 | 2 | 0 | 0 | 0 | 0 | 0 | 0 | 0 | 0 | 169 | 1369 | 0 |
| LB_pharmacophore11 | 1 | 1 | 2 | 0 | 0 | 0 | 0 | 0 | 0 | 0 | 163 | 1373 | 0 |
| LB_pharmacophore12 | 2 | 2 | 0 | 0 | 0 | 0 | 0 | 0 | 0 | 0 | 123 | 1381 | 0 |
| SB_pharmacophore13 | 0 | 2 | 2 | 0 | 0 | 0 | 0 | 0 | 0 | 0 | 91 | 1385 | 0 |
| LB_pharmacophore14 | 2 | 2 | 0 | 0 | 0 | 0 | 0 | 0 | 0 | 0 | 80 | 1386 | 0 |
| LB_pharmacophore15 | 0 | 3 | 1 | 0 | 0 | 0 | 0 | 0 | 0 | 0 | 31 | 1387 | 0 |
| LB_pharmacophore16 | 1 | 3 | 1 | 0 | 0 | 0 | 0 | 0 | 0 | 0 | 29 | 1388 | 0 |
| LB_pharmacophore17 | 2 | 2 | 1 | 1 | 1 | 0 | 0 | 0 | 0 | 0 | 5 | 1391 | 0 |
| LB_pharmacophore18 | 2 | 2 | 4 | 1 | 0 | 0 | 0 | 0 | 0 | 0 | 2 | 1392 | 0 |
| LB_pharmacophore19 | 0 | 1 | 2 | 2 | 0 | 0 | 0 | 0 | 0 | 0 | 1 | 1393 | 0 |

Table S21. PPAR_alpha agonist dataset. Pharmacophore features composition of the SBLB agonist selective pharmacophores (AR: aromatic ring, H:hydrophobic, HBA: Hydrogen Bond Acceptor, HBD: Hydrogen Bond Donor, PI: Positive ionizable area, NI: negative ionisable area, opt.: feature set as optional) and number of agonist ligands found with each pharmacophore (“Nbr of agonist ligands found”), number of unique agonist ligands found by combining the pharmacophores (“Accumulated nbr of unique agonist ligands found”) and number of decoys found (“Nbr of antagonist ligands found”)

|  | Pharmacophore features | | | | | | | | | | Number of positive and negative hits | | |
| --- | --- | --- | --- | --- | --- | --- | --- | --- | --- | --- | --- | --- | --- |
|  | AR | H | HBA | HBD | PI | NI | AR opt. | H opt. | HBA opt. | HBD opt. | Nbr of antagonist ligands found | Accumulated nbr of unique antagonist ligands found | Nbr of agonist ligands found |
| LB_pharmacophore1 | 0 | 1 | 3 | 1 | 0 | 0 | 0 | 0 | 0 | 0 | 5 | 5 | 0 |
| LB_pharmacophore2 | 1 | 5 | 1 | 0 | 0 | 0 | 0 | 0 | 0 | 0 | 1 | 6 | 0 |
| LB_pharmacophore3 | 0 | 3 | 2 | 1 | 0 | 0 | 0 | 0 | 0 | 0 | 1 | 7 | 0 |

Table S22. PPAR_alpha antagonist dataset. Pharmacophore features composition of the SBLB agonist selective pharmacophores (AR: aromatic ring, H:hydrophobic, HBA: Hydrogen Bond Acceptor, HBD: Hydrogen Bond Donor, PI: Positive ionizable area, NI: negative ionisable area, opt.: feature set as optional) and number of antagonist ligands found with each pharmacophore (“Nbr of antagonist ligands found”), number of unique antagonist ligands found by combining the pharmacophores (“Accumulated nbr of unique antagonist ligands found”) and number of decoys found (“Nbr of agonist ligands found”)

|  | Pharmacophore features | | | | | | | | | | Number of positive and negative hits | | |
| --- | --- | --- | --- | --- | --- | --- | --- | --- | --- | --- | --- | --- | --- |
|  | AR | H | HBA | HBD | PI | NI | AR opt. | H opt. | HBA opt. | HBD opt. | Nbr of agonist ligands found | Accumulated nbr of unique agonist ligands found | Nbr of antagonist ligands found |
| LB_pharmacophore1 | 0 | 1 | 2 | 0 | 0 | 1 | 0 | 0 | 0 | 0 | 843 | 843 | 0 |
| LB_pharmacophore2 | 0 | 1 | 2 | 0 | 0 | 1 | 0 | 0 | 0 | 0 | 496 | 860 | 0 |
| LB_pharmacophore3 | 0 | 1 | 2 | 0 | 0 | 1 | 0 | 0 | 0 | 0 | 321 | 863 | 0 |
| LB_pharmacophore4 | 0 | 1 | 3 | 0 | 0 | 0 | 0 | 0 | 0 | 0 | 95 | 867 | 0 |
| LB_pharmacophore5 | 1 | 2 | 2 | 0 | 0 | 0 | 0 | 0 | 0 | 0 | 93 | 868 | 0 |
| LB_pharmacophore6 | 1 | 2 | 1 | 0 | 0 | 0 | 0 | 0 | 0 | 0 | 76 | 873 | 0 |
| LB_pharmacophore7 | 1 | 2 | 2 | 0 | 0 | 0 | 0 | 0 | 0 | 0 | 69 | 878 | 0 |
| LB_pharmacophore8 | 1 | 2 | 2 | 0 | 0 | 0 | 0 | 0 | 0 | 0 | 41 | 884 | 0 |
| LB_pharmacophore9 | 1 | 3 | 1 | 0 | 0 | 0 | 0 | 0 | 0 | 0 | 41 | 887 | 0 |
| LB_pharmacophore10 | 0 | 2 | 5 | 0 | 0 | 1 | 0 | 0 | 0 | 0 | 27 | 888 | 0 |
| LB_pharmacophore11 | 0 | 3 | 1 | 1 | 0 | 0 | 0 | 1 | 0 | 0 | 20 | 890 | 0 |
| LB_pharmacophore12 | 1 | 1 | 3 | 0 | 0 | 0 | 0 | 0 | 0 | 0 | 17 | 891 | 0 |
| LB_pharmacophore13 | 0 | 1 | 1 | 1 | 1 | 0 | 0 | 0 | 0 | 0 | 6 | 894 | 0 |
| LB_pharmacophore14 | 0 | 1 | 2 | 1 | 0 | 0 | 0 | 0 | 0 | 0 | 4 | 896 | 0 |
| LB_pharmacophore15 | 2 | 2 | 3 | 1 | 0 | 0 | 0 | 0 | 0 | 0 | 2 | 897 | 0 |

Table S23. PPAR_beta agonist dataset. Pharmacophore features composition of the SBLB agonist selective pharmacophores (AR: aromatic ring, H:hydrophobic, HBA: Hydrogen Bond Acceptor, HBD: Hydrogen Bond Donor, PI: Positive ionizable area, NI: negative ionisable area, opt.: feature set as optional) and number of agonist ligands found with each pharmacophore (“Nbr of agonist ligands found”), number of unique agonist ligands found by combining the pharmacophores (“Accumulated nbr of unique agonist ligands found”) and number of decoys found (“Nbr of antagonist ligands found”)

|  | Pharmacophore features | | | | | | | | | | Number of positive and negative hits | | |
| --- | --- | --- | --- | --- | --- | --- | --- | --- | --- | --- | --- | --- | --- |
|  | AR | H | HBA | HBD | PI | NI | AR opt. | H opt. | HBA opt. | HBD opt. | Nbr of antagonist ligands found | Accumulated nbr of unique antagonist ligands found | Nbr of agonist ligands found |
| LB_pharmacophore1 | 1 | 4 | 6 | 1 | 0 | 0 | 0 | 0 | 0 | 0 | 7 | 7 | 0 |
| LB_pharmacophore2 | 2 | 5 | 8 | 1 | 0 | 0 | 0 | 0 | 0 | 0 | 1 | 8 | 0 |
| LB_pharmacophore3 | 2 | 2 | 2 | 1 | 0 | 0 | 0 | 0 | 0 | 0 | 1 | 9 | 0 |
| LB_pharmacophore4 | 0 | 3 | 4 | 0 | 0 | 0 | 0 | 0 | 0 | 0 | 1 | 10 | 0 |

Table S24. PPAR_beta antagonist dataset. Pharmacophore features composition of the SBLB agonist selective pharmacophores (AR: aromatic ring, H:hydrophobic, HBA: Hydrogen Bond Acceptor, HBD: Hydrogen Bond Donor, PI: Positive ionizable area, NI: negative ionisable area, opt.: feature set as optional) and number of antagonist ligands found with each pharmacophore (“Nbr of antagonist ligands found”), number of unique antagonist ligands found by combining the pharmacophores (“Accumulated nbr of unique antagonist ligands found”) and number of decoys found (“Nbr of agonist ligands found”)

|  | Pharmacophore features | | | | | | | | | | Number of positive and negative hits | | |
| --- | --- | --- | --- | --- | --- | --- | --- | --- | --- | --- | --- | --- | --- |
|  | AR | H | HBA | HBD | PI | NI | AR opt. | H opt. | HBA opt. | HBD opt. | Nbr of agonist ligands found | Accumulated nbr of unique agonist ligands found | Nbr of antagonist ligands found |
| LB_pharmacophore1 | 0 | 1 | 3 | 0 | 0 | 0 | 0 | 0 | 0 | 0 | 1299 | 1299 | 0 |
| SB_pharmacophore2 | 0 | 2 | 2 | 0 | 0 | 0 | 0 | 0 | 0 | 0 | 885 | 1445 | 0 |
| SB_pharmacophore3 | 0 | 2 | 2 | 0 | 0 | 0 | 0 | 0 | 0 | 0 | 526 | 1480 | 0 |
| LB_pharmacophore4 | 0 | 1 | 2 | 0 | 0 | 1 | 0 | 0 | 0 | 0 | 462 | 1496 | 0 |
| SB_pharmacophore5 | 0 | 0 | 3 | 0 | 0 | 1 | 0 | 0 | 0 | 0 | 458 | 1522 | 0 |
| SB_pharmacophore6 | 1 | 3 | 0 | 0 | 0 | 0 | 0 | 0 | 0 | 0 | 426 | 1536 | 0 |
| SB_pharmacophore7 | 0 | 1 | 3 | 0 | 0 | 0 | 0 | 0 | 0 | 0 | 415 | 1548 | 0 |
| SB_pharmacophore8 | 0 | 2 | 2 | 0 | 0 | 0 | 0 | 0 | 0 | 0 | 388 | 1568 | 0 |
| SB_pharmacophore9 | 0 | 3 | 1 | 0 | 0 | 0 | 0 | 0 | 0 | 0 | 386 | 1601 | 0 |
| SB_pharmacophore10 | 0 | 4 | 0 | 0 | 0 | 0 | 0 | 0 | 0 | 0 | 370 | 1614 | 0 |
| SB_pharmacophore11 | 0 | 2 | 2 | 0 | 0 | 0 | 0 | 0 | 0 | 0 | 347 | 1668 | 0 |
| SB_pharmacophore12 | 0 | 3 | 1 | 0 | 0 | 0 | 0 | 0 | 0 | 0 | 312 | 1686 | 0 |
| SB_pharmacophore13 | 0 | 0 | 3 | 0 | 0 | 1 | 0 | 0 | 0 | 0 | 290 | 1687 | 0 |
| LB_pharmacophore14 | 0 | 1 | 3 | 0 | 0 | 0 | 0 | 0 | 0 | 0 | 253 | 1693 | 0 |
| SB_pharmacophore15 | 0 | 1 | 3 | 0 | 0 | 0 | 0 | 0 | 0 | 0 | 248 | 1707 | 0 |
| SB_pharmacophore16 | 0 | 4 | 0 | 0 | 0 | 0 | 0 | 0 | 0 | 0 | 244 | 1717 | 0 |
| SB_pharmacophore17 | 0 | 3 | 1 | 0 | 0 | 0 | 0 | 0 | 0 | 0 | 221 | 1723 | 0 |
| SB_pharmacophore18 | 0 | 3 | 1 | 0 | 0 | 0 | 0 | 0 | 0 | 0 | 201 | 1727 | 0 |
| SB_pharmacophore19 | 0 | 4 | 0 | 0 | 0 | 0 | 0 | 0 | 0 | 0 | 185 | 1730 | 0 |
| LB_pharmacophore20 | 1 | 2 | 1 | 0 | 0 | 0 | 0 | 0 | 0 | 0 | 158 | 1742 | 0 |
| LB_pharmacophore21 | 0 | 4 | 1 | 0 | 0 | 0 | 0 | 0 | 0 | 0 | 147 | 1745 | 0 |
| SB_pharmacophore22 | 0 | 1 | 3 | 0 | 0 | 0 | 0 | 0 | 0 | 0 | 147 | 1748 | 0 |

Table S25. (continued next page)

|  | Pharmacophore features | | | | | | | | | | Number of positive and negative hits | | |
| --- | --- | --- | --- | --- | --- | --- | --- | --- | --- | --- | --- | --- | --- |
|  | AR | H | HBA | HBD | PI | NI | AR opt. | H opt. | HBA opt. | HBD opt. | Nbr of agonist ligands found | Accumulated nbr of unique agonist ligands found | Nbr of antagonist ligands found |
| SB_pharmacophore23 | 0 | 2 | 2 | 0 | 0 | 0 | 0 | 0 | 0 | 0 | 145 | 1756 | 0 |
| SB_pharmacophore24 | 0 | 2 | 2 | 0 | 0 | 0 | 0 | 0 | 0 | 0 | 139 | 1764 | 0 |
| SB_pharmacophore25 | 0 | 1 | 2 | 0 | 0 | 1 | 0 | 0 | 0 | 0 | 138 | 1770 | 0 |
| SB_pharmacophore26 | 0 | 2 | 2 | 0 | 0 | 0 | 0 | 0 | 0 | 0 | 133 | 1782 | 0 |
| SB_pharmacophore27 | 0 | 3 | 1 | 0 | 0 | 0 | 0 | 0 | 0 | 0 | 107 | 1784 | 0 |
| SB_pharmacophore28 | 0 | 3 | 0 | 1 | 0 | 0 | 0 | 0 | 0 | 0 | 95 | 1787 | 0 |
| LB_pharmacophore29 | 0 | 2 | 3 | 0 | 0 | 1 | 0 | 0 | 0 | 0 | 87 | 1790 | 0 |
| SB_pharmacophore30 | 0 | 4 | 0 | 0 | 0 | 0 | 0 | 0 | 0 | 0 | 82 | 1791 | 0 |
| LB_pharmacophore31 | 1 | 2 | 2 | 0 | 0 | 0 | 0 | 0 | 0 | 0 | 80 | 1793 | 0 |
| LB_pharmacophore32 | 1 | 2 | 2 | 0 | 0 | 0 | 0 | 0 | 0 | 0 | 48 | 1797 | 0 |
| LB_pharmacophore33 | 0 | 1 | 3 | 0 | 0 | 1 | 0 | 0 | 0 | 0 | 48 | 1801 | 0 |
| LB_pharmacophore34 | 0 | 1 | 2 | 1 | 0 | 0 | 0 | 0 | 0 | 0 | 43 | 1805 | 0 |
| LB_pharmacophore35 | 2 | 0 | 2 | 0 | 0 | 0 | 0 | 0 | 0 | 0 | 29 | 1806 | 0 |
| LB_pharmacophore36 | 0 | 1 | 2 | 1 | 0 | 0 | 0 | 0 | 0 | 0 | 23 | 1809 | 0 |
| LB_pharmacophore37 | 0 | 1 | 2 | 1 | 0 | 0 | 0 | 0 | 0 | 0 | 13 | 1813 | 0 |
| LB_pharmacophore38 | 1 | 2 | 1 | 1 | 0 | 0 | 0 | 0 | 0 | 0 | 6 | 1815 | 0 |
| LB_pharmacophore39 | 0 | 1 | 4 | 1 | 0 | 1 | 0 | 0 | 0 | 0 | 1 | 1816 | 0 |

Table S25. PPAR_gamma agonist dataset. Pharmacophore features composition of the SBLB agonist selective pharmacophores (AR: aromatic ring, H:hydrophobic, HBA: Hydrogen Bond Acceptor, HBD: Hydrogen Bond Donor, PI: Positive ionizable area, NI: negative ionisable area, opt.: feature set as optional) and number of agonist ligands found with each pharmacophore (“Nbr of agonist ligands found”), number of unique agonist ligands found by combining the pharmacophores (“Accumulated nbr of unique agonist ligands found”) and number of decoys found (“Nbr of antagonist ligands found”)

|  | Pharmacophore features | | | | | | | | | | Number of positive and negative hits | | |
| --- | --- | --- | --- | --- | --- | --- | --- | --- | --- | --- | --- | --- | --- |
|  | AR | H | HBA | HBD | PI | NI | AR opt. | H opt. | HBA opt. | HBD opt. | Nbr of antagonist ligands found | Accumulated nbr of unique antagonist ligands found | Nbr of agonist ligands found |
| LB_pharmacophore1 | 2 | 2 | 3 | 0 | 0 | 0 | 0 | 0 | 0 | 0 | 3 | 3 | 0 |
| LB_pharmacophore2 | 1 | 1 | 1 | 0 | 0 | 0 | 0 | 0 | 0 | 0 | 3 | 6 | 0 |
| LB_pharmacophore3 | 2 | 4 | 5 | 1 | 0 | 0 | 0 | 0 | 0 | 0 | 2 | 7 | 0 |
| LB_pharmacophore4 | 0 | 0 | 5 | 0 | 0 | 1 | 0 | 0 | 0 | 0 | 2 | 8 | 0 |
| LB_pharmacophore5 | 1 | 0 | 3 | 1 | 0 | 0 | 0 | 0 | 0 | 0 | 1 | 9 | 0 |

Table S26. PPAR_gamma antagonist dataset. Pharmacophore features composition of the SBLB agonist selective pharmacophores (AR: aromatic ring, H:hydrophobic, HBA: Hydrogen Bond Acceptor, HBD: Hydrogen Bond Donor, PI: Positive ionizable area, NI: negative ionisable area, opt.: feature set as optional) and number of antagonist ligands found with each pharmacophore (“Nbr of antagonist ligands found”), number of unique antagonist ligands found by combining the pharmacophores (“Accumulated nbr of unique antagonist ligands found”) and number of decoys found (“Nbr of agonist ligands found”)

|  | Pharmacophore features | | | | | | | | | | Number of positive and negative hits | | |
| --- | --- | --- | --- | --- | --- | --- | --- | --- | --- | --- | --- | --- | --- |
|  | AR | H | HBA | HBD | PI | NI | AR opt. | H opt. | HBA opt. | HBD opt. | Nbr of agonist ligands found | Accumulated nbr of unique agonist ligands found | Nbr of antagonist ligands found |
| LB_pharmacophore1 | 1 | 4 | 0 | 1 | 0 | 0 | 0 | 0 | 0 | 0 | 55 | 55 | 0 |
| LB_pharmacophore2 | 1 | 2 | 3 | 1 | 0 | 0 | 0 | 0 | 0 | 0 | 36 | 91 | 0 |
| LB_pharmacophore3 | 2 | 2 | 1 | 1 | 0 | 0 | 0 | 0 | 0 | 0 | 35 | 100 | 0 |
| LB_pharmacophore4 | 1 | 3 | 1 | 0 | 0 | 0 | 0 | 0 | 0 | 0 | 35 | 134 | 0 |
| LB_pharmacophore5 | 2 | 6 | 0 | 1 | 0 | 0 | 0 | 0 | 0 | 0 | 33 | 140 | 0 |
| LB_pharmacophore6 | 2 | 0 | 3 | 1 | 0 | 0 | 0 | 0 | 0 | 0 | 32 | 154 | 0 |
| LB_pharmacophore7 | 2 | 2 | 1 | 0 | 0 | 0 | 0 | 0 | 0 | 0 | 30 | 156 | 0 |
| LB_pharmacophore8 | 2 | 7 | 0 | 1 | 0 | 0 | 0 | 0 | 0 | 0 | 24 | 158 | 0 |
| LB_pharmacophore9 | 1 | 7 | 0 | 1 | 0 | 0 | 0 | 0 | 0 | 0 | 23 | 159 | 0 |
| LB_pharmacophore10 | 3 | 4 | 0 | 1 | 0 | 0 | 0 | 0 | 0 | 0 | 22 | 161 | 0 |
| LB_pharmacophore11 | 2 | 1 | 2 | 1 | 0 | 0 | 0 | 0 | 0 | 0 | 20 | 165 | 0 |
| LB_pharmacophore12 | 2 | 3 | 3 | 0 | 0 | 0 | 0 | 0 | 0 | 0 | 15 | 175 | 0 |
| LB_pharmacophore13 | 1 | 4 | 1 | 1 | 0 | 0 | 0 | 0 | 0 | 0 | 15 | 190 | 0 |
| LB_pharmacophore14 | 0 | 3 | 2 | 0 | 0 | 0 | 0 | 0 | 0 | 0 | 11 | 195 | 0 |
| LB_pharmacophore15 | 0 | 1 | 3 | 1 | 0 | 0 | 0 | 0 | 0 | 0 | 11 | 197 | 0 |
| LB_pharmacophore16 | 0 | 2 | 2 | 0 | 0 | 0 | 0 | 0 | 0 | 0 | 10 | 198 | 0 |
| LB_pharmacophore17 | 0 | 2 | 3 | 1 | 0 | 0 | 0 | 0 | 0 | 0 | 9 | 205 | 0 |
| LB_pharmacophore18 | 1 | 2 | 2 | 2 | 0 | 0 | 0 | 0 | 0 | 0 | 9 | 206 | 0 |
| LB_pharmacophore19 | 2 | 4 | 2 | 0 | 0 | 0 | 0 | 0 | 0 | 0 | 8 | 208 | 0 |
| LB_pharmacophore20 | 2 | 3 | 2 | 1 | 0 | 0 | 0 | 2 | 0 | 0 | 8 | 212 | 0 |
| LB_pharmacophore21 | 1 | 4 | 3 | 0 | 0 | 0 | 0 | 0 | 0 | 0 | 8 | 213 | 0 |
| LB_pharmacophore22 | 2 | 5 | 2 | 1 | 0 | 0 | 0 | 0 | 0 | 0 | 8 | 214 | 0 |

Table S27. (continued next page)

|  | Pharmacophore features | | | | | | | | | | Number of positive and negative hits | | |
| --- | --- | --- | --- | --- | --- | --- | --- | --- | --- | --- | --- | --- | --- |
|  | AR | H | HBA | HBD | PI | NI | AR opt. | H opt. | HBA opt. | HBD opt. | Nbr of agonist ligands found | Accumulated nbr of unique agonist ligands found | Nbr of antagonist ligands found |
| LB_pharmacophore23 | 2 | 7 | 1 | 1 | 0 | 0 | 0 | 0 | 0 | 0 | 8 | 215 | 0 |
| LB_pharmacophore24 | 1 | 2 | 1 | 1 | 0 | 0 | 0 | 0 | 0 | 0 | 8 | 218 | 0 |
| LB_pharmacophore25 | 1 | 2 | 2 | 1 | 0 | 0 | 0 | 0 | 0 | 0 | 7 | 220 | 0 |
| LB_pharmacophore26 | 0 | 2 | 2 | 1 | 0 | 0 | 0 | 0 | 0 | 0 | 7 | 223 | 0 |
| LB_pharmacophore27 | 0 | 4 | 2 | 1 | 0 | 0 | 0 | 1 | 0 | 0 | 6 | 224 | 0 |
| LB_pharmacophore28 | 1 | 2 | 2 | 1 | 0 | 0 | 0 | 0 | 0 | 0 | 5 | 226 | 0 |
| LB_pharmacophore29 | 1 | 3 | 0 | 0 | 1 | 0 | 0 | 0 | 0 | 0 | 5 | 228 | 0 |
| LB_pharmacophore30 | 2 | 4 | 2 | 1 | 0 | 0 | 0 | 0 | 0 | 0 | 5 | 229 | 0 |
| LB_pharmacophore31 | 1 | 4 | 1 | 1 | 0 | 0 | 0 | 0 | 0 | 0 | 5 | 231 | 0 |
| SB_pharmacophore32 | 0 | 2 | 4 | 1 | 0 | 0 | 0 | 0 | 0 | 0 | 4 | 235 | 0 |
| LB_pharmacophore33 | 3 | 4 | 3 | 0 | 0 | 0 | 0 | 0 | 0 | 0 | 4 | 236 | 0 |
| LB_pharmacophore34 | 1 | 3 | 2 | 1 | 0 | 0 | 0 | 0 | 0 | 0 | 3 | 237 | 0 |
| LB_pharmacophore35 | 1 | 3 | 2 | 1 | 0 | 0 | 0 | 0 | 0 | 0 | 3 | 238 | 0 |
| LB_pharmacophore36 | 0 | 6 | 2 | 1 | 0 | 0 | 0 | 0 | 0 | 0 | 3 | 239 | 0 |
| LB_pharmacophore37 | 0 | 3 | 3 | 0 | 0 | 0 | 0 | 0 | 0 | 0 | 2 | 241 | 0 |
| LB_pharmacophore38 | 0 | 2 | 2 | 1 | 0 | 0 | 0 | 0 | 0 | 0 | 2 | 243 | 0 |
| LB_pharmacophore39 | 0 | 1 | 3 | 1 | 0 | 0 | 0 | 0 | 0 | 0 | 2 | 244 | 0 |
| LB_pharmacophore40 | 0 | 3 | 3 | 0 | 0 | 0 | 0 | 0 | 0 | 0 | 2 | 246 | 0 |
| LB_pharmacophore41 | 0 | 2 | 5 | 0 | 0 | 0 | 0 | 0 | 0 | 0 | 2 | 247 | 0 |
| LB_pharmacophore42 | 0 | 3 | 2 | 0 | 0 | 0 | 0 | 0 | 0 | 0 | 2 | 248 | 0 |
| LB_pharmacophore43 | 0 | 2 | 2 | 0 | 0 | 0 | 0 | 0 | 0 | 0 | 2 | 250 | 0 |
| LB_pharmacophore44 | 0 | 1 | 2 | 0 | 0 | 0 | 0 | 0 | 0 | 0 | 2 | 251 | 0 |

Table S27. (continued next page)

|  | Pharmacophore features | | | | | | | | | | Number of positive and negative hits | | |
| --- | --- | --- | --- | --- | --- | --- | --- | --- | --- | --- | --- | --- | --- |
|  | AR | H | HBA | HBD | PI | NI | AR opt. | H opt. | HBA opt. | HBD opt. | Nbr of agonist ligands found | Accumulated nbr of unique agonist ligands found | Nbr of antagonist ligands found |
| LB_pharmacophore45 | 3 | 7 | 1 | 1 | 0 | 0 | 0 | 1 | 0 | 0 | 1 | 252 | 0 |
| LB_pharmacophore46 | 2 | 6 | 0 | 1 | 0 | 0 | 0 | 0 | 0 | 0 | 1 | 253 | 0 |
| LB_pharmacophore47 | 2 | 4 | 2 | 1 | 0 | 0 | 0 | 2 | 1 | 0 | 1 | 254 | 0 |
| LB_pharmacophore48 | 2 | 3 | 3 | 1 | 0 | 0 | 0 | 0 | 0 | 0 | 1 | 255 | 0 |
| LB_pharmacophore49 | 0 | 2 | 2 | 2 | 0 | 0 | 0 | 0 | 0 | 0 | 1 | 256 | 0 |
| LB_pharmacophore50 | 1 | 1 | 2 | 2 | 0 | 0 | 0 | 0 | 0 | 0 | 1 | 257 | 0 |
| LB_pharmacophore51 | 1 | 4 | 2 | 1 | 0 | 0 | 0 | 0 | 0 | 0 | 1 | 258 | 0 |
| LB_pharmacophore52 | 2 | 3 | 3 | 1 | 0 | 0 | 0 | 0 | 0 | 0 | 1 | 259 | 0 |

Table S27. PR agonist dataset. Pharmacophore features composition of the SBLB agonist selective pharmacophores (AR: aromatic ring, H:hydrophobic, HBA: Hydrogen Bond Acceptor, HBD: Hydrogen Bond Donor, PI: Positive ionizable area, NI: negative ionisable area, opt.: feature set as optional) and number of agonist ligands found with each pharmacophore (“Nbr of agonist ligands found”), number of unique agonist ligands found by combining the pharmacophores (“Accumulated nbr of unique agonist ligands found”) and number of decoys found (“Nbr of antagonist ligands found”)

|  | Pharmacophore features | | | | | | | | | | Number of positive and negative hits | | |
| --- | --- | --- | --- | --- | --- | --- | --- | --- | --- | --- | --- | --- | --- |
|  | AR | H | HBA | HBD | PI | NI | AR opt. | H opt. | HBA opt. | HBD opt. | Nbr of antagonist ligands found | Accumulated nbr of unique antagonist ligands found | Nbr of agonist ligands found |
| LB_pharmacophore1 | 2 | 1 | 1 | 0 | 0 | 0 | 0 | 0 | 0 | 0 | 74 | 74 | 0 |
| LB_pharmacophore2 | 1 | 1 | 3 | 0 | 0 | 0 | 0 | 0 | 0 | 0 | 57 | 114 | 0 |
| LB_pharmacophore3 | 1 | 3 | 2 | 0 | 0 | 0 | 0 | 0 | 0 | 0 | 56 | 170 | 0 |
| LB_pharmacophore4 | 1 | 2 | 2 | 1 | 0 | 0 | 0 | 0 | 0 | 0 | 50 | 184 | 0 |
| LB_pharmacophore5 | 0 | 3 | 1 | 0 | 0 | 0 | 0 | 0 | 0 | 0 | 49 | 225 | 0 |
| LB_pharmacophore6 | 2 | 1 | 1 | 0 | 0 | 0 | 0 | 0 | 0 | 0 | 47 | 228 | 0 |
| SB_pharmacophore7 | 0 | 1 | 3 | 0 | 0 | 0 | 0 | 0 | 0 | 0 | 45 | 266 | 0 |
| LB_pharmacophore8 | 1 | 2 | 1 | 0 | 0 | 0 | 0 | 0 | 0 | 0 | 40 | 299 | 0 |
| LB_pharmacophore9 | 2 | 0 | 1 | 1 | 0 | 0 | 0 | 0 | 0 | 0 | 32 | 307 | 0 |
| LB_pharmacophore10 | 1 | 2 | 1 | 1 | 0 | 0 | 0 | 0 | 0 | 0 | 32 | 321 | 0 |
| LB_pharmacophore11 | 1 | 2 | 2 | 1 | 0 | 0 | 0 | 0 | 0 | 0 | 30 | 323 | 0 |
| LB_pharmacophore12 | 2 | 3 | 3 | 1 | 0 | 0 | 0 | 0 | 0 | 0 | 30 | 344 | 0 |
| LB_pharmacophore13 | 2 | 3 | 1 | 1 | 0 | 0 | 0 | 0 | 0 | 0 | 29 | 348 | 0 |
| LB_pharmacophore14 | 1 | 2 | 2 | 0 | 0 | 0 | 0 | 0 | 0 | 0 | 27 | 351 | 0 |
| LB_pharmacophore15 | 2 | 1 | 1 | 0 | 0 | 0 | 0 | 0 | 0 | 0 | 26 | 354 | 0 |
| LB_pharmacophore16 | 1 | 3 | 1 | 1 | 0 | 0 | 0 | 0 | 0 | 0 | 25 | 360 | 0 |
| LB_pharmacophore17 | 2 | 2 | 1 | 1 | 0 | 0 | 0 | 0 | 0 | 0 | 25 | 372 | 0 |
| LB_pharmacophore18 | 1 | 2 | 2 | 0 | 0 | 0 | 0 | 0 | 0 | 0 | 24 | 373 | 0 |
| LB_pharmacophore19 | 3 | 1 | 0 | 0 | 0 | 0 | 0 | 0 | 0 | 0 | 23 | 374 | 0 |
| LB_pharmacophore20 | 2 | 3 | 1 | 0 | 0 | 0 | 0 | 0 | 0 | 0 | 22 | 384 | 0 |
| LB_pharmacophore21 | 0 | 3 | 2 | 1 | 0 | 0 | 0 | 0 | 0 | 0 | 22 | 385 | 0 |

Table S28. (continued next page)

|  | Pharmacophore features | | | | | | | | | | Number of positive and negative hits | | |
| --- | --- | --- | --- | --- | --- | --- | --- | --- | --- | --- | --- | --- | --- |
|  | AR | H | HBA | HBD | PI | NI | AR opt. | H opt. | HBA opt. | HBD opt. | Nbr of antagonist ligands found | Accumulated nbr of unique antagonist ligands found | Nbr of agonist ligands found |
| LB_pharmacophore22 | 1 | 1 | 2 | 0 | 0 | 0 | 0 | 0 | 0 | 0 | 21 | 399 | 0 |
| LB_pharmacophore23 | 2 | 1 | 1 | 0 | 0 | 0 | 0 | 0 | 0 | 0 | 21 | 409 | 0 |
| LB_pharmacophore24 | 2 | 4 | 0 | 1 | 0 | 0 | 1 | 1 | 0 | 0 | 20 | 429 | 0 |
| LB_pharmacophore25 | 1 | 3 | 2 | 0 | 0 | 0 | 0 | 0 | 0 | 0 | 18 | 430 | 0 |
| LB_pharmacophore26 | 2 | 1 | 0 | 1 | 0 | 0 | 0 | 0 | 0 | 0 | 18 | 432 | 0 |
| LB_pharmacophore27 | 1 | 2 | 1 | 0 | 0 | 0 | 0 | 0 | 0 | 0 | 17 | 433 | 0 |
| LB_pharmacophore28 | 0 | 0 | 3 | 1 | 0 | 0 | 0 | 0 | 0 | 0 | 15 | 438 | 0 |
| LB_pharmacophore29 | 0 | 1 | 3 | 0 | 0 | 0 | 0 | 0 | 0 | 0 | 15 | 439 | 0 |
| LB_pharmacophore30 | 0 | 2 | 2 | 0 | 0 | 0 | 0 | 0 | 0 | 0 | 15 | 440 | 0 |
| LB_pharmacophore31 | 1 | 2 | 3 | 1 | 0 | 0 | 0 | 0 | 0 | 0 | 15 | 443 | 0 |
| LB_pharmacophore32 | 1 | 1 | 2 | 0 | 0 | 0 | 0 | 0 | 0 | 0 | 14 | 453 | 0 |
| LB_pharmacophore33 | 2 | 3 | 2 | 1 | 0 | 0 | 0 | 0 | 0 | 0 | 14 | 457 | 0 |
| LB_pharmacophore34 | 2 | 2 | 2 | 1 | 0 | 0 | 0 | 0 | 0 | 0 | 13 | 460 | 0 |
| LB_pharmacophore35 | 1 | 0 | 2 | 1 | 0 | 0 | 0 | 0 | 0 | 0 | 13 | 461 | 0 |
| LB_pharmacophore36 | 1 | 1 | 3 | 0 | 0 | 0 | 0 | 1 | 0 | 1 | 12 | 468 | 0 |
| LB_pharmacophore37 | 2 | 3 | 1 | 0 | 0 | 0 | 0 | 0 | 0 | 0 | 12 | 472 | 0 |
| LB_pharmacophore38 | 1 | 3 | 1 | 0 | 0 | 0 | 0 | 0 | 0 | 0 | 11 | 473 | 0 |
| LB_pharmacophore39 | 2 | 1 | 2 | 1 | 0 | 0 | 0 | 0 | 0 | 0 | 11 | 475 | 0 |
| LB_pharmacophore40 | 1 | 4 | 1 | 0 | 0 | 0 | 0 | 0 | 0 | 0 | 9 | 481 | 0 |
| LB_pharmacophore41 | 2 | 4 | 1 | 1 | 0 | 0 | 0 | 0 | 0 | 0 | 9 | 482 | 0 |
| LB_pharmacophore42 | 0 | 3 | 2 | 0 | 0 | 0 | 0 | 0 | 0 | 0 | 9 | 483 | 0 |

Table S28. (continued next page)

|  | Pharmacophore features | | | | | | | | | | Number of positive and negative hits | | |
| --- | --- | --- | --- | --- | --- | --- | --- | --- | --- | --- | --- | --- | --- |
|  | AR | H | HBA | HBD | PI | NI | AR opt. | H opt. | HBA opt. | HBD opt. | Nbr of antagonist ligands found | Accumulated nbr of unique antagonist ligands found | Nbr of agonist ligands found |
| LB_pharmacophore43 | 1 | 2 | 1 | 1 | 0 | 0 | 0 | 0 | 0 | 0 | 9 | 490 | 0 |
| LB_pharmacophore44 | 1 | 3 | 0 | 1 | 0 | 0 | 0 | 0 | 0 | 0 | 8 | 492 | 0 |
| LB_pharmacophore45 | 1 | 2 | 3 | 0 | 0 | 0 | 0 | 0 | 0 | 0 | 7 | 494 | 0 |
| LB_pharmacophore46 | 2 | 4 | 1 | 1 | 0 | 0 | 0 | 0 | 0 | 0 | 7 | 495 | 0 |
| LB_pharmacophore47 | 2 | 2 | 1 | 1 | 0 | 0 | 0 | 0 | 0 | 0 | 7 | 500 | 0 |
| LB_pharmacophore48 | 0 | 3 | 1 | 1 | 0 | 0 | 0 | 0 | 0 | 0 | 7 | 501 | 0 |
| LB_pharmacophore49 | 1 | 3 | 2 | 1 | 0 | 0 | 0 | 0 | 0 | 0 | 7 | 503 | 0 |
| LB_pharmacophore50 | 2 | 2 | 1 | 1 | 0 | 0 | 0 | 0 | 0 | 0 | 7 | 505 | 0 |
| LB_pharmacophore51 | 1 | 4 | 1 | 0 | 0 | 0 | 0 | 0 | 0 | 0 | 7 | 507 | 0 |
| LB_pharmacophore52 | 2 | 3 | 2 | 1 | 0 | 0 | 0 | 0 | 0 | 0 | 6 | 508 | 0 |
| LB_pharmacophore53 | 2 | 6 | 0 | 0 | 0 | 0 | 0 | 0 | 1 | 0 | 6 | 512 | 0 |
| LB_pharmacophore54 | 2 | 3 | 0 | 1 | 0 | 0 | 0 | 0 | 0 | 0 | 6 | 515 | 0 |
| LB_pharmacophore55 | 2 | 3 | 2 | 1 | 0 | 0 | 0 | 0 | 0 | 0 | 6 | 516 | 0 |
| LB_pharmacophore56 | 1 | 3 | 2 | 0 | 0 | 0 | 0 | 0 | 0 | 0 | 6 | 517 | 0 |
| LB_pharmacophore57 | 0 | 5 | 1 | 0 | 0 | 0 | 0 | 0 | 0 | 0 | 5 | 519 | 0 |
| LB_pharmacophore58 | 1 | 2 | 2 | 1 | 0 | 0 | 0 | 0 | 0 | 0 | 4 | 521 | 0 |
| LB_pharmacophore59 | 2 | 5 | 1 | 1 | 0 | 0 | 0 | 0 | 0 | 0 | 4 | 523 | 0 |
| LB_pharmacophore60 | 1 | 3 | 2 | 1 | 0 | 0 | 0 | 0 | 0 | 0 | 3 | 524 | 0 |
| LB_pharmacophore61 | 2 | 5 | 3 | 1 | 0 | 0 | 0 | 0 | 0 | 0 | 3 | 526 | 0 |
| LB_pharmacophore62 | 3 | 5 | 3 | 1 | 0 | 0 | 0 | 0 | 0 | 0 | 2 | 527 | 0 |

Table S28. (continued next page)

|  | Pharmacophore features | | | | | | | | | | Number of positive and negative hits | | |
| --- | --- | --- | --- | --- | --- | --- | --- | --- | --- | --- | --- | --- | --- |
|  | AR | H | HBA | HBD | PI | NI | AR opt. | H opt. | HBA opt. | HBD opt. | Nbr of antagonist ligands found | Accumulated nbr of unique antagonist ligands found | Nbr of agonist ligands found |
| LB_pharmacophore63 | 2 | 4 | 4 | 1 | 0 | 0 | 0 | 0 | 0 | 0 | 1 | 528 | 0 |
| LB_pharmacophore64 | 1 | 1 | 1 | 2 | 0 | 0 | 0 | 0 | 0 | 0 | 1 | 529 | 0 |

Table S28. PR antagonist dataset. Pharmacophore features composition of the SBLB agonist selective pharmacophores (AR: aromatic ring, H:hydrophobic, HBA: Hydrogen Bond Acceptor, HBD: Hydrogen Bond Donor, PI: Positive ionizable area, NI: negative ionisable area, opt.: feature set as optional) and number of antagonist ligands found with each pharmacophore (“Nbr of antagonist ligands found”), number of unique antagonist ligands found by combining the pharmacophores (“Accumulated nbr of unique antagonist ligands found”) and number of decoys found (“Nbr of agonist ligands found”)

|  | Pharmacophore features | | | | | | | | | | Number of positive and negative hits | | |
| --- | --- | --- | --- | --- | --- | --- | --- | --- | --- | --- | --- | --- | --- |
|  | AR | H | HBA | HBD | PI | NI | AR opt. | H opt. | HBA opt. | HBD opt. | Nbr of agonist ligands found | Accumulated nbr of unique agonist ligands found | Nbr of antagonist ligands found |
| LB_pharmacophore1 | 0 | 1 | 2 | 0 | 0 | 0 | 0 | 0 | 0 | 0 | 54 | 54 | 0 |
| SB_pharmacophore2 | 0 | 2 | 2 | 0 | 0 | 0 | 0 | 0 | 0 | 0 | 47 | 67 | 0 |
| SB_pharmacophore3 | 0 | 3 | 1 | 0 | 0 | 0 | 0 | 0 | 0 | 0 | 45 | 72 | 0 |
| LB_pharmacophore4 | 0 | 0 | 3 | 0 | 0 | 0 | 0 | 0 | 0 | 0 | 40 | 77 | 0 |
| SB_pharmacophore5 | 0 | 2 | 2 | 0 | 0 | 0 | 0 | 0 | 0 | 0 | 26 | 79 | 0 |
| LB_pharmacophore6 | 0 | 1 | 3 | 0 | 0 | 0 | 0 | 0 | 0 | 0 | 21 | 80 | 0 |
| LB_pharmacophore7 | 0 | 2 | 1 | 1 | 0 | 0 | 0 | 0 | 0 | 0 | 18 | 89 | 0 |
| LB_pharmacophore8 | 1 | 2 | 1 | 0 | 0 | 0 | 0 | 0 | 0 | 0 | 8 | 90 | 0 |
| LB_pharmacophore9 | 0 | 0 | 4 | 1 | 0 | 0 | 0 | 0 | 0 | 0 | 8 | 91 | 0 |
| LB_pharmacophore10 | 0 | 3 | 2 | 0 | 0 | 0 | 0 | 0 | 0 | 0 | 7 | 95 | 0 |
| LB_pharmacophore11 | 1 | 2 | 1 | 0 | 0 | 0 | 0 | 0 | 0 | 0 | 6 | 96 | 0 |
| LB_pharmacophore12 | 0 | 2 | 2 | 0 | 0 | 0 | 0 | 0 | 0 | 0 | 6 | 98 | 0 |
| LB_pharmacophore13 | 2 | 4 | 2 | 1 | 0 | 0 | 0 | 0 | 0 | 0 | 3 | 99 | 0 |
| LB_pharmacophore14 | 0 | 4 | 6 | 3 | 0 | 0 | 0 | 0 | 0 | 0 | 2 | 100 | 0 |

Table S29. PXR agonist dataset. Pharmacophore features composition of the SBLB agonist selective pharmacophores (AR: aromatic ring, H:hydrophobic, HBA: Hydrogen Bond Acceptor, HBD: Hydrogen Bond Donor, PI: Positive ionizable area, NI: negative ionisable area, opt.: feature set as optional) and number of agonist ligands found with each pharmacophore (“Nbr of agonist ligands found”), number of unique agonist ligands found by combining the pharmacophores (“Accumulated nbr of unique agonist ligands found”) and number of decoys found (“Nbr of antagonist ligands found”)

|  | Pharmacophore features | | | | | | | | | | Number of positive and negative hits | | |
| --- | --- | --- | --- | --- | --- | --- | --- | --- | --- | --- | --- | --- | --- |
|  | AR | H | HBA | HBD | PI | NI | AR opt. | H opt. | HBA opt. | HBD opt. | Nbr of antagonist ligands found | Accumulated nbr of unique antagonist ligands found | Nbr of agonist ligands found |
| LB_pharmacophore1 | 0 | 0 | 3 | 2 | 0 | 0 | 1 | 0 | 0 | 0 | 4 | 4 | 0 |
| LB_pharmacophore2 | 1 | 4 | 4 | 0 | 1 | 0 | 0 | 0 | 0 | 0 | 2 | 6 | 0 |
| LB_pharmacophore3 | 1 | 1 | 2 | 2 | 0 | 0 | 0 | 0 | 0 | 0 | 2 | 7 | 0 |

Table S30. PXR antagonist dataset. Pharmacophore features composition of the SBLB agonist selective pharmacophores (AR: aromatic ring, H:hydrophobic, HBA: Hydrogen Bond Acceptor, HBD: Hydrogen Bond Donor, PI: Positive ionizable area, NI: negative ionisable area, opt.: feature set as optional) and number of antagonist ligands found with each pharmacophore (“Nbr of antagonist ligands found”), number of unique antagonist ligands found by combining the pharmacophores (“Accumulated nbr of unique antagonist ligands found”) and number of decoys found (“Nbr of agonist ligands found”)

|  | Pharmacophore features | | | | | | | | | | Number of positive and negative hits | | |
| --- | --- | --- | --- | --- | --- | --- | --- | --- | --- | --- | --- | --- | --- |
|  | AR | H | HBA | HBD | PI | NI | AR opt. | H opt. | HBA opt. | HBD opt. | Nbr of agonist ligands found | Accumulated nbr of unique agonist ligands found | Nbr of antagonist ligands found |
| LB_pharmacophore1 | 0 | 2 | 2 | 0 | 0 | 1 | 0 | 0 | 0 | 0 | 57 | 57 | 0 |
| LB_pharmacophore2 | 1 | 2 | 2 | 0 | 0 | 0 | 0 | 0 | 0 | 0 | 50 | 79 | 0 |
| SB_pharmacophore3 | 0 | 3 | 2 | 0 | 0 | 1 | 0 | 0 | 0 | 0 | 43 | 94 | 0 |
| LB_pharmacophore4 | 1 | 4 | 1 | 0 | 0 | 1 | 0 | 0 | 0 | 0 | 37 | 95 | 0 |
| LB_pharmacophore5 | 2 | 2 | 0 | 0 | 0 | 0 | 0 | 0 | 0 | 0 | 33 | 107 | 0 |
| SB_pharmacophore6 | 0 | 4 | 1 | 0 | 0 | 1 | 0 | 0 | 0 | 0 | 30 | 111 | 0 |
| LB_pharmacophore7 | 0 | 4 | 1 | 0 | 0 | 1 | 0 | 0 | 0 | 0 | 21 | 114 | 0 |
| LB_pharmacophore8 | 2 | 0 | 1 | 0 | 0 | 1 | 0 | 0 | 0 | 0 | 18 | 116 | 0 |
| LB_pharmacophore9 | 1 | 6 | 2 | 0 | 0 | 1 | 0 | 0 | 0 | 0 | 14 | 117 | 0 |
| LB_pharmacophore10 | 1 | 1 | 2 | 1 | 0 | 1 | 0 | 0 | 0 | 0 | 12 | 118 | 0 |
| LB_pharmacophore11 | 2 | 2 | 3 | 0 | 0 | 0 | 0 | 0 | 0 | 0 | 12 | 120 | 0 |
| LB_pharmacophore12 | 0 | 5 | 1 | 0 | 0 | 0 | 0 | 0 | 0 | 0 | 12 | 121 | 0 |
| LB_pharmacophore13 | 1 | 3 | 2 | 0 | 0 | 1 | 0 | 0 | 0 | 0 | 8 | 125 | 0 |
| LB_pharmacophore14 | 1 | 2 | 1 | 1 | 0 | 0 | 0 | 0 | 0 | 0 | 6 | 128 | 0 |
| LB_pharmacophore15 | 2 | 2 | 5 | 0 | 0 | 1 | 0 | 0 | 0 | 0 | 2 | 129 | 0 |

Table S31. RAR_alpha agonist dataset. Pharmacophore features composition of the SBLB agonist selective pharmacophores (AR: aromatic ring, H:hydrophobic, HBA: Hydrogen Bond Acceptor, HBD: Hydrogen Bond Donor, PI: Positive ionizable area, NI: negative ionisable area, opt.: feature set as optional) and number of agonist ligands found with each pharmacophore (“Nbr of agonist ligands found”), number of unique agonist ligands found by combining the pharmacophores (“Accumulated nbr of unique agonist ligands found”) and number of decoys found (“Nbr of antagonist ligands found”)

|  | Pharmacophore features | | | | | | | | | | Number of positive and negative hits | | |
| --- | --- | --- | --- | --- | --- | --- | --- | --- | --- | --- | --- | --- | --- |
|  | AR | H | HBA | HBD | PI | NI | AR opt. | H opt. | HBA opt. | HBD opt. | Nbr of antagonist ligands found | Accumulated nbr of unique antagonist ligands found | Nbr of agonist ligands found |
| LB_pharmacophore1 | 2 | 0 | 1 | 0 | 0 | 1 | 0 | 0 | 0 | 0 | 28 | 28 | 0 |
| SB_pharmacophore2 | 0 | 3 | 3 | 0 | 0 | 1 | 0 | 0 | 0 | 0 | 26 | 39 | 0 |
| LB_pharmacophore3 | 1 | 3 | 2 | 0 | 0 | 1 | 0 | 0 | 0 | 0 | 22 | 54 | 0 |
| LB_pharmacophore4 | 1 | 6 | 2 | 0 | 0 | 1 | 0 | 0 | 0 | 0 | 14 | 56 | 0 |
| LB_pharmacophore5 | 2 | 5 | 3 | 0 | 0 | 1 | 0 | 0 | 0 | 0 | 4 | 58 | 0 |
| LB_pharmacophore6 | 1 | 1 | 4 | 0 | 0 | 1 | 0 | 0 | 0 | 0 | 3 | 61 | 0 |
| LB_pharmacophore7 | 4 | 5 | 3 | 1 | 0 | 1 | 0 | 0 | 0 | 0 | 2 | 63 | 0 |
| LB_pharmacophore8 | 1 | 3 | 5 | 0 | 0 | 1 | 0 | 0 | 0 | 0 | 2 | 65 | 0 |
| LB_pharmacophore9 | 2 | 5 | 3 | 1 | 0 | 1 | 0 | 0 | 0 | 0 | 1 | 66 | 0 |

Table S32. RAR_alpha antagonist dataset. Pharmacophore features composition of the SBLB agonist selective pharmacophores (AR: aromatic ring, H:hydrophobic, HBA: Hydrogen Bond Acceptor, HBD: Hydrogen Bond Donor, PI: Positive ionizable area, NI: negative ionisable area, opt.: feature set as optional) and number of antagonist ligands found with each pharmacophore (“Nbr of antagonist ligands found”), number of unique antagonist ligands found by combining the pharmacophores (“Accumulated nbr of unique antagonist ligands found”) and number of decoys found (“Nbr of agonist ligands found”)

|  | Pharmacophore features | | | | | | | | | | Number of positive and negative hits | | |
| --- | --- | --- | --- | --- | --- | --- | --- | --- | --- | --- | --- | --- | --- |
|  | AR | H | HBA | HBD | PI | NI | AR opt. | H opt. | HBA opt. | HBD opt. | Nbr of agonist ligands found | Accumulated nbr of unique agonist ligands found | Nbr of antagonist ligands found |
| LB_pharmacophore1 | 0 | 2 | 2 | 0 | 0 | 1 | 0 | 0 | 0 | 0 | 79 | 79 | 0 |
| LB_pharmacophore2 | 1 | 2 | 0 | 0 | 0 | 0 | 0 | 0 | 0 | 0 | 61 | 103 | 0 |
| LB_pharmacophore3 | 1 | 2 | 1 | 0 | 0 | 0 | 0 | 0 | 0 | 0 | 60 | 110 | 0 |
| LB_pharmacophore4 | 0 | 3 | 1 | 0 | 0 | 1 | 0 | 0 | 0 | 0 | 60 | 114 | 0 |
| LB_pharmacophore5 | 0 | 2 | 2 | 0 | 0 | 1 | 0 | 0 | 0 | 0 | 60 | 121 | 0 |
| LB_pharmacophore6 | 0 | 2 | 2 | 0 | 0 | 0 | 0 | 0 | 0 | 0 | 51 | 122 | 0 |
| LB_pharmacophore7 | 0 | 3 | 1 | 0 | 0 | 1 | 0 | 0 | 0 | 0 | 46 | 126 | 0 |
| LB_pharmacophore8 | 0 | 3 | 1 | 0 | 0 | 1 | 0 | 0 | 0 | 0 | 42 | 129 | 0 |
| LB_pharmacophore9 | 1 | 1 | 1 | 0 | 0 | 0 | 0 | 0 | 1 | 1 | 16 | 130 | 0 |

Table S33. RAR_beta agonist dataset. Pharmacophore features composition of the SBLB agonist selective pharmacophores (AR: aromatic ring, H:hydrophobic, HBA: Hydrogen Bond Acceptor, HBD: Hydrogen Bond Donor, PI: Positive ionizable area, NI: negative ionisable area, opt.: feature set as optional) and number of agonist ligands found with each pharmacophore (“Nbr of agonist ligands found”), number of unique agonist ligands found by combining the pharmacophores (“Accumulated nbr of unique agonist ligands found”) and number of decoys found (“Nbr of antagonist ligands found”)

|  | Pharmacophore features | | | | | | | | | | Number of positive and negative hits | | |
| --- | --- | --- | --- | --- | --- | --- | --- | --- | --- | --- | --- | --- | --- |
|  | AR | H | HBA | HBD | PI | NI | AR opt. | H opt. | HBA opt. | HBD opt. | Nbr of antagonist ligands found | Accumulated nbr of unique antagonist ligands found | Nbr of agonist ligands found |
| LB_pharmacophore1 | 1 | 1 | 1 | 0 | 0 | 1 | 0 | 0 | 0 | 0 | 18 | 18 | 0 |
| LB_pharmacophore2 | 2 | 6 | 2 | 0 | 0 | 1 | 0 | 0 | 0 | 0 | 12 | 24 | 0 |
| LB_pharmacophore3 | 2 | 6 | 3 | 0 | 0 | 1 | 0 | 0 | 0 | 0 | 5 | 25 | 0 |
| LB_pharmacophore4 | 3 | 6 | 3 | 0 | 0 | 1 | 0 | 0 | 0 | 0 | 2 | 27 | 0 |
| LB_pharmacophore5 | 2 | 2 | 3 | 0 | 0 | 1 | 0 | 0 | 0 | 0 | 2 | 29 | 0 |
| LB_pharmacophore6 | 5 | 3 | 3 | 1 | 0 | 1 | 0 | 0 | 0 | 0 | 1 | 30 | 0 |
| LB_pharmacophore7 | 4 | 6 | 3 | 1 | 0 | 1 | 0 | 0 | 0 | 0 | 1 | 31 | 0 |

Table S34. RAR_beta antagonist dataset. Pharmacophore features composition of the SBLB agonist selective pharmacophores (AR: aromatic ring, H:hydrophobic, HBA: Hydrogen Bond Acceptor, HBD: Hydrogen Bond Donor, PI: Positive ionizable area, NI: negative ionisable area, opt.: feature set as optional) and number of antagonist ligands found with each pharmacophore (“Nbr of antagonist ligands found”), number of unique antagonist ligands found by combining the pharmacophores (“Accumulated nbr of unique antagonist ligands found”) and number of decoys found (“Nbr of agonist ligands found”)

|  | Pharmacophore features | | | | | | | | | | Number of positive and negative hits | | |
| --- | --- | --- | --- | --- | --- | --- | --- | --- | --- | --- | --- | --- | --- |
|  | AR | H | HBA | HBD | PI | NI | AR opt. | H opt. | HBA opt. | HBD opt. | Nbr of agonist ligands found | Accumulated nbr of unique agonist ligands found | Nbr of antagonist ligands found |
| LB_pharmacophore1 | 1 | 2 | 1 | 0 | 0 | 1 | 0 | 0 | 0 | 0 | 32 | 32 | 0 |
| LB_pharmacophore2 | 1 | 1 | 1 | 0 | 0 | 1 | 0 | 0 | 0 | 0 | 27 | 43 | 0 |
| LB_pharmacophore3 | 2 | 6 | 2 | 0 | 0 | 1 | 0 | 1 | 0 | 0 | 10 | 49 | 0 |
| LB_pharmacophore4 | 1 | 2 | 2 | 0 | 0 | 1 | 0 | 0 | 0 | 0 | 10 | 50 | 0 |
| LB_pharmacophore5 | 3 | 6 | 2 | 0 | 0 | 1 | 0 | 0 | 0 | 0 | 9 | 51 | 0 |
| LB_pharmacophore6 | 3 | 1 | 0 | 0 | 0 | 0 | 0 | 0 | 0 | 0 | 5 | 52 | 0 |
| LB_pharmacophore7 | 3 | 5 | 3 | 0 | 0 | 1 | 0 | 0 | 0 | 1 | 3 | 53 | 0 |
| LB_pharmacophore8 | 3 | 6 | 3 | 1 | 0 | 1 | 0 | 1 | 0 | 0 | 2 | 54 | 0 |
| LB_pharmacophore9 | 2 | 3 | 3 | 1 | 0 | 1 | 0 | 0 | 0 | 0 | 1 | 55 | 0 |
| LB_pharmacophore10 | 4 | 5 | 4 | 1 | 0 | 1 | 0 | 0 | 0 | 0 | 1 | 56 | 0 |
| LB_pharmacophore11 | 2 | 2 | 4 | 0 | 0 | 1 | 0 | 0 | 0 | 0 | 1 | 57 | 0 |

Table S35. RAR_gamma agonist dataset. Pharmacophore features composition of the SBLB agonist selective pharmacophores (AR: aromatic ring, H:hydrophobic, HBA: Hydrogen Bond Acceptor, HBD: Hydrogen Bond Donor, PI: Positive ionizable area, NI: negative ionisable area, opt.: feature set as optional) and number of agonist ligands found with each pharmacophore (“Nbr of agonist ligands found”), number of unique agonist ligands found by combining the pharmacophores (“Accumulated nbr of unique agonist ligands found”) and number of decoys found (“Nbr of antagonist ligands found”)

|  | Pharmacophore features | | | | | | | | | | Number of positive and negative hits | | |
| --- | --- | --- | --- | --- | --- | --- | --- | --- | --- | --- | --- | --- | --- |
|  | AR | H | HBA | HBD | PI | NI | AR opt. | H opt. | HBA opt. | HBD opt. | Nbr of antagonist ligands found | Accumulated nbr of unique antagonist ligands found | Nbr of agonist ligands found |
| LB_pharmacophore1 | 1 | 2 | 1 | 0 | 0 | 1 | 0 | 0 | 0 | 0 | 32 | 32 | 0 |
| LB_pharmacophore2 | 1 | 1 | 1 | 0 | 0 | 1 | 0 | 0 | 0 | 0 | 27 | 43 | 0 |
| LB_pharmacophore3 | 2 | 6 | 2 | 0 | 0 | 1 | 0 | 1 | 0 | 0 | 10 | 49 | 0 |
| LB_pharmacophore4 | 1 | 2 | 2 | 0 | 0 | 1 | 0 | 0 | 0 | 0 | 10 | 50 | 0 |
| LB_pharmacophore5 | 3 | 6 | 2 | 0 | 0 | 1 | 0 | 0 | 0 | 0 | 9 | 51 | 0 |
| LB_pharmacophore6 | 3 | 1 | 0 | 0 | 0 | 0 | 0 | 0 | 0 | 0 | 5 | 52 | 0 |
| LB_pharmacophore7 | 3 | 5 | 3 | 0 | 0 | 1 | 0 | 0 | 0 | 1 | 3 | 53 | 0 |
| LB_pharmacophore8 | 3 | 6 | 3 | 1 | 0 | 1 | 0 | 1 | 0 | 0 | 2 | 54 | 0 |
| LB_pharmacophore9 | 2 | 3 | 3 | 1 | 0 | 1 | 0 | 0 | 0 | 0 | 1 | 55 | 0 |
| LB_pharmacophore10 | 4 | 5 | 4 | 1 | 0 | 1 | 0 | 0 | 0 | 0 | 1 | 56 | 0 |
| LB_pharmacophore11 | 2 | 2 | 4 | 0 | 0 | 1 | 0 | 0 | 0 | 0 | 1 | 57 | 0 |

Table S36. RAR_gamma antagonist dataset. Pharmacophore features composition of the SBLB agonist selective pharmacophores (AR: aromatic ring, H:hydrophobic, HBA: Hydrogen Bond Acceptor, HBD: Hydrogen Bond Donor, PI: Positive ionizable area, NI: negative ionisable area, opt.: feature set as optional) and number of antagonist ligands found with each pharmacophore (“Nbr of antagonist ligands found”), number of unique antagonist ligands found by combining the pharmacophores (“Accumulated nbr of unique antagonist ligands found”) and number of decoys found (“Nbr of agonist ligands found”)

|  | Pharmacophore features | | | | | | | | | | Number of positive and negative hits | | |
| --- | --- | --- | --- | --- | --- | --- | --- | --- | --- | --- | --- | --- | --- |
|  | AR | H | HBA | HBD | PI | NI | AR opt. | H opt. | HBA opt. | HBD opt. | Nbr of agonist ligands found | Accumulated nbr of unique agonist ligands found | Nbr of antagonist ligands found |
| LB_pharmacophore1 | 0 | 2 | 4 | 0 | 0 | 0 | 0 | 0 | 0 | 0 | 2 | 2 | 0 |
| LB_pharmacophore2 | 0 | 2 | 1 | 1 | 0 | 0 | 0 | 0 | 0 | 0 | 2 | 3 | 0 |

Table S37. ROR_alpha agonist dataset. Pharmacophore features composition of the SBLB agonist selective pharmacophores (AR: aromatic ring, H:hydrophobic, HBA: Hydrogen Bond Acceptor, HBD: Hydrogen Bond Donor, PI: Positive ionizable area, NI: negative ionisable area, opt.: feature set as optional) and number of agonist ligands found with each pharmacophore (“Nbr of agonist ligands found”), number of unique agonist ligands found by combining the pharmacophores (“Accumulated nbr of unique agonist ligands found”) and number of decoys found (“Nbr of antagonist ligands found”)

|  | Pharmacophore features | | | | | | | | | | Number of positive and negative hits | | |
| --- | --- | --- | --- | --- | --- | --- | --- | --- | --- | --- | --- | --- | --- |
|  | AR | H | HBA | HBD | PI | NI | AR opt. | H opt. | HBA opt. | HBD opt. | Nbr of antagonist ligands found | Accumulated nbr of unique antagonist ligands found | Nbr of agonist ligands found |
| LB_pharmacophore1 | 0 | 2 | 1 | 1 | 0 | 0 | 0 | 0 | 0 | 0 | 7 | 7 | 0 |

Table S38. ROR_alpha antagonist dataset. Pharmacophore features composition of the SBLB agonist selective pharmacophores (AR: aromatic ring, H:hydrophobic, HBA: Hydrogen Bond Acceptor, HBD: Hydrogen Bond Donor, PI: Positive ionizable area, NI: negative ionisable area, opt.: feature set as optional) and number of antagonist ligands found with each pharmacophore (“Nbr of antagonist ligands found”), number of unique antagonist ligands found by combining the pharmacophores (“Accumulated nbr of unique antagonist ligands found”) and number of decoys found (“Nbr of agonist ligands found”)

|  | Pharmacophore features | | | | | | | | | | Number of positive and negative hits | | |
| --- | --- | --- | --- | --- | --- | --- | --- | --- | --- | --- | --- | --- | --- |
|  | AR | H | HBA | HBD | PI | NI | AR opt. | H opt. | HBA opt. | HBD opt. | Nbr of agonist ligands found | Accumulated nbr of unique agonist ligands found | Nbr of antagonist ligands found |
| LB_pharmacophore1 | 0 | 2 | 1 | 1 | 0 | 0 | 0 | 0 | 0 | 0 | 4 | 4 | 0 |

Table S39. ROR_gamma agonist dataset. Pharmacophore features composition of the SBLB agonist selective pharmacophores (AR: aromatic ring, H:hydrophobic, HBA: Hydrogen Bond Acceptor, HBD: Hydrogen Bond Donor, PI: Positive ionizable area, NI: negative ionisable area, opt.: feature set as optional) and number of agonist ligands found with each pharmacophore (“Nbr of agonist ligands found”), number of unique agonist ligands found by combining the pharmacophores (“Accumulated nbr of unique agonist ligands found”) and number of decoys found (“Nbr of antagonist ligands found”)

|  | Pharmacophore features | | | | | | | | | | Number of positive and negative hits | | |
| --- | --- | --- | --- | --- | --- | --- | --- | --- | --- | --- | --- | --- | --- |
|  | AR | H | HBA | HBD | PI | NI | AR opt. | H opt. | HBA opt. | HBD opt. | Nbr of antagonist ligands found | Accumulated nbr of unique antagonist ligands found | Nbr of agonist ligands found |
| LB_pharmacophore1 | 0 | 2 | 1 | 1 | 0 | 0 | 0 | 0 | 0 | 0 | 4 | 4 | 0 |

Table S40. ROR_gamma antagonist dataset. Pharmacophore features composition of the SBLB agonist selective pharmacophores (AR: aromatic ring, H:hydrophobic, HBA: Hydrogen Bond Acceptor, HBD: Hydrogen Bond Donor, PI: Positive ionizable area, NI: negative ionisable area, opt.: feature set as optional) and number of antagonist ligands found with each pharmacophore (“Nbr of antagonist ligands found”), number of unique antagonist ligands found by combining the pharmacophores (“Accumulated nbr of unique antagonist ligands found”) and number of decoys found (“Nbr of agonist ligands found”)

|  | Pharmacophore features | | | | | | | | | | Number of positive and negative hits | | |
| --- | --- | --- | --- | --- | --- | --- | --- | --- | --- | --- | --- | --- | --- |
|  | AR | H | HBA | HBD | PI | NI | AR opt. | H opt. | HBA opt. | HBD opt. | Nbr of agonist ligands found | Accumulated nbr of unique agonist ligands found | Nbr of antagonist ligands found |
| SB_pharmacophore1 | 0 | 3 | 1 | 0 | 0 | 1 | 0 | 0 | 0 | 0 | 78 | 78 | 0 |
| LB_pharmacophore2 | 0 | 3 | 1 | 0 | 0 | 1 | 0 | 0 | 0 | 0 | 71 | 116 | 0 |
| LB_pharmacophore3 | 0 | 2 | 2 | 0 | 0 | 1 | 0 | 0 | 0 | 0 | 71 | 133 | 0 |
| SB_pharmacophore4 | 0 | 3 | 1 | 0 | 0 | 1 | 0 | 0 | 0 | 0 | 68 | 152 | 0 |
| SB_pharmacophore5 | 0 | 3 | 2 | 0 | 0 | 1 | 0 | 0 | 0 | 0 | 66 | 158 | 0 |
| SB_pharmacophore6 | 0 | 3 | 1 | 0 | 0 | 1 | 0 | 0 | 0 | 0 | 65 | 163 | 0 |
| SB_pharmacophore7 | 0 | 4 | 0 | 0 | 0 | 1 | 0 | 0 | 0 | 0 | 63 | 167 | 0 |
| SB_pharmacophore8 | 0 | 3 | 1 | 0 | 0 | 0 | 0 | 0 | 0 | 0 | 61 | 173 | 0 |
| SB_pharmacophore9 | 0 | 2 | 1 | 0 | 0 | 1 | 0 | 0 | 0 | 0 | 58 | 176 | 0 |
| LB_pharmacophore10 | 0 | 3 | 1 | 0 | 0 | 0 | 0 | 0 | 0 | 0 | 58 | 183 | 0 |
| SB_pharmacophore11 | 0 | 3 | 1 | 0 | 0 | 1 | 0 | 0 | 0 | 0 | 57 | 185 | 0 |
| LB_pharmacophore12 | 1 | 1 | 2 | 0 | 0 | 1 | 0 | 0 | 0 | 0 | 54 | 188 | 0 |
| LB_pharmacophore13 | 0 | 6 | 2 | 0 | 0 | 1 | 0 | 0 | 0 | 0 | 53 | 195 | 0 |
| LB_pharmacophore14 | 0 | 3 | 0 | 0 | 0 | 1 | 0 | 0 | 0 | 0 | 53 | 201 | 0 |
| SB_pharmacophore15 | 0 | 3 | 1 | 0 | 0 | 1 | 0 | 0 | 0 | 0 | 48 | 202 | 0 |
| LB_pharmacophore16 | 1 | 4 | 1 | 0 | 0 | 0 | 0 | 0 | 0 | 0 | 41 | 203 | 0 |
| LB_pharmacophore17 | 2 | 2 | 1 | 0 | 0 | 0 | 0 | 0 | 0 | 0 | 40 | 209 | 0 |
| LB_pharmacophore18 | 2 | 1 | 2 | 0 | 0 | 0 | 0 | 0 | 0 | 0 | 2 | 210 | 0 |

Table S41. RXR_alpha agonist dataset. Pharmacophore features composition of the SBLB agonist selective pharmacophores (AR: aromatic ring, H:hydrophobic, HBA: Hydrogen Bond Acceptor, HBD: Hydrogen Bond Donor, PI: Positive ionizable area, NI: negative ionisable area, opt.: feature set as optional) and number of agonist ligands found with each pharmacophore (“Nbr of agonist ligands found”), number of unique agonist ligands found by combining the pharmacophores (“Accumulated nbr of unique agonist ligands found”) and number of decoys found (“Nbr of antagonist ligands found”)

|  | Pharmacophore features | | | | | | | | | | Number of positive and negative hits | | |
| --- | --- | --- | --- | --- | --- | --- | --- | --- | --- | --- | --- | --- | --- |
|  | AR | H | HBA | HBD | PI | NI | AR opt. | H opt. | HBA opt. | HBD opt. | Nbr of antagonist ligands found | Accumulated nbr of unique antagonist ligands found | Nbr of agonist ligands found |
| LB_pharmacophore1 | 1 | 0 | 3 | 0 | 0 | 0 | 0 | 0 | 0 | 0 | 77 | 77 | 0 |
| LB_pharmacophore2 | 1 | 0 | 2 | 0 | 0 | 1 | 0 | 0 | 0 | 0 | 29 | 105 | 0 |
| LB_pharmacophore3 | 1 | 5 | 3 | 0 | 0 | 1 | 0 | 0 | 0 | 0 | 22 | 116 | 0 |
| LB_pharmacophore4 | 1 | 4 | 3 | 0 | 0 | 1 | 0 | 0 | 0 | 0 | 20 | 120 | 0 |
| LB_pharmacophore5 | 2 | 6 | 1 | 0 | 0 | 0 | 0 | 0 | 0 | 0 | 20 | 121 | 0 |
| LB_pharmacophore6 | 1 | 1 | 0 | 0 | 0 | 1 | 0 | 0 | 0 | 0 | 15 | 123 | 0 |
| LB_pharmacophore7 | 1 | 3 | 1 | 0 | 0 | 0 | 0 | 0 | 0 | 0 | 8 | 124 | 0 |
| LB_pharmacophore8 | 1 | 2 | 2 | 0 | 0 | 0 | 0 | 0 | 0 | 0 | 5 | 125 | 0 |
| LB_pharmacophore9 | 1 | 3 | 2 | 0 | 0 | 0 | 0 | 0 | 0 | 0 | 4 | 127 | 0 |
| LB_pharmacophore10 | 2 | 1 | 1 | 0 | 0 | 0 | 0 | 0 | 0 | 0 | 4 | 129 | 0 |
| LB_pharmacophore11 | 1 | 5 | 4 | 0 | 0 | 1 | 0 | 1 | 2 | 1 | 2 | 130 | 0 |
| SB_pharmacophore12 | 0 | 5 | 6 | 1 | 0 | 1 | 0 | 0 | 0 | 0 | 1 | 131 | 0 |
| LB_pharmacophore13 | 1 | 2 | 1 | 0 | 0 | 0 | 0 | 0 | 0 | 0 | 1 | 132 | 0 |

Table S42. RXR_alpha antagonist dataset. Pharmacophore features composition of the SBLB agonist selective pharmacophores (AR: aromatic ring, H:hydrophobic, HBA: Hydrogen Bond Acceptor, HBD: Hydrogen Bond Donor, PI: Positive ionizable area, NI: negative ionisable area, opt.: feature set as optional) and number of antagonist ligands found with each pharmacophore (“Nbr of antagonist ligands found”), number of unique antagonist ligands found by combining the pharmacophores (“Accumulated nbr of unique antagonist ligands found”) and number of decoys found (“Nbr of agonist ligands found”)

|  | Pharmacophore features | | | | | | | | | | Number of positive and negative hits | | |
| --- | --- | --- | --- | --- | --- | --- | --- | --- | --- | --- | --- | --- | --- |
|  | AR | H | HBA | HBD | PI | NI | AR opt. | H opt. | HBA opt. | HBD opt. | Nbr of agonist ligands found | Accumulated nbr of unique agonist ligands found | Nbr of antagonist ligands found |
| LB_pharmacophore1 | 0 | 2 | 2 | 0 | 0 | 1 | 0 | 0 | 0 | 0 | 55 | 55 | 0 |
| LB_pharmacophore2 | 0 | 2 | 1 | 0 | 0 | 1 | 0 | 0 | 0 | 0 | 48 | 58 | 0 |
| LB_pharmacophore3 | 0 | 3 | 1 | 0 | 0 | 0 | 0 | 0 | 0 | 0 | 45 | 61 | 0 |
| SB_pharmacopore4 | 0 | 4 | 0 | 0 | 0 | 0 | 0 | 0 | 0 | 0 | 28 | 62 | 0 |
| SB_pharmacopore5 | 0 | 3 | 1 | 0 | 0 | 0 | 0 | 0 | 0 | 0 | 25 | 63 | 0 |
| LB_pharmacophore6 | 0 | 5 | 2 | 0 | 0 | 1 | 0 | 0 | 0 | 0 | 22 | 65 | 0 |

Table S43. RXR_beta agonist dataset. Pharmacophore features composition of the SBLB agonist selective pharmacophores (AR: aromatic ring, H:hydrophobic, HBA: Hydrogen Bond Acceptor, HBD: Hydrogen Bond Donor, PI: Positive ionizable area, NI: negative ionisable area, opt.: feature set as optional) and number of agonist ligands found with each pharmacophore (“Nbr of agonist ligands found”), number of unique agonist ligands found by combining the pharmacophores (“Accumulated nbr of unique agonist ligands found”) and number of decoys found (“Nbr of antagonist ligands found”)

|  | Pharmacophore features | | | | | | | | | | Number of positive and negative hits | | |
| --- | --- | --- | --- | --- | --- | --- | --- | --- | --- | --- | --- | --- | --- |
|  | AR | H | HBA | HBD | PI | NI | AR opt. | H opt. | HBA opt. | HBD opt. | Nbr of antagonist ligands found | Accumulated nbr of unique antagonist ligands found | Nbr of agonist ligands found |
| LB_pharmacophore1 | 1 | 5 | 3 | 0 | 0 | 1 | 0 | 0 | 0 | 0 | 5 | 5 | 0 |
| LB_pharmacophore2 | 1 | 4 | 3 | 0 | 0 | 1 | 1 | 0 | 0 | 0 | 2 | 6 | 0 |
| LB_pharmacophore3 | 1 | 5 | 1 | 0 | 0 | 0 | 1 | 0 | 0 | 0 | 2 | 7 | 0 |

Table S44. RXR_beta antagonist dataset. Pharmacophore features composition of the SBLB agonist selective pharmacophores (AR: aromatic ring, H:hydrophobic, HBA: Hydrogen Bond Acceptor, HBD: Hydrogen Bond Donor, PI: Positive ionizable area, NI: negative ionisable area, opt.: feature set as optional) and number of antagonist ligands found with each pharmacophore (“Nbr of antagonist ligands found”), number of unique antagonist ligands found by combining the pharmacophores (“Accumulated nbr of unique antagonist ligands found”) and number of decoys found (“Nbr of agonist ligands found”)

|  | Pharmacophore features | | | | | | | | | | Number of positive and negative hits | | |
| --- | --- | --- | --- | --- | --- | --- | --- | --- | --- | --- | --- | --- | --- |
|  | AR | H | HBA | HBD | PI | NI | AR opt. | H opt. | HBA opt. | HBD opt. | Nbr of agonist ligands found | Accumulated nbr of unique agonist ligands found | Nbr of antagonist ligands found |
| LB_pharmacophore1 | 0 | 2 | 2 | 0 | 0 | 1 | 0 | 0 | 0 | 0 | 64 | 64 | 0 |
| LB_pharmacophore2 | 0 | 2 | 1 | 0 | 0 | 1 | 0 | 0 | 0 | 0 | 62 | 71 | 0 |

Table S45. RXR_gamma agonist dataset. Pharmacophore features composition of the SBLB agonist selective pharmacophores (AR: aromatic ring, H:hydrophobic, HBA: Hydrogen Bond Acceptor, HBD: Hydrogen Bond Donor, PI: Positive ionizable area, NI: negative ionisable area, opt.: feature set as optional) and number of agonist ligands found with each pharmacophore (“Nbr of agonist ligands found”), number of unique agonist ligands found by combining the pharmacophores (“Accumulated nbr of unique agonist ligands found”) and number of decoys found (“Nbr of antagonist ligands found”)

|  | Pharmacophore features | | | | | | | | | | Number of positive and negative hits | | |
| --- | --- | --- | --- | --- | --- | --- | --- | --- | --- | --- | --- | --- | --- |
|  | AR | H | HBA | HBD | PI | NI | AR opt. | H opt. | HBA opt. | HBD opt. | Nbr of antagonist ligands found | Accumulated nbr of unique antagonist ligands found | Nbr of agonist ligands found |
| LB_pharmacophore1 | 2 | 1 | 3 | 0 | 0 | 1 | 0 | 1 | 0 | 0 | 6 | 6 | 0 |

Table S46. RXR_gamma antagonist dataset. Pharmacophore features composition of the SBLB agonist selective pharmacophores (AR: aromatic ring, H:hydrophobic, HBA: Hydrogen Bond Acceptor, HBD: Hydrogen Bond Donor, PI: Positive ionizable area, NI: negative ionisable area, opt.: feature set as optional) and number of antagonist ligands found with each pharmacophore (“Nbr of antagonist ligands found”), number of unique antagonist ligands found by combining the pharmacophores (“Accumulated nbr of unique antagonist ligands found”) and number of decoys found (“Nbr of agonist ligands found”)

|  | Pharmacophore features | | | | | | | | | | Number of positive and negative hits | | |
| --- | --- | --- | --- | --- | --- | --- | --- | --- | --- | --- | --- | --- | --- |
|  | AR | H | HBA | HBD | PI | NI | AR opt. | H opt. | HBA opt. | HBD opt. | Nbr of agonist ligands found | Accumulated nbr of unique agonist ligands found | Nbr of antagonist ligands found |
| LB_pharmacophore1 | 2 | 2 | 0 | 0 | 0 | 0 | 0 | 0 | 0 | 0 | 12 | 12 | 0 |
| LB_pharmacophore2 | 1 | 3 | 0 | 0 | 0 | 0 | 0 | 0 | 0 | 0 | 6 | 14 | 0 |
| LB_pharmacophore3 | 0 | 4 | 0 | 1 | 0 | 0 | 0 | 0 | 0 | 0 | 3 | 16 | 0 |
| LB_pharmacophore4 | 1 | 2 | 1 | 0 | 0 | 0 | 0 | 0 | 0 | 0 | 3 | 19 | 0 |

Table S47. SF1 agonist dataset. Pharmacophore features composition of the SBLB agonist selective pharmacophores (AR: aromatic ring, H:hydrophobic, HBA: Hydrogen Bond Acceptor, HBD: Hydrogen Bond Donor, PI: Positive ionizable area, NI: negative ionisable area, opt.: feature set as optional) and number of agonist ligands found with each pharmacophore (“Nbr of agonist ligands found”), number of unique agonist ligands found by combining the pharmacophores (“Accumulated nbr of unique agonist ligands found”) and number of decoys found (“Nbr of antagonist ligands found”)

|  | Pharmacophore features | | | | | | | | | | Number of positive and negative hits | | |
| --- | --- | --- | --- | --- | --- | --- | --- | --- | --- | --- | --- | --- | --- |
|  | AR | H | HBA | HBD | PI | NI | AR opt. | H opt. | HBA opt. | HBD opt. | Nbr of antagonist ligands found | Accumulated nbr of unique antagonist ligands found | Nbr of agonist ligands found |
| LB_pharmacophore1 | 2 | 2 | 3 | 1 | 0 | 0 | 0 | 0 | 0 | 0 | 19 | 19 | 0 |
| LB_pharmacophore2 | 0 | 2 | 2 | 1 | 0 | 0 | 0 | 0 | 2 | 2 | 7 | 20 | 0 |

Table S48. SF1 antagonist dataset. Pharmacophore features composition of the SBLB agonist selective pharmacophores (AR: aromatic ring, H:hydrophobic, HBA: Hydrogen Bond Acceptor, HBD: Hydrogen Bond Donor, PI: Positive ionizable area, NI: negative ionisable area, opt.: feature set as optional) and number of antagonist ligands found with each pharmacophore (“Nbr of antagonist ligands found”), number of unique antagonist ligands found by combining the pharmacophores (“Accumulated nbr of unique antagonist ligands found”) and number of decoys found (“Nbr of agonist ligands found”)

|  | Pharmacophore features | | | | | | | | | | Number of positive and negative hits | | |
| --- | --- | --- | --- | --- | --- | --- | --- | --- | --- | --- | --- | --- | --- |
|  | AR | H | HBA | HBD | PI | NI | AR opt. | H opt. | HBA opt. | HBD opt. | Nbr of agonist ligands found | Accumulated nbr of unique agonist ligands found | Nbr of antagonist ligands found |
| LB_pharmacophore1 | 2 | 1 | 1 | 1 | 0 | 0 | 0 | 0 | 0 | 0 | 58 | 58 | 0 |
| LB_pharmacophore2 | 2 | 1 | 2 | 1 | 0 | 0 | 0 | 0 | 2 | 0 | 56 | 61 | 0 |
| SB_pharmacophore3 | 0 | 4 | 1 | 0 | 0 | 0 | 0 | 0 | 0 | 0 | 46 | 66 | 0 |
| SB_pharmacophore4 | 0 | 2 | 2 | 0 | 0 | 0 | 0 | 0 | 0 | 0 | 25 | 69 | 0 |

Table S49. TR_alpha agonist dataset. Pharmacophore features composition of the SBLB agonist selective pharmacophores (AR: aromatic ring, H:hydrophobic, HBA: Hydrogen Bond Acceptor, HBD: Hydrogen Bond Donor, PI: Positive ionizable area, NI: negative ionisable area, opt.: feature set as optional) and number of agonist ligands found with each pharmacophore (“Nbr of agonist ligands found”), number of unique agonist ligands found by combining the pharmacophores (“Accumulated nbr of unique agonist ligands found”) and number of decoys found (“Nbr of antagonist ligands found”)

|  | Pharmacophore features | | | | | | | | | | Number of positive and negative hits | | |
| --- | --- | --- | --- | --- | --- | --- | --- | --- | --- | --- | --- | --- | --- |
|  | AR | H | HBA | HBD | PI | NI | AR opt. | H opt. | HBA opt. | HBD opt. | Nbr of antagonist ligands found | Accumulated nbr of unique antagonist ligands found | Nbr of agonist ligands found |
| LB_pharmacophore1 | 2 | 5 | 4 | 2 | 0 | 0 | 0 | 0 | 0 | 0 | 4 | 4 | 0 |
| LB_pharmacophore2 | 0 | 2 | 1 | 0 | 0 | 0 | 0 | 0 | 2 | 0 | 4 | 7 | 0 |
| LB_pharmacophore3 | 2 | 4 | 2 | 1 | 0 | 0 | 0 | 0 | 0 | 0 | 3 | 10 | 0 |
| LB_pharmacophore4 | 2 | 4 | 4 | 2 | 1 | 0 | 0 | 0 | 0 | 0 | 3 | 12 | 0 |
| LB_pharmacophore5 | 1 | 3 | 4 | 2 | 0 | 0 | 0 | 0 | 0 | 0 | 3 | 13 | 0 |
| LB_pharmacophore6 | 2 | 2 | 4 | 0 | 0 | 0 | 0 | 0 | 0 | 0 | 2 | 15 | 0 |
| LB_pharmacophore7 | 2 | 3 | 6 | 1 | 0 | 1 | 0 | 0 | 0 | 0 | 1 | 16 | 0 |
| LB_pharmacophore8 | 0 | 0 | 5 | 1 | 0 | 1 | 0 | 0 | 0 | 0 | 1 | 17 | 0 |

Table S50. TR_alpha antagonist dataset. Pharmacophore features composition of the SBLB agonist selective pharmacophores (AR: aromatic ring, H:hydrophobic, HBA: Hydrogen Bond Acceptor, HBD: Hydrogen Bond Donor, PI: Positive ionizable area, NI: negative ionisable area, opt.: feature set as optional) and number of antagonist ligands found with each pharmacophore (“Nbr of antagonist ligands found”), number of unique antagonist ligands found by combining the pharmacophores (“Accumulated nbr of unique antagonist ligands found”) and number of decoys found (“Nbr of agonist ligands found”)

|  | Pharmacophore features | | | | | | | | | | Number of positive and negative hits | | |
| --- | --- | --- | --- | --- | --- | --- | --- | --- | --- | --- | --- | --- | --- |
|  | AR | H | HBA | HBD | PI | NI | AR opt. | H opt. | HBA opt. | HBD opt. | Nbr of agonist ligands found | Accumulated nbr of unique agonist ligands found | Nbr of antagonist ligands found |
| SB_pharmacophore1 | 0 | 2 | 1 | 1 | 0 | 0 | 0 | 0 | 0 | 0 | 51 | 51 | 0 |
| SB_pharmacophore2 | 0 | 4 | 0 | 0 | 0 | 0 | 0 | 0 | 0 | 0 | 51 | 64 | 0 |
| SB_pharmacophore3 | 0 | 3 | 1 | 0 | 0 | 0 | 0 | 0 | 0 | 0 | 50 | 69 | 0 |
| LB_pharmacophore4 | 0 | 1 | 1 | 1 | 0 | 1 | 0 | 0 | 0 | 0 | 44 | 73 | 0 |
| LB_pharmacophore5 | 2 | 2 | 2 | 1 | 0 | 0 | 0 | 0 | 0 | 0 | 27 | 74 | 0 |
| LB_pharmacophore6 | 2 | 3 | 3 | 0 | 0 | 1 | 0 | 0 | 0 | 0 | 2 | 76 | 0 |
| LB_pharmacophore7 | 2 | 3 | 4 | 1 | 0 | 1 | 0 | 0 | 0 | 0 | 2 | 77 | 0 |

Table S51. TR_beta agonist dataset. Pharmacophore features composition of the SBLB agonist selective pharmacophores (AR: aromatic ring, H:hydrophobic, HBA: Hydrogen Bond Acceptor, HBD: Hydrogen Bond Donor, PI: Positive ionizable area, NI: negative ionisable area, opt.: feature set as optional) and number of agonist ligands found with each pharmacophore (“Nbr of agonist ligands found”), number of unique agonist ligands found by combining the pharmacophores (“Accumulated nbr of unique agonist ligands found”) and number of decoys found (“Nbr of antagonist ligands found”)

|  | Pharmacophore features | | | | | | | | | | Number of positive and negative hits | | |
| --- | --- | --- | --- | --- | --- | --- | --- | --- | --- | --- | --- | --- | --- |
|  | AR | H | HBA | HBD | PI | NI | AR opt. | H opt. | HBA opt. | HBD opt. | Nbr of antagonist ligands found | Accumulated nbr of unique antagonist ligands found | Nbr of agonist ligands found |
| LB_pharmacophore1 | 2 | 5 | 4 | 2 | 0 | 0 | 0 | 0 | 0 | 0 | 4 | 4 | 0 |
| LB_pharmacophore2 | 3 | 4 | 3 | 1 | 0 | 1 | 0 | 0 | 0 | 0 | 3 | 7 | 0 |
| LB_pharmacophore3 | 2 | 3 | 2 | 1 | 0 | 0 | 0 | 0 | 0 | 0 | 3 | 10 | 0 |
| LB_pharmacophore4 | 2 | 3 | 5 | 1 | 0 | 1 | 0 | 0 | 1 | 0 | 2 | 11 | 0 |
| LB_pharmacophore5 | 2 | 1 | 3 | 0 | 0 | 1 | 0 | 0 | 3 | 1 | 2 | 13 | 0 |
| LB_pharmacophore6 | 2 | 4 | 4 | 2 | 0 | 0 | 0 | 0 | 0 | 0 | 2 | 14 | 0 |
| LB_pharmacophore7 | 1 | 2 | 4 | 1 | 0 | 1 | 0 | 0 | 0 | 0 | 1 | 15 | 0 |

Table S52. TR_beta antagonist dataset. Pharmacophore features composition of the SBLB agonist selective pharmacophores (AR: aromatic ring, H:hydrophobic, HBA: Hydrogen Bond Acceptor, HBD: Hydrogen Bond Donor, PI: Positive ionizable area, NI: negative ionisable area, opt.: feature set as optional) and number of antagonist ligands found with each pharmacophore (“Nbr of antagonist ligands found”), number of unique antagonist ligands found by combining the pharmacophores (“Accumulated nbr of unique antagonist ligands found”) and number of decoys found (“Nbr of agonist ligands found”)

|  | Pharmacophore features | | | | | | | | | | Number of positive and negative hits | | |
| --- | --- | --- | --- | --- | --- | --- | --- | --- | --- | --- | --- | --- | --- |
|  | AR | H | HBA | HBD | PI | NI | AR opt. | H opt. | HBA opt. | HBD opt. | Nbr of agonist ligands found | Accumulated nbr of unique agonist ligands found | Nbr of antagonist ligands found |
| LB_pharmcophore1 | 0 | 1 | 1 | 2 | 0 | 0 | 0 | 0 | 0 | 0 | 83 | 83 | 0 |
| LB_pharmcophore2 | 0 | 2 | 0 | 2 | 0 | 0 | 0 | 0 | 0 | 0 | 64 | 85 | 0 |
| LB_pharmcophore3 | 0 | 3 | 2 | 1 | 0 | 0 | 0 | 0 | 0 | 0 | 61 | 91 | 0 |
| LB_pharmcophore4 | 0 | 3 | 1 | 0 | 0 | 0 | 0 | 0 | 0 | 0 | 45 | 105 | 0 |
| LB_pharmcophore5 | 0 | 2 | 2 | 1 | 0 | 0 | 0 | 0 | 0 | 0 | 35 | 106 | 0 |
| LB_pharmcophore6 | 0 | 2 | 2 | 2 | 0 | 0 | 0 | 0 | 0 | 0 | 32 | 108 | 0 |
| LB_pharmcophore7 | 0 | 4 | 2 | 2 | 0 | 0 | 0 | 0 | 0 | 0 | 15 | 114 | 0 |
| SB_pharmacophore8 | 0 | 3 | 1 | 0 | 0 | 0 | 0 | 0 | 0 | 0 | 15 | 116 | 0 |
| LB_pharmcophore9 | 0 | 1 | 2 | 0 | 0 | 1 | 0 | 0 | 0 | 0 | 12 | 119 | 0 |
| SB_pharmacophore10 | 0 | 1 | 3 | 4 | 0 | 0 | 0 | 0 | 0 | 0 | 7 | 120 | 0 |
| LB_pharmcophore11 | 2 | 3 | 0 | 0 | 0 | 0 | 0 | 0 | 0 | 0 | 3 | 121 | 0 |

Table S53. VDR agonist dataset. Pharmacophore features composition of the SBLB agonist selective pharmacophores (AR: aromatic ring, H:hydrophobic, HBA: Hydrogen Bond Acceptor, HBD: Hydrogen Bond Donor, PI: Positive ionizable area, NI: negative ionisable area, opt.: feature set as optional) and number of agonist ligands found with each pharmacophore (“Nbr of agonist ligands found”), number of unique agonist ligands found by combining the pharmacophores (“Accumulated nbr of unique agonist ligands found”) and number of decoys found (“Nbr of antagonist ligands found”)

|  | Pharmacophore features | | | | | | | | | | Number of positive and negative hits | | |
| --- | --- | --- | --- | --- | --- | --- | --- | --- | --- | --- | --- | --- | --- |
|  | AR | H | HBA | HBD | PI | NI | AR opt. | H opt. | HBA opt. | HBD opt. | Nbr of antagonist ligands found | Accumulated nbr of unique antagonist ligands found | Nbr of agonist ligands found |
| LB_pharmacophore1 | 0 | 2 | 3 | 1 | 0 | 0 | 0 | 0 | 0 | 0 | 26 | 26 | 0 |
| LB_pharmacophore2 | 0 | 4 | 4 | 3 | 0 | 0 | 0 | 0 | 0 | 0 | 11 | 37 | 0 |
| LB_pharmacophore3 | 0 | 4 | 5 | 3 | 0 | 0 | 0 | 0 | 0 | 0 | 8 | 38 | 0 |
| LB_pharmacophore4 | 0 | 3 | 3 | 1 | 0 | 0 | 0 | 0 | 0 | 0 | 6 | 39 | 0 |
| LB_pharmacophore5 | 0 | 3 | 2 | 2 | 0 | 0 | 0 | 0 | 0 | 1 | 5 | 43 | 0 |
| LB_pharmacophore6 | 0 | 5 | 3 | 3 | 0 | 0 | 0 | 0 | 0 | 0 | 2 | 44 | 0 |
| LB_pharmacophore7 | 0 | 4 | 3 | 3 | 0 | 0 | 0 | 0 | 0 | 0 | 1 | 45 | 0 |

Table S54. VDR antagonist dataset. Pharmacophore features composition of the SBLB agonist selective pharmacophores (AR: aromatic ring, H:hydrophobic, HBA: Hydrogen Bond Acceptor, HBD: Hydrogen Bond Donor, PI: Positive ionizable area, NI: negative ionisable area, opt.: feature set as optional) and number of antagonist ligands found with each pharmacophore (“Nbr of antagonist ligands found”), number of unique antagonist ligands found by combining the pharmacophores (“Accumulated nbr of unique antagonist ligands found”) and number of decoys found (“Nbr of agonist ligands found”)

|  | SB_pharmacophore4 | | | SB_pharmacophore5 | | | SB_pharmacophore9 | | | SB_pharmacophore16 | | | SB_pharmacophore17 | | |  |
| --- | --- | --- | --- | --- | --- | --- | --- | --- | --- | --- | --- | --- | --- | --- | --- | --- |
|  | Overlap (/4) | RMS | Score | Overlap (/5) | RMS | Score | Overlap (/5) | RMS | Score | Overlap (/4) | RMS | Score | Overlap (/5) | RMS | Score | |
| LB_pharmacophore1 | 3 | 0.054 | 38.8 | 4 | 0.279 | 38.2 | 5 | 0.751 | 56.7 | 0 | ND | ND | 4 | 0.176 | 48.5 | |
| LB_pharmacophore2 | 4 | 1.109 | 39.0 | 4 | 0.585 | 37.2 | 5 | 0.193 | 58.4 | 3 | 1.466 | 34.6 | 5 | 0.436 | 51.0 | |
| LB_pharmacophore3 | 4 | 0.351 | 47.9 | 5 | 0.073 | 58.0 | 5 | 0.701 | 56.5 | 0 | ND | ND | 4 | 0.244 | 48.3 | |
| LB_pharmacophore6 | 3 | 0.136 | 38.6 | 4 | 0.149 | 48.6 | 5 | 0.850 | 56.5 | 0 | ND | ND | 0 | ND | ND | |
| LB_pharmacophore7 | 3 | 0.025 | 38.9 | 4 | 0.067 | 43.8 | 5 | 0.534 | 52.4 | 0 | ND | ND | 0 | ND | ND | |
| LB_pharmacophore8 | 3 | 0.103 | 38.7 | 3 | 0.865 | 36.4 | 4 | 0.120 | 48.6 | 0 | ND | ND | 2 | 1.041 | 35.9 | |
| LB_pharmacophore10 | 0 | ND | ND | 0 | ND | ND | 0 | ND | ND | 0 | ND | ND | 3 | 0.859 | 39.8 | |
| LB_pharmacophore11 | 3 | 0.085 | 38.7 | 3 | 0.887 | 36.3 | 4 | 0.103 | 48.7 | 0 | ND | ND | 2 | 1.063 | 35.8 | |
| LB_pharmacophore12 | 4 | 0.897 | 46.3 | 4 | 1.372 | 44.9 | 4 | 0.004 | 49.0 | 0 | ND | ND | 3 | 0.419 | 47.7 | |
| LB_pharmacophore13 | 3 | 0.020 | 35.6 | 0 | ND | ND | 4 | 0.042 | 45.5 | 0 | ND | ND | 0 | ND | ND | |
| LB_pharmacophore14 | 0 | ND | ND | 0 | ND | ND | 4 | 0.048 | 46.6 | 0 | ND | ND | 0 | ND | ND | |
| LB_pharmacophore15 | 3 | 0.052 | 38.8 | 0 | ND | ND | 4 | 0.072 | 48.8 | 0 | ND | ND | 0 | ND | ND | |
| LB_pharmacophore18 | 4 | 0.330 | 48.0 | 4 | 0.314 | 48.1 | 4 | 0.027 | 48.9 | 3 | 1.257 | 35.2 | 4 | 0.496 | 52.5 | |
| LB_pharmacophore19 | 0 | ND | ND | 0 | ND | ND | 0 | ND | ND | 0 | ND | ND | 0 | ND | ND | |
| LB_pharmacophore20 | 4 | 0.295 | 48.1 | 4 | 0.217 | 48.3 | 4 | 0.025 | 48.9 | 0 | ND | ND | 3 | 0.239 | 48.3 | |
| LB_pharmacophore21 | 0 | ND | ND | 0 | ND | ND | 5 | 0.083 | 48.8 | 0 | ND | ND | 3 | 0.555 | 47.3 | |
| LB_pharmacophore22 | 0 | ND | ND | 0 | ND | ND | 0 | ND | ND | 0 | ND | ND | 0 | ND | ND | |
| LB_pharmacophore23 | 0 | ND | ND | 0 | ND | ND | 0 | ND | ND | 0 | ND | ND | 0 | ND | ND | |
| LB_pharmacophore24 | 0 | ND | ND | 0 | ND | ND | 0 | ND | ND | 0 | ND | ND | 0 | ND | ND | |
| LB_pharmacophore25 | 3 | 1.260 | 35.2 | 4 | 1.181 | 45.5 | 0 | ND | ND | 0 | ND | ND | 0 | ND | ND | |
| LB_pharmacophore26 | 0 | ND | ND | 0 | ND | ND | 0 | ND | ND | 0 | ND | ND | 0 | ND | ND | |
| LB_pharmacophore27 | 0 | ND | ND | 0 | ND | ND | 4 | 0.739 | 45.9 | 0 | ND | ND | 0 | ND | ND | |
| LB_pharmacophore28 | 4 | 0.437 | 37.7 | 4 | 0.600 | 37.2 | 4 | 0.252 | 38.2 | 0 | ND | ND | 3 | 0.226 | 34.5 | |
| LB_pharmacophore29 | 4 | 0.925 | 46.2 | 3 | 0.856 | 36.4 | 4 | 0.042 | 48.9 | 0 | ND | ND | 3 | 0.607 | 47.2 | |
| LB_pharmacophore30 | 4 | 1.018 | 43.0 | 4 | 0.826 | 43.6 | 4 | 0.069 | 47.0 | 4 | 0.942 | 43.8 | 3 | 0.523 | 46.3 | |
| LB_pharmacophore31 | 4 | 0.365 | 41.2 | 4 | 0.314 | 44.7 | 5 | 0.894 | 49.7 | 4 | 0.498 | 44.2 | 4 | 0.657 | 53.7 | |
| LB_pharmacophore32 | 3 | 0.18 | 31.4 | 3 | 0.917 | 36.2 | 0 | ND | ND | 0 | ND | ND | 3 | 0.460 | 41.0 | |

Table S55. (continued next page)

|  | SB_pharmacophore4 | | | SB_pharmacophore5 | | | SB_pharmacophore9 | | | SB_pharmacophore16 | | | SB_pharmacophore17 | | |  |
| --- | --- | --- | --- | --- | --- | --- | --- | --- | --- | --- | --- | --- | --- | --- | --- | --- |
|  | Overlap (/4) | RMS | Score | Overlap (/5) | RMS | Score | Overlap (/5) | RMS | Score | Overlap (/4) | RMS | Score | Overlap (/5) | RMS | Score | |
| LB_pharmacophore33 | 4 | 0.617 | 43.8 | 4 | 0.464 | 44.3 | 4 | 0.211 | 48.4 | 0 | ND | ND | 3 | 0.016 | 45.6 | |
| LB_pharmacophore34 | 4 | 0.885 | 46.3 | 0 | ND | ND | 5 | 0.481 | 52.6 | 0 | ND | ND | 3 | 0.415 | 47.8 | |
| LB_pharmacophore35 | 3 | 0.704 | 31.9 | 4 | 1.236 | 45.3 | 4 | 0.644 | 42.1 | 0 | ND | ND | 2 | 1.240 | 30.3 | |
| LB_pharmacophore36 | 4 | 0.116 | 48.7 | 0 | ND | ND | 4 | 0.060 | 48.8 | 0 | ND | ND | 3 | 0.583 | 47.3 | |
| LB_pharmacophore37 | 0 | ND | ND | 0 | ND | ND | 0 | ND | ND | 0 | ND | ND | 0 | ND | ND | |
| LB_pharmacophore38 | 4 | 0.279 | 38.2 | 5 | 0.900 | 51.3 | 5 | 1.192 | 40.4 | 4 | 0.351 | 37.9 | 4 | 0.685 | 41.9 | |
| LB_pharmacophore39 | 0 | ND | ND | 0 | ND | ND | 4 | 0.269 | 48.2 | 0 | ND | ND | 0 | ND | ND | |

Table S55. Overlapping features between ER_alpha the SB agonist selective pharmacophores and the LB agonist selective pharmacophores included in the SBLB agonist combination of pharmacophores (Overlap: number of LB pharmacophore features that overlap SB pharmacophore features, RMS: Root Mean Square of matched features pairs, Score: LigandScout alignment score)

|  | SB_pharmacophore1 | | | SB_pharmacophore3 | | | SB_pharmacophore4 | | | SB_pharmacophore6 | | | SB_pharmacophore7 | | | SB_pharmacophore15 | | |  |
| --- | --- | --- | --- | --- | --- | --- | --- | --- | --- | --- | --- | --- | --- | --- | --- | --- | --- | --- | --- |
|  | Overlap(/7) | RMS | Score | Overlap(/4) | RMS | Score | Overlap(/7) | RMS | Score | Overlap(/5) | RMS | Score | Overlap(/4) | RMS | Score | Overlap(/6) | RMS | Score | |
| LB_pharmacophore2 | 0 | ND | ND | 0 | ND | ND | 0 | ND | ND | 0 | ND | ND | 0 | ND | ND | 0 | ND | ND | |
| LB_pharmacophore5 | 5 | 0.476 | 57.6 | 3 | 0.516 | 37.5 | 3 | 0.100 | 48.7 | 3 | 0.988 | 36.0 | 3 | 1.083 | 35.8 | 0 | ND | ND | |
| LB_pharmacophore8 | 4 | 0.24 | 48.0 | 0 | ND | ND | 0 | ND | ND | 0 | ND | ND | 0 | ND | ND | 3 | 0.805 | 35.2 | |
| LB_pharmacophore9 | 0 | ND | ND | 0 | ND | ND | 0 | ND | ND | 0 | ND | ND | 0 | ND | ND | 0 | ND | ND | |
| LB_pharmacophore10 | 5 | 0.269 | 54.9 | 0 | ND | ND | 4 | 0.270 | 54.9 | 4 | 1.114 | 42.3 | 3 | 0.276 | 35.9 | 0 | ND | ND | |
| LB_pharmacophore11 | 0 | ND | ND | 0 | ND | ND | 0 | ND | ND | 0 | ND | ND | 0 | ND | ND | 3 | 0.777 | 36.7 | |
| LB_pharmacophore12 | 5 | 0.839 | 53.1 | 3 | 0.361 | 34.6 | 3 | 0.766 | 43.4 | 0 | ND | ND | 0 | ND | ND | 3 | 0.780 | 33.3 | |
| LB_pharmacophore13 | 6 | 0.698 | 61.9 | 3 | 0.604 | 32.2 | 4 | 0.121 | 53.6 | 4 | 1.054 | 40.8 | 3 | 0.361 | 32.9 | 3 | 0.838 | 31.5 | |
| LB_pharmacophore14 | 6 | 0.536 | 67.4 | 3 | 0.884 | 36.3 | 5 | 0.243 | 68.3 | 4 | 1.041 | 45.9 | 3 | 0.189 | 38.4 | 3 | 0.249 | 38.3 | |
| LB_pharmacophore16 | 5 | 0.504 | 57.5 | 0 | ND | ND | 3 | 1.238 | 45.3 | 3 | 1.000 | 36.0 | 0 | ND | ND | 0 | ND | ND | |
| LB_pharmacophore17 | 6 | 0.602 | 67.2 | 3 | 0.920 | 36.2 | 4 | 0.238 | 58.3 | 4 | 0.875 | 46.4 | 3 | 0.215 | 38.4 | 4 | 0.988 | 46.0 | |
| LB_pharmacophore18 | 6 | 0.437 | 67.7 | 0 | ND | ND | 5 | 0.578 | 67.3 | 4 | 0.745 | 46.8 | 3 | 0.226 | 38.3 | 4 | 1.210 | 45.4 | |
| LB_pharmacophore19 | 5 | 0.121 | 58.6 | 0 | ND | ND | 4 | 0.829 | 56.5 | 3 | 0.953 | 36.1 | 3 | 0.904 | 36.3 | 0 | ND | ND | |
| LB_pharmacophore20 | 5 | 0.521 | 57.4 | 0 | ND | ND | 3 | 1.232 | 45.3 | 3 | 1.01 | 36.0 | 0 | ND | ND | 0 | ND | ND | |
| LB_pharmacophore21 | 6 | 0.536 | 67.4 | 3 | 0.283 | 38.2 | 5 | 0.243 | 68.3 | 4 | 1.041 | 45.9 | 3 | 0.189 | 38.4 | 4 | 0.822 | 46.5 | |
| LB_pharmacophore22 | 6 | 0.617 | 67.1 | 3 | 0.596 | 37.2 | 3 | 0.606 | 47.2 | 4 | 1.200 | 45.4 | 3 | 0.209 | 38.4 | 3 | 0.662 | 37.0 | |
| LB_pharmacophore23 | 5 | 0.435 | 57.7 | 0 | ND | ND | 3 | 0.475 | 47.6 | 3 | 0.965 | 36.1 | 3 | 1.084 | 35.7 | 3 | 1.254 | 35.2 | |

Table S56. Overlapping features between ER_alpha the SB antagonist selective pharmacophores and the LB antagonist selective pharmacophores included in the SBLB antagonist combination of pharmacophores (Overlap: number of LB pharmacophore features that overlap SB pharmacophore features, RMS: Root Mean Square of matched features pairs, Score: LigandScout alignment score)
